# ACE2 Netlas: *In silico* Functional Characterization and Drug-Gene Interactions of *ACE2* Gene Network to Understand Its Potential Involvement in COVID-19 Susceptibility

**DOI:** 10.3389/fgene.2021.698033

**Published:** 2021-08-27

**Authors:** Gita A. Pathak, Frank R. Wendt, Aranyak Goswami, Dora Koller, Flavio De Angelis, COVID-19 Host Genetics Initiative, Renato Polimanti

**Affiliations:** ^1^Division of Human Genetics, Department of Psychiatry, Yale School of Medicine, New Haven, CT, United States; ^2^Veteran Affairs Connecticut Healthcare System, West Haven, CT, United States

**Keywords:** ACE2, COVID-19, miRNA, immune response, network

## Abstract

Angiotensin-converting enzyme-2 (*ACE2*) receptor has been identified as the key adhesion molecule for the transmission of the SARS-CoV-2. However, there is no evidence that human genetic variation in ACE2 is singularly responsible for COVID-19 susceptibility. Therefore, we performed an integrative multi-level characterization of genes that interact with ACE2 (ACE2-gene network) for their statistically enriched biological properties in the context of COVID-19. The phenome-wide association of 51 genes including ACE2 with 4,756 traits categorized into 26 phenotype categories, showed enrichment of immunological, respiratory, environmental, skeletal, dermatological, and metabolic domains (*p* < 4e-4). Transcriptomic regulation of ACE2-gene network was enriched for tissue-specificity in kidney, small intestine, and colon (*p* < 4.7e-4). Leveraging the drug-gene interaction database we identified 47 drugs, including dexamethasone and spironolactone, among others. Considering genetic variants within ± 10 kb of ACE2-network genes we identified miRNAs whose binding sites may be altered as a consequence of genetic variation. The identified miRNAs revealed statistical over-representation of inflammation, aging, diabetes, and heart conditions. The genetic variant associations in *RORA*, *SLC12A6*, and *SLC6A19* genes were observed in genome-wide association study (GWAS) of COVID-19 susceptibility. We also report the GWAS-identified variant in 3p21.31 locus, serves as trans-QTL for *RORA* and *RORC* genes. Overall, functional characterization of ACE2-gene network highlights several potential mechanisms in COVID-19 susceptibility. The data can also be accessed at https://gpwhiz.github.io/ACE2Netlas/.

## Introduction

SARS-CoV-2 (severe acute respiratory syndrome coronavirus 2) is the causative agent responsible for recent global spread of COVID-19 (coronavirus disease 2019) (Wu et al., 2020; Zhou et al., 2020). Millions of people have been infected with the virus, which caused global lockdowns and heavily restricted interpersonal contact. These measures were taken to reduce viral spread through respiratory droplet exchange between persons.

SARS-CoV-2 is capable of entering the host cells via ACE2 (angiotensin converting enzyme 2) (Walls et al., 2020; Mercurio et al., 2021). ACE2 is found on many different cell types, which normally helps regulate blood pressure and inflammation through cleavage of angiotensin II (ANG II) (Hamming et al., 2007). The virus occupies cell-surface of *ACE2* leading to accumulation of angiotensin (ANGII), inflammation, and cell death (Walls et al., 2020). The interactions between the spike protein and ACE2 trigger pre-/post-fusion conformational changes at the spike protein, following spike cleavage in two main domains. The spike N-terminal domain forms a stable protein-protein complex with ACE2, whereas the C-terminal domain, namely the spike post-fusion protein, favors virus/host-cells membrane fusion (Mercurio et al., 2021). In the lungs, SARS-CoV-2 mediated ANGII accumulation leads to alveolar cell death and a reduction in oxygen uptake (Wang et al., 2020).

Although ACE2 is the cellular entry point, there is little evidence that genetic variation in *ACE2* is singularly responsible for COVID-19 susceptibility (Ellinghaus et al., 2020; Pairo-Castineira et al., 2020; Shelton et al., 2020). Due to the functional role of ACE2 in SARS-CoV-2 infection, we hypothesize that genes interacting with ACE2 activity are enriched for molecular pathways relevant for COVID-19 susceptibility. Accordingly, we employed a top-down approach to analyze tissue-specific transcriptomic regulation, drug-gene interactions, and variant prioritization using genetic variants within the ACE2 gene-gene connectome and protein-protein interaction networks. With this approach we identified several biological processes and functional effects of ACE2-gene network relevant for the vast symptoms observed following SARS-CoV-2 infection.

## Results

A study overview is presented in Supplementary Figure 1.

### The ACE2 Gene Connectome

A total of 60 genes were identified from six network databases that interact with *ACE2* (Supplementary Table 1).

### Tissue-Specific Transcriptomic Regulation

**FIGURE 1:**
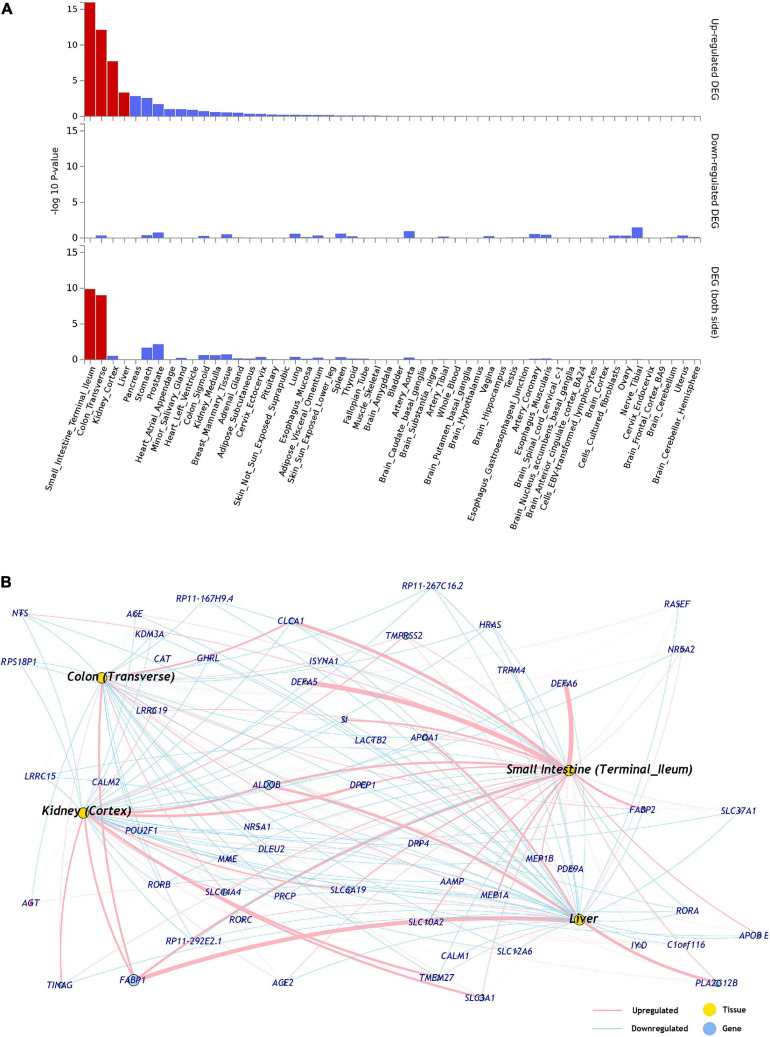
**(A)** Tissues enriched based on ACE2-network gene expression from GTEx database. The genes from the ACE2-network show over-representation of tissues (*x*-axis) and –log10 *p*-value (*y*-axis). The red bars are significant enrichments. (DEG—Differentially expressed genes). **(B)** The network of genes with tissue specific expression of overrepresented tissues, pink edges represent upregulation and blue edges represent downregulation. The weight of the edges corresponds to expression values (average transcript per million; TPM).

The differential expression data of 54 tissues (GTEx-v8) was used to identify the tissue specificity of the ACE2 network genes. The ACE2 network genes were enriched for upregulated expression in small intestine (*p* = 1.07 × 10^–16^), colon (*p* = 7.60 × 10^–13^), kidney (*p* = 1.93 × 10^–8^), and liver (*p* = 4.63 × 10^–4^) (Figure 1 and Supplementary Table 2). No tissue-specific enrichment was observed for down-regulated expression.

### Gene Expression of ACE2-Interacting genes in Upper Respiratory Tissue for SARS-CoV-2 and Other Viruses

Using transcriptomic data related to acute respiratory illnesses for COVID-19 patients (*N* = 93), other viral (*N* = 41), or non-viral (*N* = 100) in the upper respiratory tract tissue (Mick et al., 2020), we found 35 of the 61 ACE2-interacting genes. Of the 35 genes, we found 19 genes that were reported as significant (Benjamini–Hochberg adjusted *p*-value < 0.05) for any of three comparisons, SARS-CoV-2 vs. no-virus, SARS-CoV-2 vs. other-virus, other-virus vs. no-virus (Supplementary Table 3). For the SARS-CoV-2 vs. no-virus, four of the ACE2-interacting genes were significant (*ACE2* logFC = 1.25; *CALM2* logFC = 0.36*; PRCP* logFC = *–*0.33*; RORB* logFC = 0.83). For differential gene expression between SARS-CoV-2 vs. other-virus and other-virus vs. no-virus, there were 16 and 11 genes, respectively, that were significant.

### Gene-Drug Interaction and Over-Represented Biological Functions

**FIGURE 2:**
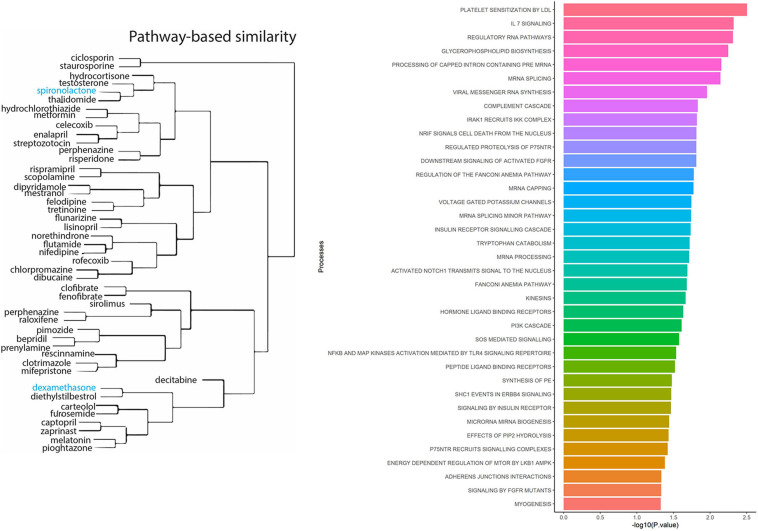
Drug-set enrichment analysis. LEFT: The similarity of drugs based on pathways identified. RIGHT: Biological Processes identified based on drugs that interact with genes from the ACE2-network.

To identify known drugs that interact with the ACE2-gene set, we investigated the drug-gene interaction database (DGIdb) (Griffith et al., 2013). Out of 61 genes, 29 had information about their drug-gene interaction in DGIdb, resulting in 238 unique drug-gene observations (Supplementary Table 4). Some of the notable drugs observed via this approach were spironolactone, dexamethasone, metformin, and hydrocortisone. To understand the role of these drugs in affecting biological processes, we performed drug-set enrichment analysis. DSEA (Napolitano et al., 2016) found gene-ontology mapping for 47 drugs and tested against REACTOME gene ontology database. Although the results did not survive Bonferroni correction, nominally significant enrichments were observed for platelet sensitization by low-density lipoprotein cholesterol (*p* = 0.003), IL-7 signaling (*p* = 0.004), glycerophospholipid biosynthesis (*p* = 0.005), and viral messenger RNA synthesis (*p* = 0.011) (Figure 2 and Supplementary Table 5).

### Over-Representation of Phenotypic Domains Within ACE2 Gene Network

**FIGURE 3:**
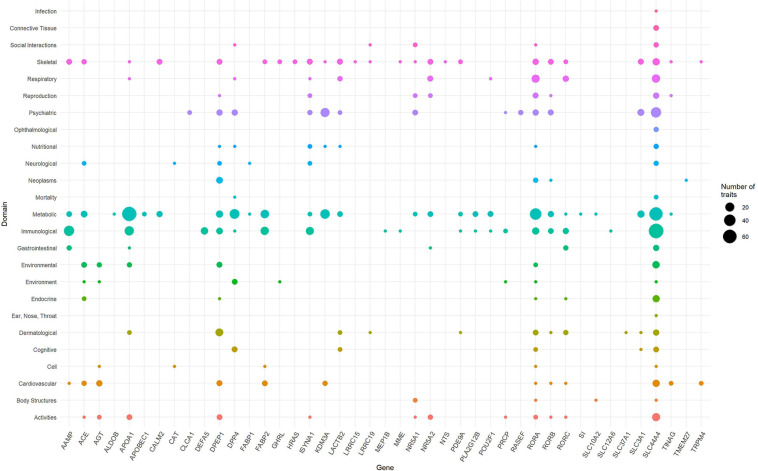
Domain distribution of PheWAS of ACE2-network genes. The ACE2 gene network associations are grouped based on domains (*y*-axis) and gene names (*x*-axis). The size of the data points reflects number of phenotypes and corresponding 43 genes surviving multiple testing correction.

To identify the phenotypes/traits associated with the ACE2-genes, we performed a phenome-wide association study (PheWAS). The GWASAtlas (Watanabe et al., 2019) contains 4756 traits categorized into 26 phenotype domains. We found 3983 phenotype associations for 51/61 genes, of which 43 genes with 476/3983 phenotypes were significant (*p* < 1e-5) (Supplementary Figures 2–52 and Supplementary Table 6). Most significant phenotypes were observed for *SLC44A4* with rheumatoid arthritis (*p* = 1.51 × 10^–105^) and white blood cell (*p* = 6.16 × 10^–105^). Significant phenotypes observed across several genes included body mass index, anthropometric traits, kidney function phenotypes—glomerular filtration rate, renin-angiotensin system, and lung capacity indices, i.e., FEV and FVC. The phenotype domains were tested for enrichment of significant traits vs. non-significant traits (Supplementary Table 7). Six domains were significant: “Immunological” (*p* = 7.62 × 10^–25^), “Respiratory” (*p* = 1.30 × 10^–8^), “Skeletal” (2.94 × 10^–8^), “Dermatological” (*p* = 7.91 × 10^–8^), “Environmental” (*p* = 2.21 × 10^–7^), and “Metabolic” (4.33 × 10^–4^) (Supplementary Table 8). *SLC44A4* had the highest number of associated traits across the significant domains (n_to__tal_ = 173) followed by *APOA1* had highest number of traits associations, mostly metabolic traits such as triglycerides, cholesterol, lipid measurements and blood cell measurements, i.e., platelet count and mean corpuscle volume (n_to__tal_ = 100; metabolic = 71 traits) (Figure 3). *SLC44A4*, *APOA1*, and *RORA* showed associations across all six enriched domains.

### Characterization of SNPs

**FIGURE 4:**
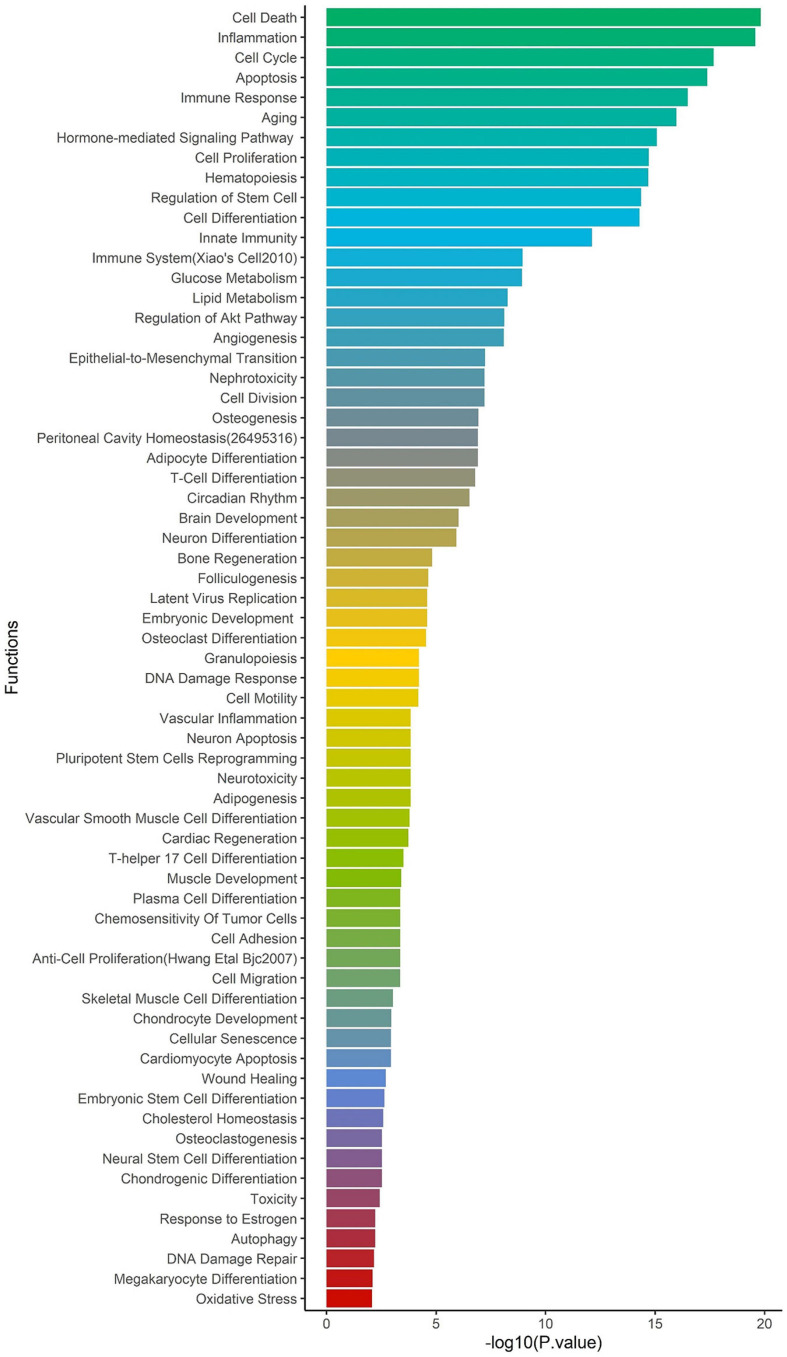
Enrichment of biological functions based on miRNA:SNP annotation. Using miRNAs annotation, over-represented biological processes are shown on *y*-axis and –log10 *p*-value on *x*-axis.

To identify the regulatory role of genetic variations for the ACE2-genes, we report their global allele frequency, functional consequence using pathogenic regulatory score, and disrupting miRNA sites. We extracted all 957,222 SNPs in the ACE2-network and annotated for allele frequency (Supplementary File 3), nearby genes and coordinates (Supplementary File 4), Combined Annotation Dependent Depletion (CADD) (Rentzsch et al., 2019) and DeepSEA (Zhou and Troyanskaya, 2015) scores. There were 98,529 SNPs with CADD score > 10, which corresponds to the top 10% pathogenic variants across the human genome (Supplementary File 5). To identify their regulatory consequences, variants were annotated with DeepSEA which provides functional probability of the SNPs in serving as gene expression, disease and chromatin regulating variants. There were 12,095 SNPs within the ACE2-gene network which had > 50% functional probability (DeepSEA functional score > 0.5) (Supplementary File 6). The miRNAs altered by the SNPs were analyzed for over-represented miRNA-family, biological functions, and diseases considering false discovery rate multiple testing correction (FDR *p* < 0.05). There were 4 miRNA clusters that were enriched, miR-302b, miR-181d (*p* = 0.0079), miR-17, and 106a (*p* = 0.00298). We found 65 biological functions that were significant and the top five significant biological processes were cell death (*p* = 1.5 × 10^–20^), inflammation (*p* = 2.57 × 10^–20^), cell cycle (*p* = 2.09 × 10^–18^), apoptosis (*p* = 4.15 × 10^–18^), and immune response (*p* = 3.17 × 10^–17^) (Figure 4). We observed a total of 152 significant diseases of which the most significant were diabetes mellitus type 2 (*p* = 1.55 × 10^–22^), hepatitis c virus infection (*p* = 5.56 × 10^–21^), atherosclerosis (*p* = 3.08 × 10^–19^), heart failure (*p* = 4.22 × 10^–19^), and Alzheimer’s disease (*p* = 1.35 × 10^–17^) (Supplementary Table 8).

### Neanderthal LA Introgression Within ACE2 Network SNPs

Due to the Neanderthal introgression observed in 3p21 locus as risk to COVID-19 (Zeberg and Pääbo, 2020), we compared mean probability of Neanderthal LA between the ACE2-network SNP set (mean = 0.032) and 1,000 randomly selected SNP sets with comparable genomic features (range of Neanderthal LA means = 0.027–0.036). The ACE2-network SNPs did not show evidence of Neanderthal LA introgression significantly different from those expected by chance (*p* = 0.663) (Supplementary Figure 55).

### Functional Annotation of Network SNPs Using the COVID-19 GWAS

We tested ACE2-network SNPs with respect to six COVID-19-related phenotypes (Freeze 3) released by the COVID-19 Host Genetics Initiative (Covid-19 Host Genetics Initiative (HGI), 2020). To identify independent variants, the variants were pruned for linkage disequilibrium (LD < 0.1 within 250 kb genomic size) and clumped for *p*-value < 0.01. Variants surviving multiple testing were annotated for eQTLs, and mQTLs. Three genes—*RORA*, *SLC12A6*, and *SLC6A19*—showed associations with multiple COVID-19 phenotypes (Supplementary Tables 9–14 and Supplementary Figures 56–61). *RORA* SNPs were associated with COVID-19 positive status (rs17303202, *p* = 2.35e-5), laboratory-confirmed positive COVID-19 status (rs4774377, *p* = 8.25e-5), hospitalized COVID-19 (rs17303202, *p* = 2.76e-05), and COVID-19 with very severe respiratory symptoms (rs341419, *p* = 8.13e-4). The SNPs (rs12912196) in *RORA* gene are also associated with gene expression (eQTL) of *RORA* gene (*p* = 3.9e-5) and mQTL (cg00930615, *p* = 7.84e-7) in *ANXA2*. *SLC12A6* associations were observed with respect to COVID-19 (rs145719616, *p* = 1.19e-4), hospitalized COVID-19 (rs192235418, *p* = 4.42e-4), COVID-19 with very severe respiratory (rs2705343, *p* = 1.86e-3), and. *SLC6A19* SNPs were associated with severe COVID-19 phenotype definitions, i.e., COVID-19 with very severe respiratory confirmed (rs76067074, *p* = 2.65e-3) and hospitalized COVID-19 (rs76067074, *p* = 2.52e-4). Furthermore, the GWAS of hospitalized COVID-19 identified 3p21.31 locus, wherein the genome-wide significant variant—rs13325613 (chr3:46298373bp; *p* = 1.17e-08) is a trans-QTL for genes *RORA* (*p* = 7.7e-7) and *RORC* (*p* = 1.3e-31).

**FIGURE 5:**
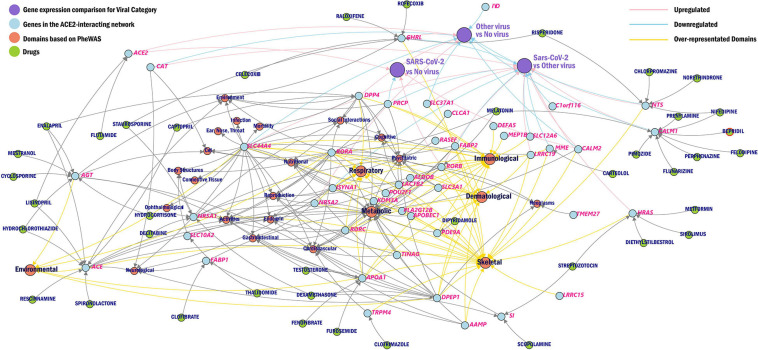
Network of ACE2 interacting genes. The overview of ACE2-network genes (blue) as drug targets (green), phenotype domains (red), and gene expression category (purple) wherein the genes are significant from the CZBioHub (see text for details). The pink edges represent upregulation and blue edges represent downregulation, and yellow edges connect genes to overrepresented phenotype domains.

We further created a unique comprehensive network by prioritizing genes from the ACE2-network using transcriptomic profile specific to SARS-CoV-2, over-represented domains for traits associated with ACE2-network genes, and the gene-drug targets that were overrepresented for biological functions (Figure 5).

## Discussion

*ACE2* is expressed in several tissues and plays a key role in host-entry of SARS-CoV-2 (Hoffmann et al., 2020). However, the genomic profile of *ACE2* is limited in explaining the vast symptomology observed for COVID-19. Understanding ACE2 associated molecular networks presents several functional insights between genetic targets based on gene expression, topology, and protein and signaling relationships (Huang et al., 2018). Due to the well-characterized role of ACE2 in SARS-CoV-2 infection, we generated novel information regarding the molecular and phenotypic characteristics of ACE gene network in the context of their potential involvement in COVID-19 susceptibility. Our PheWAS-based analysis showed that genetic variation within ACE2 gene network is associated with immunity (white blood cell, neutrophil count, lymphocyte count), respiratory (FVC, asthma, DVT), and metabolic traits (BMI, cholesterol, body measurements). This is in line with known epidemiology of COVID-19 and its comorbidities (Ejaz et al., 2020; Gardinassi et al., 2020).

The expression of ACE2-network genes was enriched for regulatory mechanisms related to small intestine, colon, kidney, and liver. It is hypothesized that furin, a serine protease present in lungs but also highly expressed in small intestine, and is involved in the cleavage of S-spike for attachment of the ACE2 receptor (Mönkemüller et al., 2020). Patients with kidney disease have higher risk for COVID-19 severe symptoms (Ajaimy and Melamed, 2020). Additionally, the inflammation and cytokine storm from COVID-19 is observed to damage kidney tissues (Gao et al., 2020). Lastly, modest increase in liver enzymes has been associated with COVID-19, and returning to baseline during the recovery phase (Pawlotsky, 2020).

Understanding the genes that interact with *ACE2* receptor has potential to understand drug-targets and molecular processes that might play a role in susceptibility and treatment response of COVID-19. The drug-gene interaction analysis within ACE2 network identified dexamethasone, reported to lower mortality in COVID-19 cases requiring mechanical ventilation (RECOVERY Collaborative Group et al., 2020). Drugs—spironolactone and hydrocortisone target the androgen system. Androgen signaling modulates ACE2 expression and elevated androgen levels have been associated with severe symptomology of COVID-19 (Samuel et al., 2020). Spironolactone is a diuretic and alleviates respiratory symptoms by reducing fluid from the lungs (Cadegiani et al., 2020). The use of spironolactone is currently being tested for acute respiratory distress syndrome in COVID-19 patients (Dumanlı et al., 2020). Hydrocortisone is currently under clinical trials for treating COVID-19 related hypoxia symptoms (Petersen et al., 2020). Among the other compounds identified, metformin, a known drug for treating diabetes, can also affect respiratory outcomes (Yen et al., 2020). A recent study reported protective effects of metformin in women with diabetes and obesity who were admitted with COVID-19 diagnosis (Bramante et al., 2020). Lastly, melatonin has been hypothesized to improve general immunity and lower oxidative stress generated from SARS-CoV-2 infection (Shneider et al., 2020).

The miRNA target sites altered by ACE2-network SNPs identified miR-302b and miR-181d as over-represented miRNA clusters. The downregulated expression of miR-302b has been observed to reduce survival rates in chronic obstructive pulmonary disease (COPD) patients (Keller et al., 2019). A meta-analysis showed that COPD diagnosis increased susceptibility to COVID-19 (Lippi and Henry, 2020). The miRNA-181 cluster has been associated with regulation of TNF-alpha (Zhu et al., 2017), T-cell aging (Ye et al., 2018) and emphysema (Osei et al., 2015). miRNA-17 and 106 belong to same miRNA family, miRNA-17 is upregulated in bronchoalveolar stem cells to lower SARS-CoV replication (Mallick et al., 2009). An *in silico* study of miRNA targets for SARS-CoV-2 genomic sequence found miRNA-17 as one of the targets with experimental evidence of its upregulation in H7N9 Influenza virus infection (Khan M.A.A.K. et al., 2020). The top over-represented diseases in miRNA-ACE2-network-SNPs were diabetes, hepatitis C viral infection, heart failure and Alzheimer’s disease. A greater number of diabetic individuals with COVID-19 have been reported to require hospitalization than non-diabetic individuals (Apicella et al., 2020). Furthermore, SARS-CoV-2 infection contributes in the development of ketosis in diabetic individuals resulting in longer length of hospitalization stay (Li et al., 2020). Triglyceride and glucose index was associated with severity of COVID-19 (Ren et al., 2020). While there are limited studies about hepatitis C in COVID-19 patients (Richardson et al., 2020), heart failure was reported by multiple studies as being associated with COVID-19 severity (Hanley et al., 2020; Yancy and Fonarow, 2020). Alzheimer’s disease is another condition associated with COVID-19 susceptibility (Chang et al., 2020), including *APOE4* carrier status with increased risk of severe COVID-19 (Kuo et al., 2020).

In contrast to specific enrichment of Neanderthal LA in a COVID-19 risk locus on chromosome 3 (Zeberg and Pääbo, 2020), there is no evidence of increased Neanderthal LA in the ACE2 network investigated here. This suggests that, although some loci conferring risk for COVID-19 severity, such as the one identified on chromosome 3, may have originated from Neanderthal admixture events, this mechanism did not shape the genetic architecture of the ACE2 network responsible for entry of SARS-CoV-2 into host cellular machinery.

Lastly, among ACE2-network-SNPs, potential COVID-19 risk alleles were observed in *RORA* gene with respect to multiple COVID-19 phenotypes. *RORA* protein product is involved in immune response, cancer and metabolism (Cook et al., 2015). *RORA* plays a role in the activation of T helper cells during lung inflammation by regulating tumor necrosis factor and interleukins (Nejati Moharrami et al., 2018; Haim-Vilmovsky et al., 2019), and was upregulated in cardiomyocytes infected with SARS-CoV-2 (Hachim et al., 2020). The hypothesis-free approach of genome-wide association of hospitalized COVID-19 vs. the population highlighted *SLC6A20* with genome-wide significance on chromosome 3 locus. The SLC12 (*SLC12A6*) class is responsible for transport of inorganic ions such as sodium and chloride while the SLC6 class (*SLC6A19*, identified via network approach and SLC6A20, identified via genome-wide approach) are responsible for transport of amino acids such as glutamate and glycine which are important for neurotransmitter activity (Lin et al., 2015). *SLC6A19* (among other SLC-class genes) serves similar function to *SLC6A20*, both are expressed in the intestinal tissue and contingent upon ACE2 expression (Vuille-Dit-Bille et al., 2020). Multiple studies report more than 10% of the COVID-19 confirmed patients exhibit gastrointestinal symptoms (Jin et al., 2020; Khan M. et al., 2020; Lian et al., 2020).

Although we provided a wide range of information highlighting the molecular and phenotypic characteristics of *ACE2* gene network and their putative implications with COVID-19 risk, the findings reported have to be considered exploratory. We used appropriate computational methods and statistical approaches to generate reliable evidence useful to open new directions in COVID-19 research. We also highlighted when the results reported did not survive stringent multiple testing correction. This limitation is particularly relevant with respect to the ACE2 network genetic associations. Due to the limited statistical power of the genome-wide data available for the Freeze 3 data from the COVID-19 Host Genetics Initiative, none of the risk alleles identified as functionally relevant survive genome-wide testing correction. Future work from the HGI will potentially lead to more risk loci being identified. Further analyses will be needed to validate our current findings.

## Conclusion

*ACE2* is one of the few molecular targets recognized to play a key role in the COVID-19 pathogenesis. We conducted a comprehensive analysis leveraging multiple resources (e.g., drug-gene interactions, tissue-specific transcriptomic profile, and phenome-wide and genome-wide datasets) to expand our understanding of the genomic characteristics of the host *ACE2* gene network. Overall, our findings incorporate multi-tiered epigenomic, transcriptomic, and genomics of the known ACE2-network which highlight the potential mechanisms linking *ACE2* systems biology to COVID-19 susceptibility and its possible comorbidities.

## Methods

### Gene Network Collection

Information regarding ACE2 gene network was mined from GeneMANIA (Franz et al., 2018), Stringdb (Szklarczyk et al., 2017), Agile Protein Interactomes Database (APID) (Prieto and De Las Rivas, 2006), GeneNetwork (Deelen et al., 2019), Biogrid (Oughtred et al., 2019), and FunctionalNet (Lee et al., 2011) for Homo sapiens organism, last searched on June 27, 2020. Since all resources use different algorithms, using *ACE2* as query gene, immediate genes connections that were available in each databank were retrieved with their default settings. Removing overlapping genes across the six databases, resulted in 61 unique genes (60 genes plus *ACE2*) (Supplementary Figure 1 and Supplementary Table 1). Specifically, GeneMANIA uses automatically selected weighting method for 20 max resultant genes with 10 max resultant attributes. In Stringdb, the ACE2 gene query with medium confidence interaction score, and including all active interaction source except text mining. For APID, single query of ACE2 generated a network of 11 interacting genes. In GeneNetwork, we used ACE2 as query genes and selected co-regulated genes with evidence of *p* < 2e-12. In Biogrid, we used ACE2 in Homo Sapiens as query, and used the network information. In FunctionalNet, the ACE2 was searched using its ENTREZ id (59272) and selected the genelist that interact with ACE2. We focused on the genes that interact immediately with ACE2 because including more genes that interact through intermediate genes will result in large volume data and difficult to interpret. Several genes were identified by multiple sources listed in Supplementary Table 1, and a total of 61 genes including ACE2 were investigated for their characteristics. The genomic coordinates for the genes were annotated using biomart (Durinck et al., 2009), ensemble GRCh37/hg19. The analysis and visualization were performed in R 3.6.

### Tissue-Specific Transcriptomic Regulation

The tissue specificity was tested for 60 ACE2-interacting genes in FUMA (Watanabe et al., 2017). The input genes were tested for pre-calculated tissue-specific differentially expressed genes from the GTEx v8 (Aguet et al., 2019). We also considered the t-statistic sign for up and down-regulated genes against protein coding genes as background. Enrichments were performed using hypergeometric tests and significant enrichments were defined according to Bonferroni corrected *p*-value < 0.05.

### Gene Expression of ACE2-Interacting Genes in Upper Respiratory Tissue for SARS-CoV-2 and Other Viruses

To understand which genes from the ACE2-interacting genes are differentially expressed in SARS-CoV-2 and other viruses, we extracted these genes from transcriptomic study of acute respiratory illnesses for COVID-19 patients (*N* = 93), other viral (*N* = 41) or non-viral (*N* = 100) in the upper respiratory tract tissue (Mick et al., 2020). Their data was extracted from https://github.com/czbiohub/covid19-transcriptomics-pathogenesis-diagnostics-results. We used ENSEMBL identifiers of 61 ACE2 interacting genes and were able to extract 35/61 genes. The study performed three gene expression comparisons, SARS-CoV-2 vs. no-virus, SARS-CoV-2 vs. other-virus, other-virus vs. no-virus, and genes with Benjamin-Hochberg adjusted *p*-value < 0.05 were assigned as significant to the respective comparison.

### Phenome-Wide Analysis of ACE2 Gene Network

A phenome-wide association study (PheWAS) was performed for 51 of 61 genes that were present in GWASAtlas (Watanabe et al., 2019) using all traits available per gene. Statistical significance was determined by applying a Bonferroni multiple-testing correction accounting for the number of GWAS traits (4,765 traits) available in the GWASAtlas (*p* < 1.05 × 10^–5^). Each trait was grouped into a domain (Supplementary Table 5) which was tested for enrichment using one-sided Fisher’s exact test for high proportion of significant traits vs. all others tested. A significant domain enrichment was defined considering a Bonferroni-corrected threshold accounting for the number of domains tested (*p*-value < 0.0019; 0.05/26).

### Gene-Drug Interactions and Biological Functions

Information on drugs that interact with ACE2 network genes were extracted from The Drug-Gene Interaction database (DGIdb) (Griffith et al., 2013) followed by drug-set enrichment for over represented biological functions using DSEA (Drug-Set Enrichment Analysis) (Napolitano et al., 2016).

### Characterization of SNPs

Single nucleotide polymorphism (SNPs) were extracted based on the genomic coordinates of the genes (± 10 kb) for GrCh37; dbSNP153 from the UCSC browser (Haeussler et al., 2019) using bigbed utilities (Karolchik et al., 2004), and referred to as “ACE2-network SNPs.” ACE2-network SNPs were annotated for global allele frequency, Combined Annotation-Dependent Depletion (CADD) score (Rentzsch et al., 2019), deep learning based algorithm framework (DeepSEA) (Zhou and Troyanskaya, 2015), and target miRNAs using SNPnexus (Dayem Ullah et al., 2018). DeepSEA is a deep learning-based algorithmic framework for predicting the chromatin effects of sequence alterations with single nucleotide sensitivity (Zhou and Troyanskaya, 2015). The identified miRNAs were tested for over-represented miRNA clusters, functions, and diseases using TAM 2.0 (Li et al., 2018).

### Neanderthal Introgression

Motivated by evidence of a chromosome 3 COVID-19 risk locus enriched of Neanderthal local ancestry (LA) (Zeberg and Pääbo, 2020), we compared the distribution of probability of Neanderthal LA in our COVID-19 ACE2-network SNP set and 1,000 randomly sampled SNP sets comprised on SNPs across the genome with comparable genomic features. ACE2-network SNPs were mapped using previously defined Neanderthal LA data (Sankararaman et al., 2014; Durvasula and Sankararaman, 2019). A total of 6,822 LD-independent pairwise SNPs (*r^2^* = 0.1 and *p* = 0.1 in 250 kb window size) were used as standard input for SNPsnap (Pers et al., 2015). In SNPsnap, 1,249/6,822 independent ACE2 network SNPs could be matched based on the following genomic features relative to the input SNP list: minor allele frequency within 2%, gene density within 50%, nearest gene within 50%, and number of linkage disequilibrium groups within 50%. SNPsnap was instructed to exclude the ACE2-network SNP list from the pool of eligible feature-matched SNPs. Non-parametric Wilcoxon rank sum tests were used to compare the Neanderthal LA of our ACE2 network SNP list to that of all 1,000 random SNP sets and multiple testing correction was applied to adjust for a false discovery rate of 5%.

### Association Statistics of ACE2 Network SNPs From the COVID-19 Host Genetics Initiative (HGI)

The ACE2-network SNPs were extracted from association statistics released by the COVID-19 HGI (COVID-19 Host Genetics Initiative [HGI], 2020) for six phenotypes describing COVID-19 susceptibility. These phenotypes were A2_V2 [very severe respiratory confirmed COVID-19 cases (*N* = 536) vs. population (*N* = 329391)], B1_V2 [hospitalized COVID-19 cases (*N* = 928) vs. not hospitalized COVID-19 cases (*N* = 2028)], B2_V2 [hospitalized COVID-19 cases (*N* = 3199) vs. population (*N* = 897488)], C1_V2 [COVID-19 cases (*N* = 3523) vs. lab/self-reported negative (*N* = 36634)], C2_V2 [COVID-19 cases (*N* = 6696) vs. population (*N* = 1073072)], and D1_V2 [predicted COVID-19 cases from self-reported symptoms (*N* = 1865) vs. predicted or self-reported non-COVID-19 cases (*N* = 29174)]. The SNPs of the ACE2 network were extracted and pruned for LD and *p*-value using plink 1.9. The multiple testing correction was applied using Bonferroni *p*-value < 0.05. These significant SNPs were annotated further for pathogenicity using Combined Annotation Dependent Depletion (CADD) score and their role as quantitative trait loci (QTL) for gene expression using GTEx, and methylation using QTLbase (Zheng et al., 2020). The trans-eQTL relationship of GWAS-reported locus-3p21.31 were identified from eQTLgen (Võsa et al., 2018).

## Data Availability Statement

The raw data supporting the conclusions of this article will be made available by the authors, without undue reservation, to any qualified researcher. The data presented is available in Supplementary Files and also on https://gpwhiz.github.io/ACE2Netlas/.

## Ethics Statement

Ethical review and approval was not required for the study on human participants in accordance with the local legislation and institutional requirements. The patients/participants provided their written informed consent to participate in their original studies reported.

## Author Contributions

GP conceptualized the study design, analyzed, and drafted the manuscript. FW contributed to analysis and manuscript writing. AG, DK, FD, and RP contributed to result interpretation, manuscript drafting and revision. RP supervised the study and finalized the manuscript. All authors contributed to the article and approved the submitted version.

## Conflict of Interest

The authors declare that the research was conducted in the absence of any commercial or financial relationships that could be construed as a potential conflict of interest.

## Publisher’s Note

All claims expressed in this article are solely those of the authors and do not necessarily represent those of their affiliated organizations, or those of the publisher, the editors and the reviewers. Any product that may be evaluated in this article, or claim that may be made by its manufacturer, is not guaranteed or endorsed by the publisher.

## References

[B1] Aguet F. Barbeira A. N. Bonazzola R. Brown A. Castel S. E. Jo B. (2019). The GTEx Consortium atlas of genetic regulatory effects across human tissues. *bioRxiv* [Preprint]. 10.1101/787903

[B2] Ajaimy M. Melamed M. L. (2020). COVID-19 in Patients with Kidney Disease. *Clin. J. Am. Soc. Nephrol.* 15 1087–1089. 10.2215/CJN.09730620 32636199 PMC7409763

[B3] Apicella M. Campopiano M. C. Mantuano M. Mazoni L. Coppelli A. Del Prato S. (2020). COVID-19 in people with diabetes: understanding the reasons for worse outcomes. *Lancet Diabetes Endocrinol.* 8 782–792. 10.1016/S2213-8587(20)30238-2 32687793 PMC7367664

[B4] Bramante C. Ingraham N. Murray T. Marmor S. Hoversten S. Gronski J. (2020). Observational Study of Metformin and Risk of Mortality in Patients Hospitalized with Covid-19. *medRxiv* [Preprint]. 10.1101/2020.06.19.20135095 32607520 PMC7325185

[B5] Cadegiani F. A. Goren A. Wambier C. G. (2020). Spironolactone may provide protection from SARS-CoV-2: targeting androgens, angiotensin converting enzyme 2 (ACE2), and renin-angiotensin-aldosterone system (RAAS). *Med. Hypotheses* 143:110112. 10.1016/j.mehy.2020.110112 32721806 PMC7363620

[B6] Chang T. S. Ding Y. Freund M. K. Johnson R. Schwarz T. Yabu J. M. (2020). Prior diagnoses and medications as risk factors for COVID-19 in a Los Angeles Health System. *medRxiv* [Preprint]. 10.1101/2020.07.03.20145581 32637977 PMC7340203

[B7] Cook D. N. Kang H. S. Jetten A. M. (2015). Retinoic Acid-Related Orphan Receptors (RORs): regulatory Functions in Immunity, Development, Circadian Rhythm, and Metabolism. *Nucl. Receptor Res.* 2:101185. 10.11131/2015/101185 26878025 PMC4750502

[B8] Covid-19 Host Genetics Initiative (HGI). (2020). The COVID-19 Host Genetics Initiative, a global initiative to elucidate the role of host genetic factors in susceptibility and severity of the SARS-CoV-2 virus pandemic. *Eur. J. Hum. Genet.* 28 715–718. 10.1038/s41431-020-0636-6 32404885 PMC7220587

[B9] Dayem Ullah A. Z. Oscanoa J. Wang J. Nagano A. Lemoine N. R. Chelala C. (2018). SNPnexus: assessing the functional relevance of genetic variation to facilitate the promise of precision medicine. *Nucleic Acids Res.* 46 W109–W113. 10.1093/nar/gky399 29757393 PMC6030955

[B10] Deelen P. van Dam S. Herkert J. C. Karjalainen J. M. Brugge H. Abbott K. M. (2019). Improving the diagnostic yield of exome- sequencing by predicting gene-phenotype associations using large-scale gene expression analysis. *Nat. Commun.* 10:2837. 10.1038/s41467-019-10649-4 31253775 PMC6599066

[B11] Dumanlı G. Y. Dilken O. Ürkmez S. (2020). Use of Spironolactone in SARS-CoV-2 ARDS Patients. *Turk. J. Anaesthesiol. Reanim.* 48 254–255. 10.5152/TJAR.2020.569 32551456 PMC7279869

[B12] Durinck S. Spellman P. T. Birney E. Huber W. (2009). Mapping identifiers for the integration of genomic datasets with the R/Bioconductor package biomaRt. *Nat. Protoc.* 4 1184–1191. 10.1038/nprot.2009.97 19617889 PMC3159387

[B13] Durvasula A. Sankararaman S. (2019). A statistical model for reference-free inference of archaic local ancestry. *PLoS Genet.* 15:e1008175. 10.1371/journal.pgen.1008175 31136573 PMC6555542

[B14] Ejaz H. Alsrhani A. Zafar A. Javed H. Junaid K. Abdalla A. E. (2020). COVID-19 and comorbidities: deleterious impact on infected patients. *J. Infect. Public Health* 13 1833–1839. 10.1016/j.jiph.2020.07.014 32788073 PMC7402107

[B15] Ellinghaus D. Degenhardt F. Bujanda L. Buti M. Albillos A. Invernizzi P. (2020). Genomewide Association Study of Severe Covid-19 with Respiratory Failure. *N. Engl. J. Med.* 383 1522–1534. 10.1056/NEJMoa2020283 32558485 PMC7315890

[B16] Franz M. Rodriguez H. Lopes C. Zuberi K. Montojo J. Bader G. D. (2018). GeneMANIA update 2018. *Nucleic Acids Res.* 46 W60–W64. 10.1093/nar/gky311 29912392 PMC6030815

[B17] Gao M. Wang Q. Wei J. Zhu Z. Li H. (2020). Severe Coronavirus disease 2019 pneumonia patients showed signs of aggravated renal impairment. *J. Clin. Lab. Anal.* 34:e23535. 10.1002/jcla.23535 32840917 PMC7461016

[B18] Gardinassi L. G. Souza C. O. S. Sales-Campos H. Fonseca S. G. (2020). Immune and Metabolic Signatures of COVID-19 Revealed by Transcriptomics Data Reuse. *Front. Immunol.* 11:1636. 10.3389/fimmu.2020.01636 32670298 PMC7332781

[B19] Griffith M. Griffith O. L. Coffman A. C. Weible J. V. McMichael J. F. Spies N. C. (2013). DGIdb: mining the druggable genome. *Nat. Methods* 10 1209–1210. 10.1038/nmeth.2689 24122041 PMC3851581

[B20] Hachim M. Y. Al Heialy S. Senok A. Hamid Q. Alsheikh-Ali A. (2020). Molecular Basis of Cardiac and Vascular Injuries Associated With COVID-19. *Front. Cardiovasc. Med.* 7:582399. 10.3389/fcvm.2020.582399 33240937 PMC7669624

[B21] Haeussler M. Zweig A. S. Tyner C. Speir M. L. Rosenbloom K. R. Raney B. J. (2019). The UCSC Genome Browser database: 2019 update. *Nucleic Acids Res.* 47 D853–D858. 10.1093/nar/gky1095 30407534 PMC6323953

[B22] Haim-Vilmovsky L. Walker J. A. Henriksson J. Miao Z. Natan E. Kar G. (2019). *Rora* regulates activated T helper cells during inflammation. *bioRxiv* [Preprint]. 10.1101/709998

[B23] Hamming I. Cooper M. E. Haagmans B. L. Hooper N. M. Korstanje R. Osterhaus A. D. M. E. (2007). The emerging role of ACE2 in physiology and disease. *J. Pathol.* 212 1–11. 10.1002/path.2162 17464936 PMC7167724

[B24] Hanley B. Naresh K. N. Roufosse C. Nicholson A. G. Weir J. Cooke G. S. (2020). Histopathological findings and viral tropism in UK patients with severe fatal COVID-19: a post-mortem study. *Lancet Microbe* 1 E245–E253. 10.1016/S2666-5247(20)30115-4 32844161 PMC7440861

[B25] Hoffmann M. Kleine-Weber H. Schroeder S. Krüger N. Herrler T. Erichsen S. (2020). SARS-CoV-2 Cell Entry Depends on ACE2 and TMPRSS2 and Is Blocked by a Clinically Proven Protease Inhibitor. *Cell* 181 271–280.e8. 10.1016/j.cell.2020.02.052 32142651 PMC7102627

[B26] Huang J. K. Carlin D. E. Yu M. K. Zhang W. Kreisberg J. F. Tamayo P. (2018). Systematic evaluation of molecular networks for discovery of disease genes. *Cell Syst.* 6 484–495.e5. 10.1016/j.cels.2018.03.001 29605183 PMC5920724

[B27] Jin X. Lian J.-S. Hu J.-H. Gao J. Zheng L. Zhang Y.-M. (2020). Epidemiological, clinical and virological characteristics of 74 cases of coronavirus-infected disease 2019 (COVID-19) with gastrointestinal symptoms. *Gut* 69 1002–1009. 10.1136/gutjnl-2020-320926 32213556 PMC7133387

[B28] Karolchik D. Hinrichs A. S. Furey T. S. Roskin K. M. Sugnet C. W. Haussler D. (2004). The UCSC Table Browser data retrieval tool. *Nucleic Acids Res.* 32 D493–D496. 10.1093/nar/gkh103 14681465 PMC308837

[B29] Keller A. Ludwig N. Fehlmann T. Kahraman M. Backes C. Kern F. (2019). Low miR-150-5p and miR-320b Expression Predicts Reduced Survival of COPD Patients. *Cells* 8:1162. 10.3390/cells8101162 31569706 PMC6848926

[B30] Khan M. A. A. K. Sany M. R. U. Islam M. S. Mehebub M. S. Islam A. B. M. M. K. (2020). Epigenetic regulator miRNA pattern differences among SARS-CoV, SARS-CoV-2 and SARS-CoV-2 world-wide isolates delineated the mystery behind the epic pathogenicity and distinct clinical characteristics of pandemic COVID-19. *bioRxiv* [Preprint]. 10.1101/2020.05.06.081026 PMC7381279 32765592

[B31] Khan M. Khan H. Khan S. Nawaz M. (2020). Epidemiological and clinical characteristics of coronavirus disease (COVID-19) cases at a screening clinic during the early outbreak period: a single-centre study. *J. Med. Microbiol.* 69 1114–1123. 10.1099/jmm.0.001231 32783802 PMC7642977

[B32] Kuo C.-L. Pilling L. C. Atkins J. L. Masoli J. A. H. Delgado J. Kuchel G. A. (2020). APOE e4 genotype predicts severe COVID-19 in the UK Biobank community cohort. *J. Gerontol. A Biol. Sci. Med. Sci.* 75 2231–2232. 10.1093/gerona/glaa131 32451547 PMC7314139

[B33] Lee I. Blom U. M. Wang P. I. Shim J. E. Marcotte E. M. (2011). Prioritizing candidate disease genes by network-based boosting of genome-wide association data. *Genome Res.* 21 1109–1121. 10.1101/gr.118992.110 21536720 PMC3129253

[B34] Li J. Han X. Wan Y. Zhang S. Zhao Y. Fan R. (2018). TAM 2.0: tool for MicroRNA set analysis. *Nucleic Acids Res.* 46 W180–W185. 10.1093/nar/gky509 29878154 PMC6031048

[B35] Li J. Wang X. Chen J. Zuo X. Zhang H. Deng A. (2020). COVID-19 infection may cause ketosis and ketoacidosis. *Diabetes Obes. Metab.* 22 1935–1941. 10.1111/dom.14057 32314455 PMC7264681

[B36] Lian J. Jin X. Hao S. Jia H. Cai H. Zhang X. (2020). Epidemiological, clinical, and virological characteristics of 465 hospitalized cases of coronavirus disease 2019 (COVID-19) from Zhejiang province in China. *Influenza Other Respir. Viruses* 14 564–574. 10.1111/irv.12758 32397011 PMC7273099

[B37] Lin L. Yee S. W. Kim R. B. Giacomini K. M. (2015). SLC transporters as therapeutic targets: emerging opportunities. *Nat. Rev. Drug Discov.* 14 543–560. 10.1038/nrd4626 26111766 PMC4698371

[B38] Lippi G. Henry B. M. (2020). Chronic obstructive pulmonary disease is associated with severe coronavirus disease 2019 (COVID-19). *Respir. Med.* 167:105941. 10.1016/j.rmed.2020.105941 32421537 PMC7154502

[B39] Mallick B. Ghosh Z. Chakrabarti J. (2009). MicroRNome analysis unravels the molecular basis of SARS infection in bronchoalveolar stem cells. *PLoS One* 4:e7837. 10.1371/journal.pone.0007837 19915717 PMC2773932

[B40] Mercurio I. Tragni V. Busto F. De Grassi A. Pierri C. L. (2021). Protein structure analysis of the interactions between SARS-CoV-2 spike protein and the human ACE2 receptor: from conformational changes to novel neutralizing antibodies. *Cell Mol. Life Sci.* 78 1501–1522. 10.1007/s00018-020-03580-1 32623480 PMC7334636

[B41] Mick E. Kamm J. Pisco A. O. Ratnasiri K. Babik J. M. Castañeda G. (2020). Upper airway gene expression reveals suppressed immune responses to SARS-CoV-2 compared with other respiratory viruses. *Nat. Commun.* 11:5854. 10.1038/s41467-020-19587-y 33203890 PMC7673985

[B42] Mönkemüller K. Fry L. Rickes S. (2020). COVID-19, coronavirus, SARS-CoV-2 and the small bowel. *Rev. Esp. Enferm. Dig.* 112 383–388. 10.17235/reed.2020.7137/2020 32343593

[B43] Napolitano F. Sirci F. Carrella D. di Bernardo D. (2016). Drug-set enrichment analysis: a novel tool to investigate drug mode of action. *Bioinformatics* 32 235–241. 10.1093/bioinformatics/btv536 26415724 PMC4795590

[B44] Nejati Moharrami N. Bjørkøy Tande E. Ryan L. Espevik T. Boyartchuk V. (2018). RORα controls inflammatory state of human macrophages. *PLoS One* 13:e0207374. 10.1371/journal.pone.0207374 30485323 PMC6261595

[B45] Osei E. T. Florez-Sampedro L. Timens W. Postma D. S. Heijink I. H. Brandsma C.-A. (2015). Unravelling the complexity of COPD by microRNAs: it’s a small world after all. *Eur. Respir. J.* 46 807–818. 10.1183/13993003.02139-2014 26250493

[B46] Oughtred R. Stark C. Breitkreutz B.-J. Rust J. Boucher L. Chang C. (2019). The BioGRID interaction database: 2019 update. *Nucleic Acids Res.* 47 D529–D541. 10.1093/nar/gky1079 30476227 PMC6324058

[B47] Pairo-Castineira E. Clohisey S. Klaric L. Bretherick A. Rawlik K. Parkinson N. (2020). Genetic mechanisms of critical illness in Covid-19. *medRxiv* [Preprint]. 10.1101/2020.09.24.20200048 33307546

[B48] Pawlotsky J.-M. (2020). COVID-19 and the liver-related deaths to come. *Nat. Rev. Gastroenterol. Hepatol.* 17 523–525. 10.1038/s41575-020-0328-2 32528138 PMC7288259

[B49] Pers T. H. Timshel P. Hirschhorn J. N. (2015). SNPsnap: a Web-based tool for identification and annotation of matched SNPs. *Bioinformatics* 31 418–420. 10.1093/bioinformatics/btu655 25316677 PMC4308663

[B50] Petersen M. W. Meyhoff T. S. Helleberg M. Kjær M.-B. N. Granholm A. Hjortsø C. J. S. (2020). Low-dose hydrocortisone in patients with COVID-19 and severe hypoxia (COVID STEROID) trial-Protocol and statistical analysis plan. *Acta Anaesthesiol. Scand.* 64 1365–1375. 10.1111/aas.13673 32779728 PMC7404666

[B51] Prieto C. De Las Rivas J. (2006). APID: agile protein interaction dataanalyzer. *Nucleic Acids Res.* 34 W298–W302. 10.1093/nar/gkl128 16845013 PMC1538863

[B52] RECOVERY Collaborative Group P. Lim W. S. Emberson J. R. Mafham M. Bell J. L. (2020). Dexamethasone in Hospitalized Patients with Covid-19 - Preliminary Report. *N. Engl. J. Med.* 384 693–704. 10.1056/NEJMoa2021436 32678530 PMC7383595

[B53] Ren H. Yang Y. Wang F. Yan Y. Shi X. Dong K. (2020). Association of the insulin resistance marker TyG index with the severity and mortality of COVID-19. *Cardiovasc. Diabetol.* 19:58. 10.1186/s12933-020-01035-2 32393351 PMC7213552

[B54] Rentzsch P. Witten D. Cooper G. M. Shendure J. Kircher M. (2019). CADD: predicting the deleteriousness of variants throughout the human genome. *Nucleic Acids Res.* 47 D886–D894. 10.1093/nar/gky1016 30371827 PMC6323892

[B55] Richardson S. Hirsch J. S. Narasimhan M. Crawford J. M. McGinn T. Davidson K. W. (2020). Presenting Characteristics, Comorbidities, and Outcomes Among 5700 Patients Hospitalized With COVID-19 in the New York City Area. *JAMA* 323 2052–2059. 10.1001/jama.2020.6775 32320003 PMC7177629

[B56] Samuel R. M. Majd H. Richter M. N. Ghazizadeh Z. Zekavat S. M. Navickas A. (2020). Androgen Signaling Regulates SARS-CoV-2 Receptor Levels and Is Associated with Severe COVID-19 Symptoms in Men. *Cell Stem Cell* 27 876–889.e12. 10.1016/j.stem.2020.11.009 33232663 PMC7670929

[B57] Sankararaman S. Mallick S. Dannemann M. Prüfer K. Kelso J. Pääbo S. (2014). The genomic landscape of Neanderthal ancestry in present-day humans. *Nature* 507 354–357. 10.1038/nature12961 24476815 PMC4072735

[B58] Shelton J. F. Shastri A. J. Ye C. Weldon C. H. Filshtein-Somnez T. Coker D. (2020). Trans-ethnic analysis reveals genetic and non-genetic associations with COVID-19 susceptibility and severity. *medRxiv* [Preprint]. 10.1101/2020.09.04.20188318 33888907

[B59] Shneider A. Kudriavtsev A. Vakhrusheva A. (2020). Can melatonin reduce the severity of COVID-19 pandemic? *Int. Rev. Immunol.* 39 153–162. 10.1080/08830185.2020.1756284 32347747

[B60] Szklarczyk D. Morris J. H. Cook H. Kuhn M. Wyder S. Simonovic M. (2017). The STRING database in 2017: quality-controlled protein-protein association networks, made broadly accessible. *Nucleic Acids Res.* 45 D362–D368. 10.1093/nar/gkw937 27924014 PMC5210637

[B61] Võsa U. Claringbould A. Westra H. J. Bonder M. J. (2018). Unraveling the polygenic architecture of complex traits using blood eQTL metaanalysis. *bioRxiv* [Preprint]. 10.1101/447367

[B62] Vuille-Dit-Bille R. N. Liechty K. W. Verrey F. Guglielmetti L. C. (2020). SARS-CoV-2 receptor ACE2 gene expression in small intestine correlates with age. *Amino Acids* 52 1063–1065. 10.1007/s00726-020-02870-z 32627059 PMC7335412

[B63] Walls A. C. Park Y.-J. Tortorici M. A. Wall A. McGuire A. T. Veesler D. (2020). Structure, Function, and Antigenicity of the SARS-CoV-2 Spike Glycoprotein. *Cell* 181 281–292.e6. 10.1016/j.cell.2020.02.058 32155444 PMC7102599

[B64] Wang D. Hu B. Hu C. Zhu F. Liu X. Zhang J. (2020). Clinical Characteristics of 138 Hospitalized Patients With 2019 Novel Coronavirus-Infected Pneumonia in Wuhan, China. *JAMA* 323 1061–1069. 10.1001/jama.2020.1585 32031570 PMC7042881

[B65] Watanabe K. Stringer S. Frei O. Umićević Mirkov M. de Leeuw C. Polderman T. J. C. (2019). A global overview of pleiotropy and genetic architecture in complex traits. *Nat. Genet.* 51 1339–1348. 10.1038/s41588-019-0481-0 31427789

[B66] Watanabe K. Taskesen E. van Bochoven A. Posthuma D. (2017). Functional mapping and annotation of genetic associations with FUMA. *Nat. Commun.* 8:1826. 10.1038/s41467-017-01261-5 29184056 PMC5705698

[B67] Wu F. Zhao S. Yu B. Chen Y.-M. Wang W. Song Z.-G. (2020). A new coronavirus associated with human respiratory disease in China. *Nature* 579 265–269. 10.1038/s41586-020-2008-3 32015508 PMC7094943

[B68] Yancy C. W. Fonarow G. C. (2020). Coronavirus Disease 2019 (COVID-19) and the Heart-Is Heart Failure the Next Chapter? *JAMA Cardiol.* 5 1216–1217. 10.1001/jamacardio.2020.3575 32730614

[B69] Ye Z. Li G. Kim C. Hu B. Jadhav R. R. Weyand C. M. (2018). Regulation of miR-181a expression in T cell aging. *Nat. Commun.* 9:3060. 10.1038/s41467-018-05552-3 30076309 PMC6076328

[B70] Yen F.-S. Wei J. C.-C. Yang Y.-C. Hsu C.-C. Hwu C.-M. (2020). Respiratory outcomes of metformin use in patients with type 2 diabetes and chronic obstructive pulmonary disease. *Sci. Rep.* 10:10298. 10.1038/s41598-020-67338-2 32581289 PMC7314747

[B71] Zeberg H. Pääbo S. (2020). The major genetic risk factor for severe COVID-19 is inherited from Neanderthals. *Nature* 587 610–612. 10.1038/s41586-020-2818-3 32998156

[B72] Zheng Z. Huang D. Wang J. Zhao K. Zhou Y. Guo Z. (2020). QTLbase: an integrative resource for quantitative trait loci across multiple human molecular phenotypes. *Nucleic Acids Res.* 48 D983–D991. 10.1093/nar/gkz888 31598699 PMC6943073

[B73] Zhou J. Troyanskaya O. G. (2015). Predicting effects of noncoding variants with deep learning-based sequence model. *Nat. Methods* 12 931–934. 10.1038/nmeth.3547 26301843 PMC4768299

[B74] Zhou P. Yang X.-L. Wang X.-G. Hu B. Zhang L. Zhang W. (2020). A pneumonia outbreak associated with a new coronavirus of probable bat origin. *Nature* 579 270–273. 10.1038/s41586-020-2012-7 32015507 PMC7095418

[B75] Zhu J. Wang F.-L. Wang H.-B. Dong N. Zhu X.-M. Wu Y. (2017). TNF-α mRNA is negatively regulated by microRNA-181a-5p in maturation of dendritic cells induced by high mobility group box-1 protein. *Sci. Rep.* 7:12239. 10.1038/s41598-017-12492-3 28947753 PMC5612954

