# Supplementary material for: ACE2 Netlas: In silico Functional Characterization and Drug-Gene Interactions of ACE2 Gene Network to Understand Its Potential Involvement in COVID-19 Susceptibility
Source: Front Genet. 2021 Aug 27;12:698033. doi: 10.3389/fgene.2021.698033 (PMC8429844; doi:10.3389/fgene.2021.698033)
Supplement: Supplementary file 2 [file Data_Sheet_2.docx]

Supplementary file 1

**ACE2 Netlas: Functional characterization and drug-gene interactions of ACE2 gene network and its potential involvement in COVID-19 susceptibility**

Contents

[Study Overview 2](#_Toc52268796)

[PheWAS of genes in the ACE2 network 3](#_Toc52268797)

[miRNA enrichment 28](#_Toc52268798)

[Neanderthal local ancestry assessment for the SNPs in the ACE2 gene network 30](#_Toc52268799)

[ACE2 network SNPs in six phenotypes of COVID-19 31](#_Toc52268800)

# Study Overview


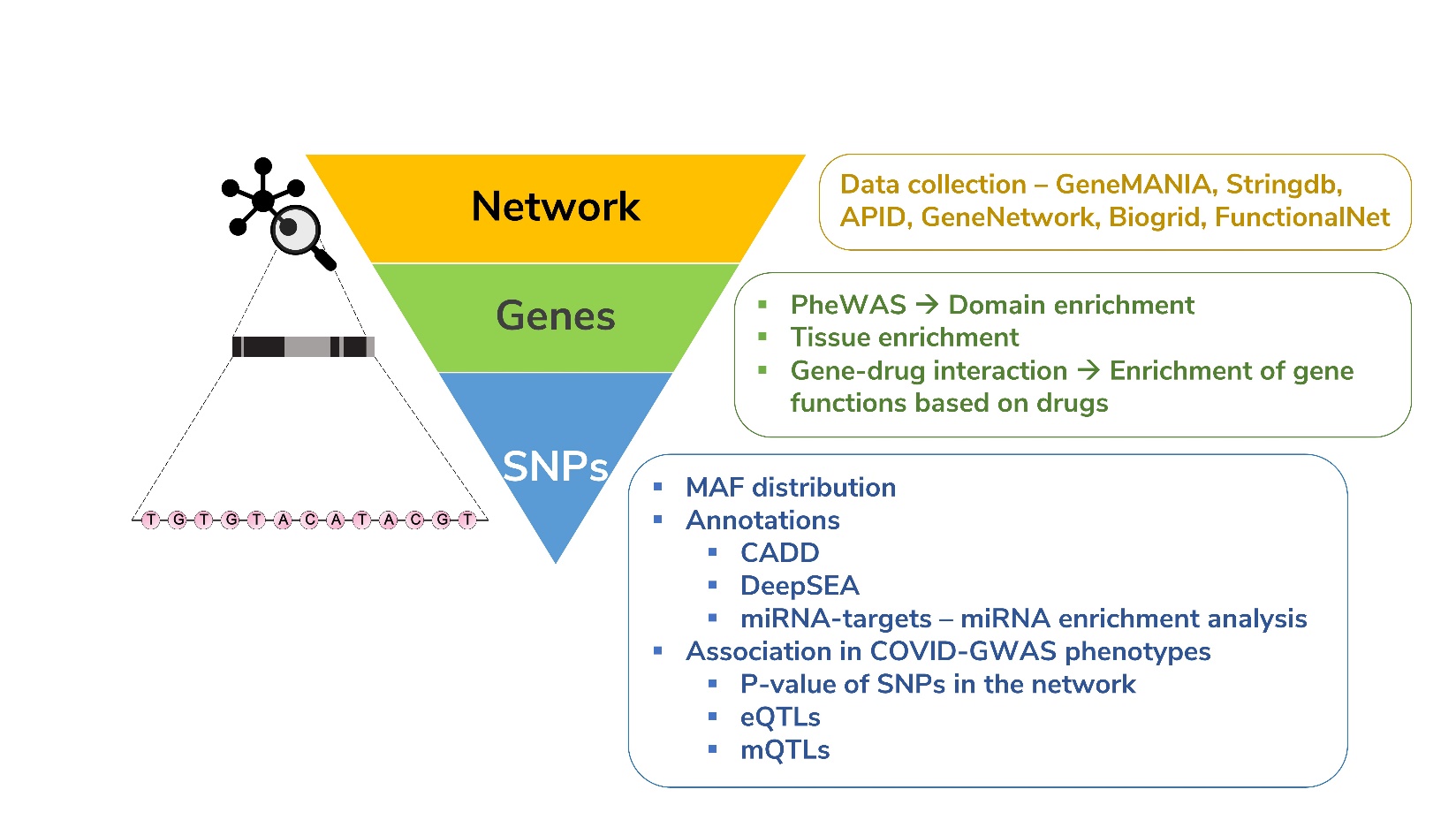


Figure S1: Study Overview. In this study we used a top-down approach to characterize the network of genes that interact with ACE2 gene. First we used several resources to identify which interact with ACE2. Then we performed phenotype associations of each gene using the 4756 phenotypes available in GWAS Atlas. We then performed tissue enrichment based on differential expression of these genes and tested for drugs that interact with the ACE2-gene network. Finally, we extracted SNPs positionally within 10kb of each gene and obtained functional scores using CADD and DeepSEA scores, followed by miRNA-SNP relationship to identify non-coding regulatory consequences. Lastly, SNPs were extracted from the COVID-19 GWAS association statistics of six COVID-19 susceptibility phenotypes to derive LD-independent and p-value clumped SNPs. The significant SNPs were tested for their gene (eQTLs) and methylation associations (mQTLs).


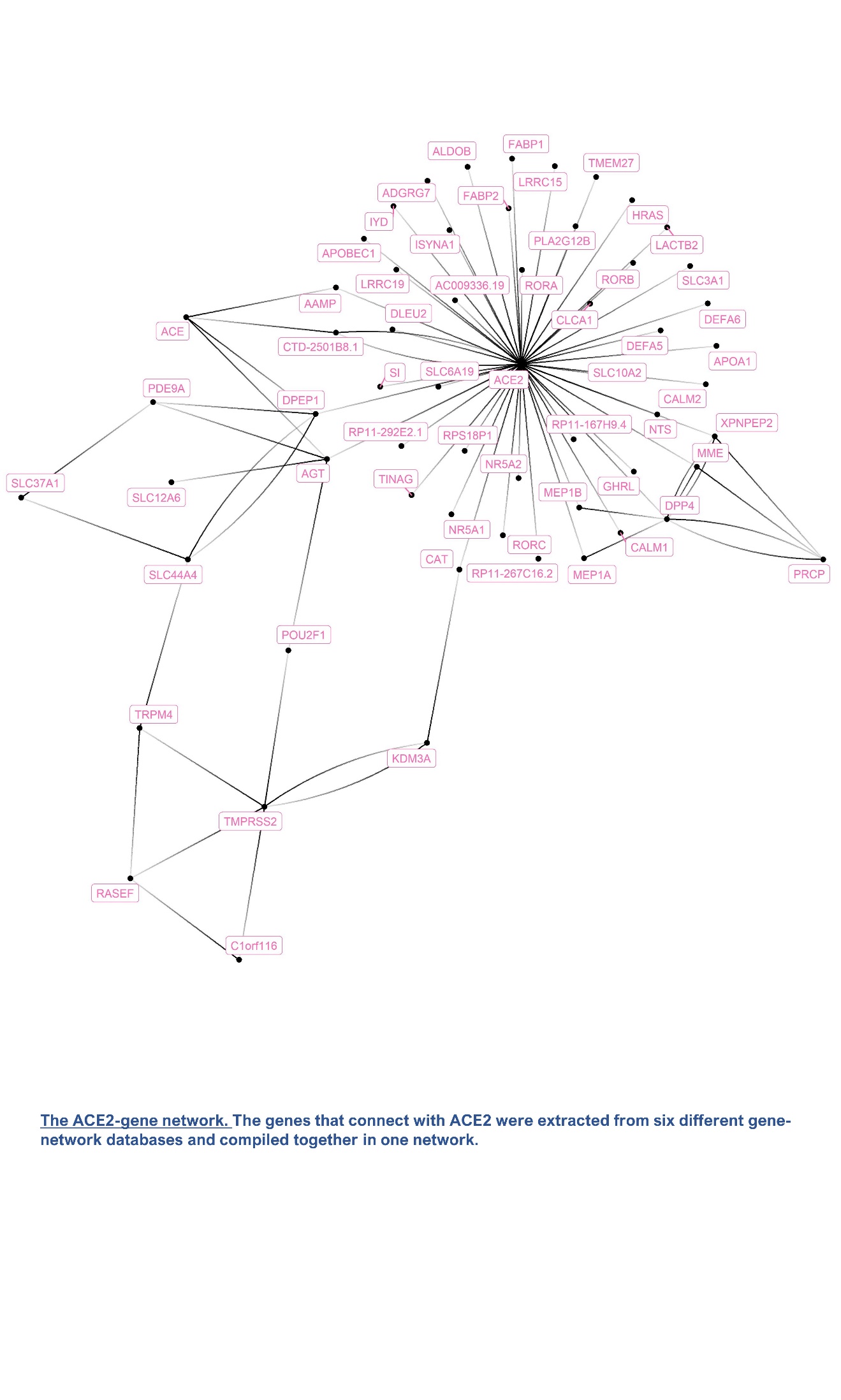


# PheWAS of genes in the ACE2 network


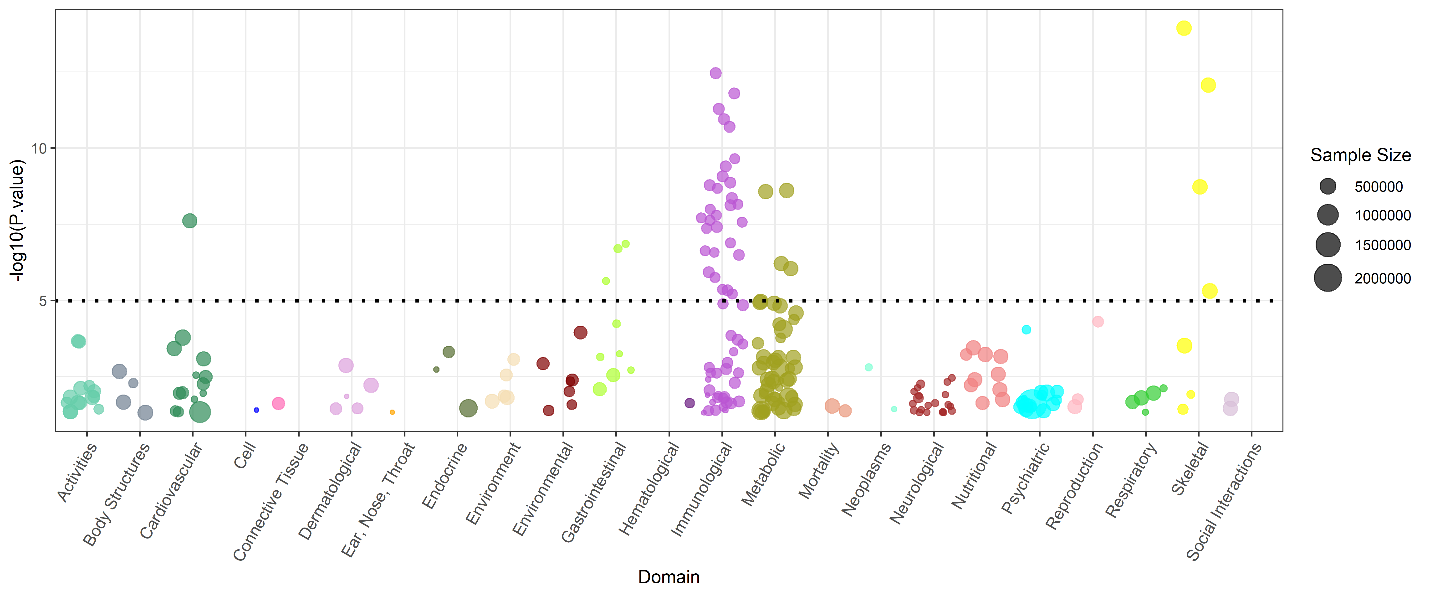


Figure S2:AAMP. Each data point presents trait associated with gene as mined from the GWAS Atlas, traits are grouped in domains (x-axis) and size of the data point represents the sample size (legend on right) of the study for which the association statistic was reported. The y-axis shows -log10(p-value) of the gene with the respective trait. The dotted line presents Bonferroni significance line (1e-5) correcting for the traits present in the GWASatlas.


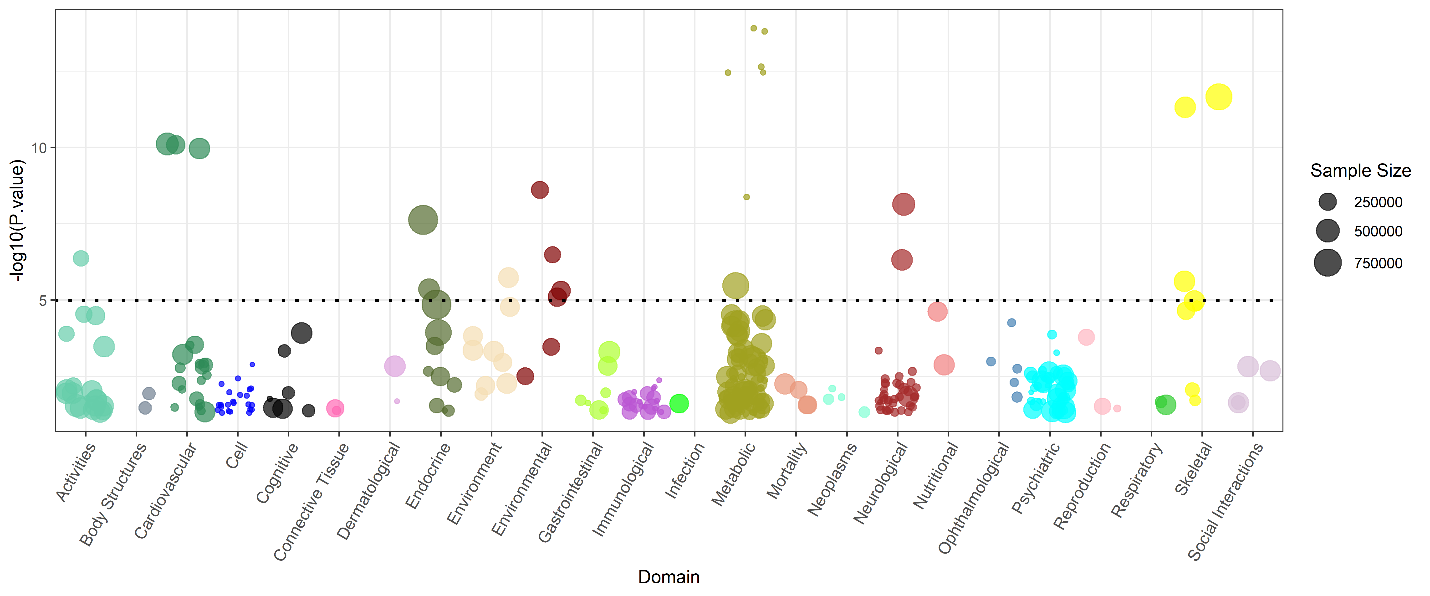


Figure S3: ACE. Each data point presents trait associated with gene as mined from the GWAS Atlas, traits are grouped in domains (x-axis) and size of the data point represents the sample size (legend on right) of the study for which the association statistic was reported. The y-axis shows -log10(p-value) of the gene with the respective trait. The dotted line presents Bonferroni significance line (1e-5) correcting for the traits present in the GWASatlas.


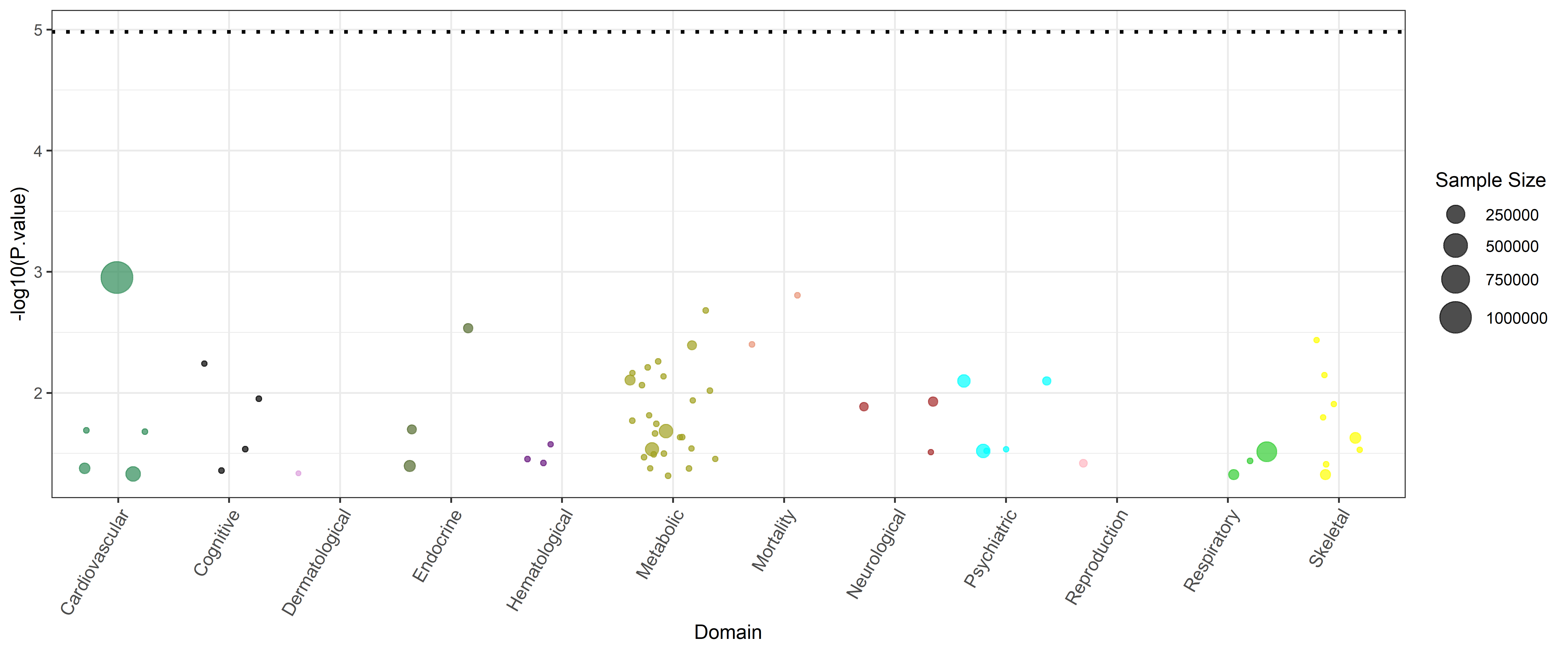


Figure S4: ACE2. Each data point presents trait associated with gene as mined from the GWAS Atlas, traits are grouped in domains (x-axis) and size of the data point represents the sample size (legend on right) of the study for which the association statistic was reported. The y-axis shows -log10(p-value) of the gene with the respective trait. The dotted line presents Bonferroni significance line (1e-5) correcting for the traits present in the GWASatlas.


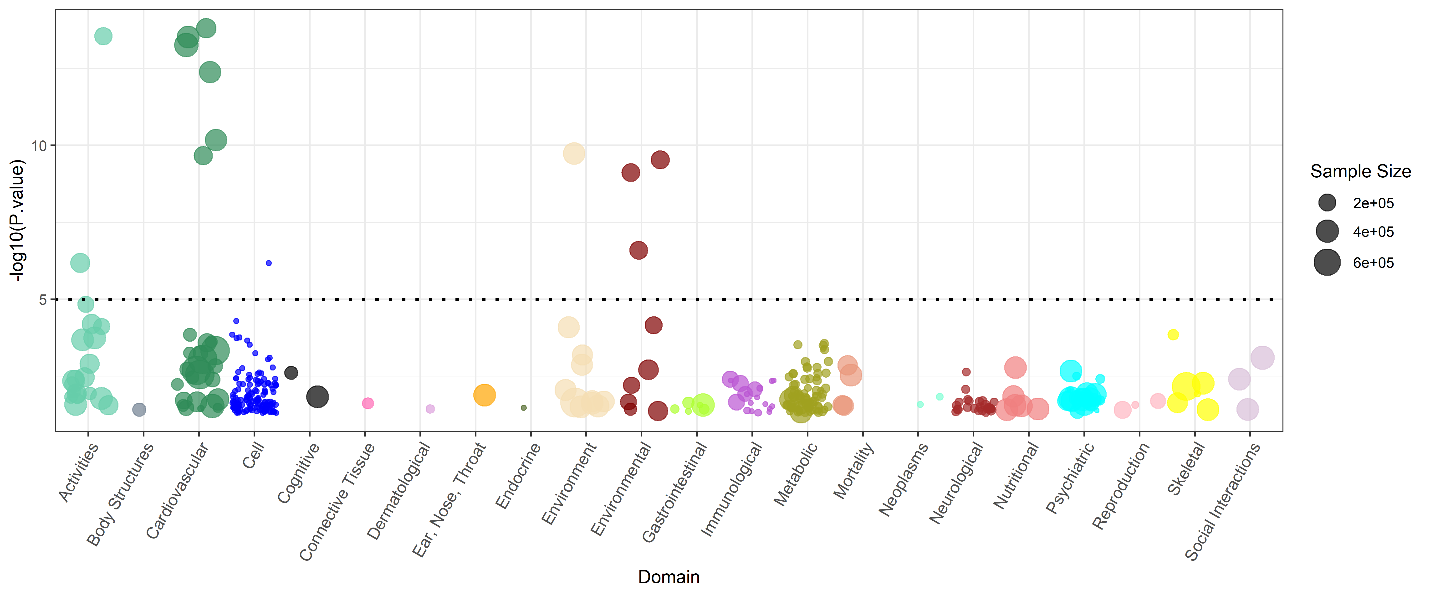


Figure S5: AGT. Each data point presents trait associated with gene as mined from the GWAS Atlas, traits are grouped in domains (x-axis) and size of the data point represents the sample size (legend on right) of the study for which the association statistic was reported. The y-axis shows -log10(p-value) of the gene with the respective trait. The dotted line presents Bonferroni significance line (1e-5) correcting for the traits present in the GWASatlas.


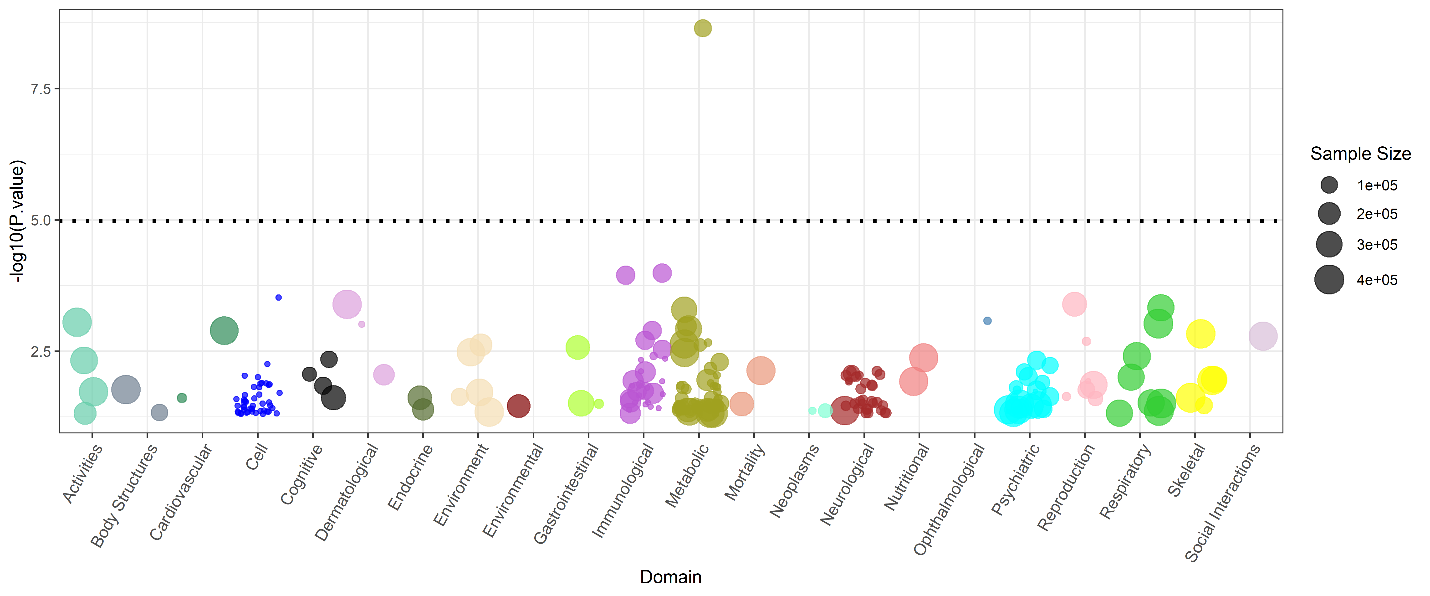


Figure S6: ALDOB. Each data point presents trait associated with gene as mined from the GWAS Atlas, traits are grouped in domains (x-axis) and size of the data point represents the sample size (legend on right) of the study for which the association statistic was reported. The y-axis shows -log10(p-value) of the gene with the respective trait. The dotted line presents Bonferroni significance line (1e-5) correcting for the traits present in the GWASAtlas.


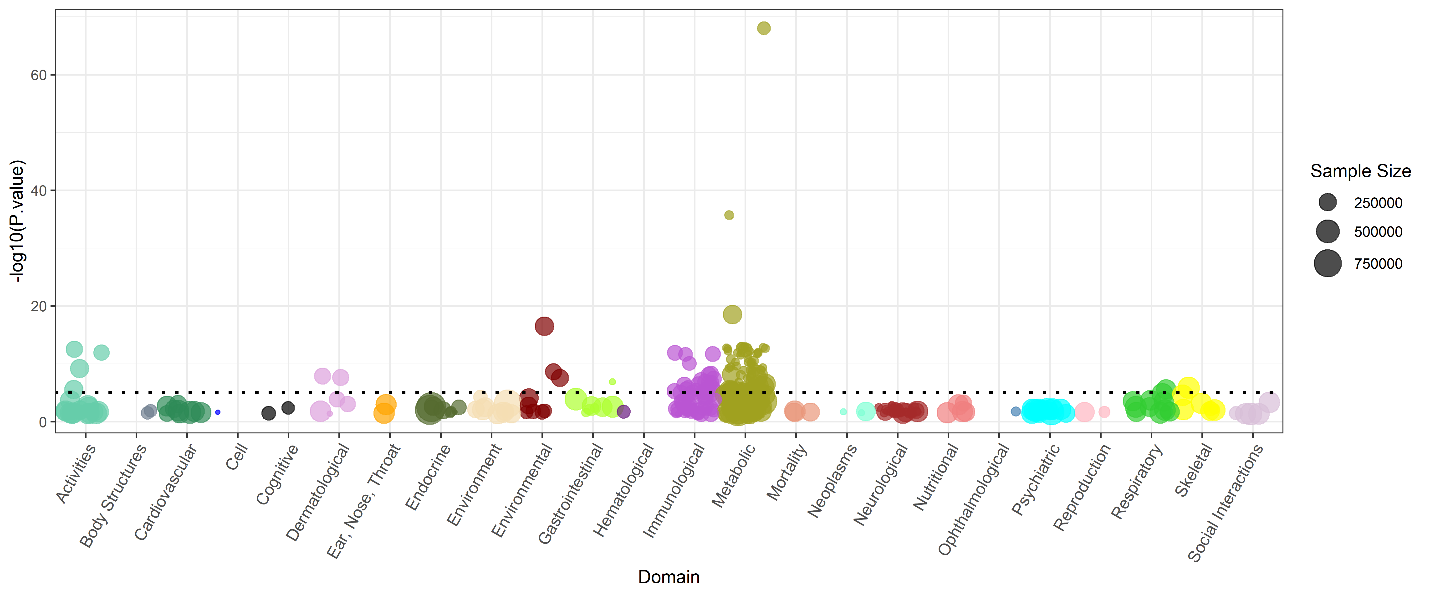


Figure S7: APOA1. Each data point presents trait associated with gene as mined from the GWAS Atlas, traits are grouped in domains (x-axis) and size of the data point represents the sample size (legend on right) of the study for which the association statistic was reported. The y-axis shows -log10(p-value) of the gene with the respective trait. The dotted line presents Bonferroni significance line (1e-5) correcting for the traits present in the GWASAtlas.


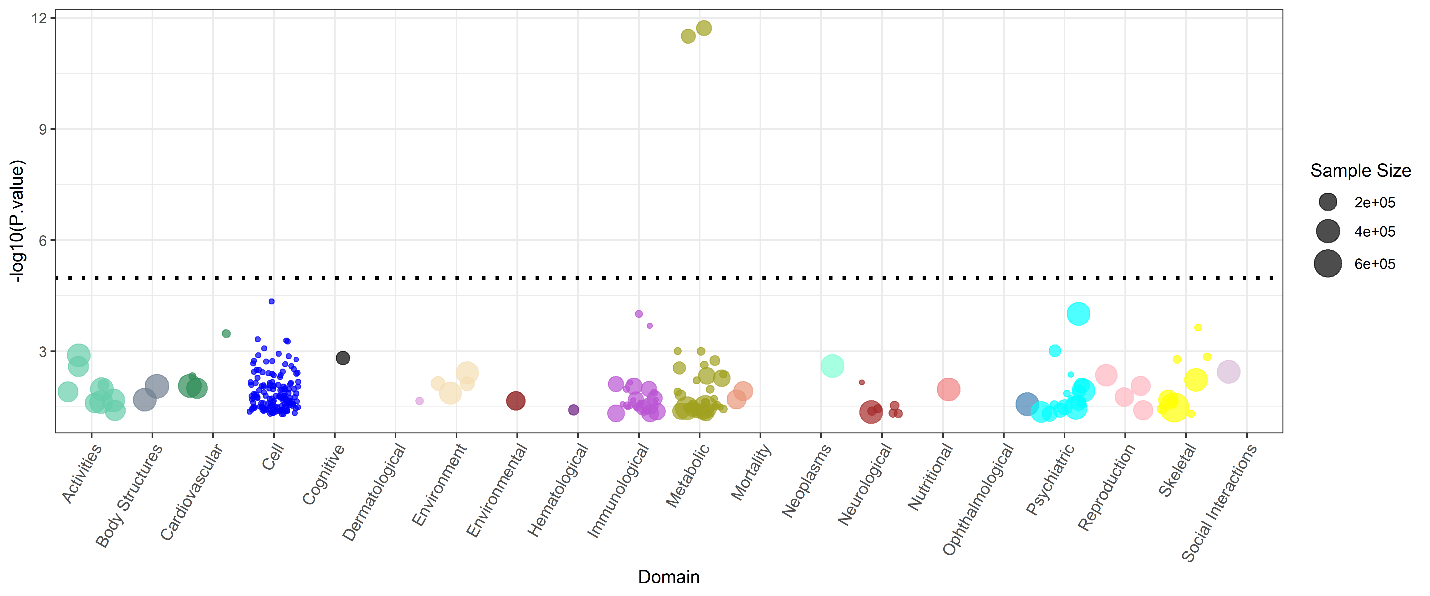


Figure S8: APOBEC1. Each data point presents trait associated with gene as mined from the GWAS Atlas, traits are grouped in domains (x-axis) and size of the data point represents the sample size (legend on right) of the study for which the association statistic was reported. The y-axis shows -log10(p-value) of the gene with the respective trait. The dotted line presents Bonferroni significance line (1e-5) correcting for the traits present in the GWASAtlas.


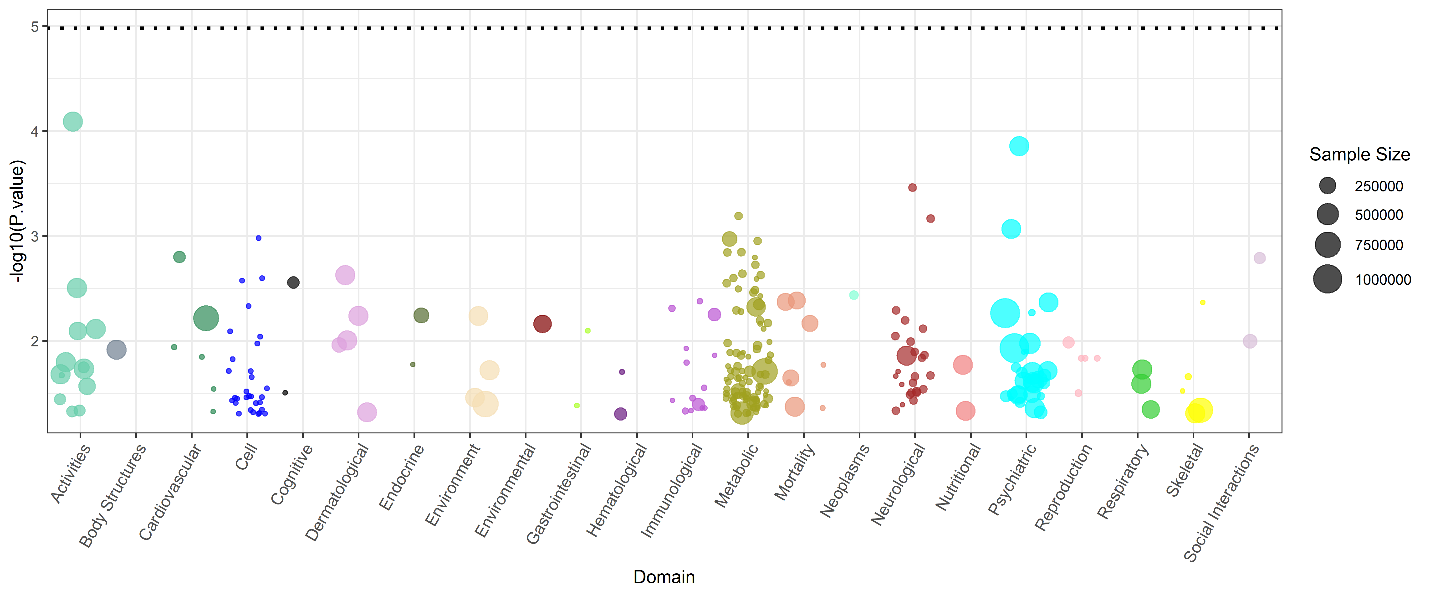


Figure S9: CALM1. Each data point presents trait associated with gene as mined from the GWAS Atlas, traits are grouped in domains (x-axis) and size of the data point represents the sample size (legend on right) of the study for which the association statistic was reported. The y-axis shows -log10(p-value) of the gene with the respective trait. The dotted line presents Bonferroni significance line (1e-5) correcting for the traits present in the GWASAtlas.


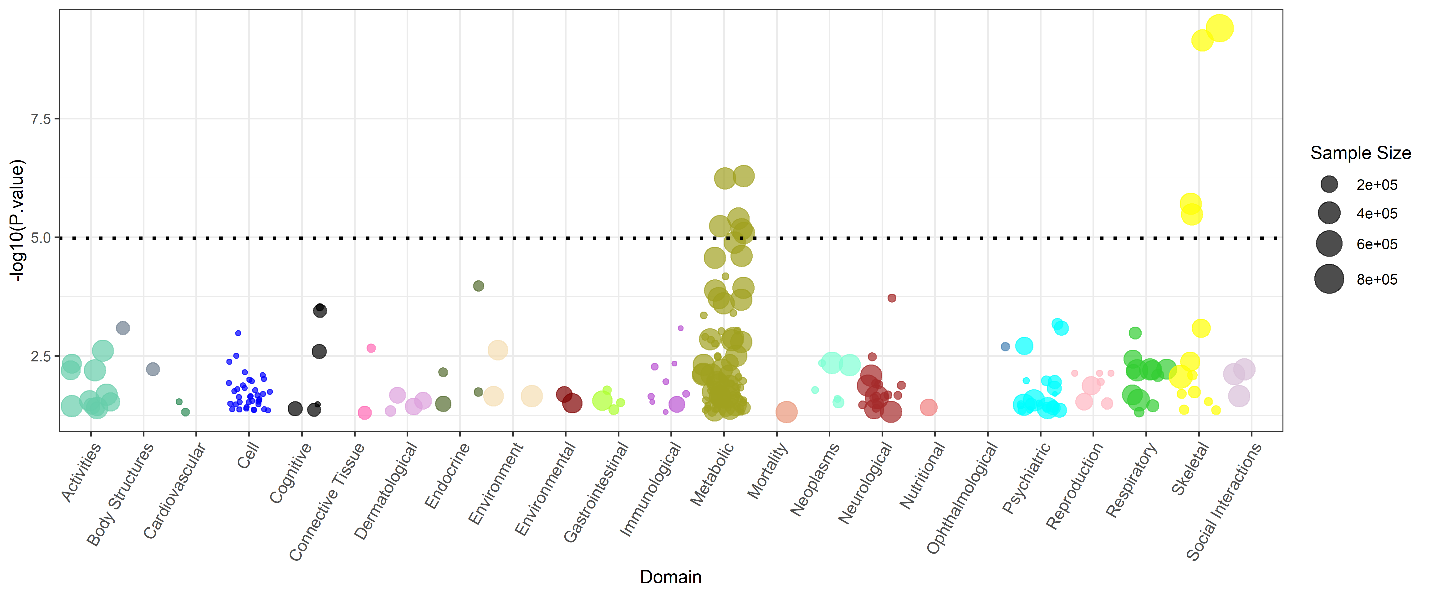


Figure S10: CALM2. Each data point presents trait associated with gene as mined from the GWAS Atlas, traits are grouped in domains (x-axis) and size of the data point represents the sample size (legend on right) of the study for which the association statistic was reported. The y-axis shows -log10(p-value) of the gene with the respective trait. The dotted line presents Bonferroni significance line (1e-5) correcting for the traits present in the GWASAtlas.


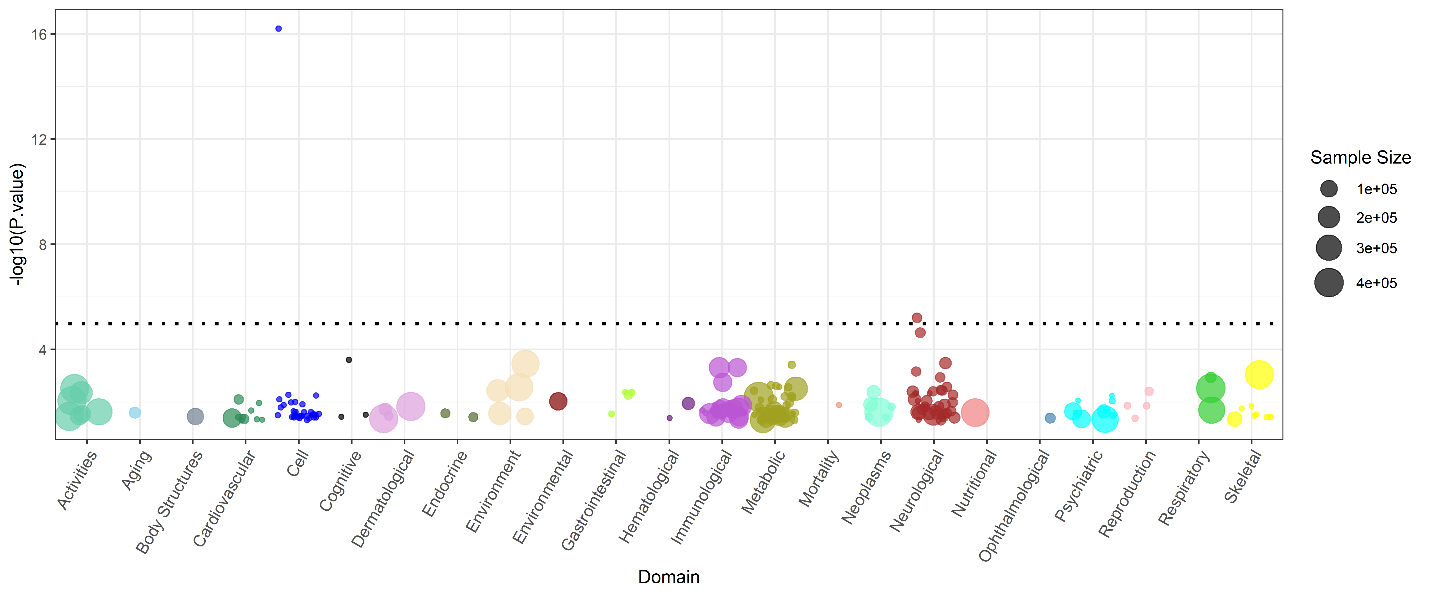


Figure S11: CAT. Each data point presents trait associated with gene as mined from the GWAS Atlas, traits are grouped in domains (x-axis) and size of the data point represents the sample size (legend on right) of the study for which the association statistic was reported. The y-axis shows -log10(p-value) of the gene with the respective trait. The dotted line presents Bonferroni significance line (1e-5) correcting for the traits present in the GWASAtlas.


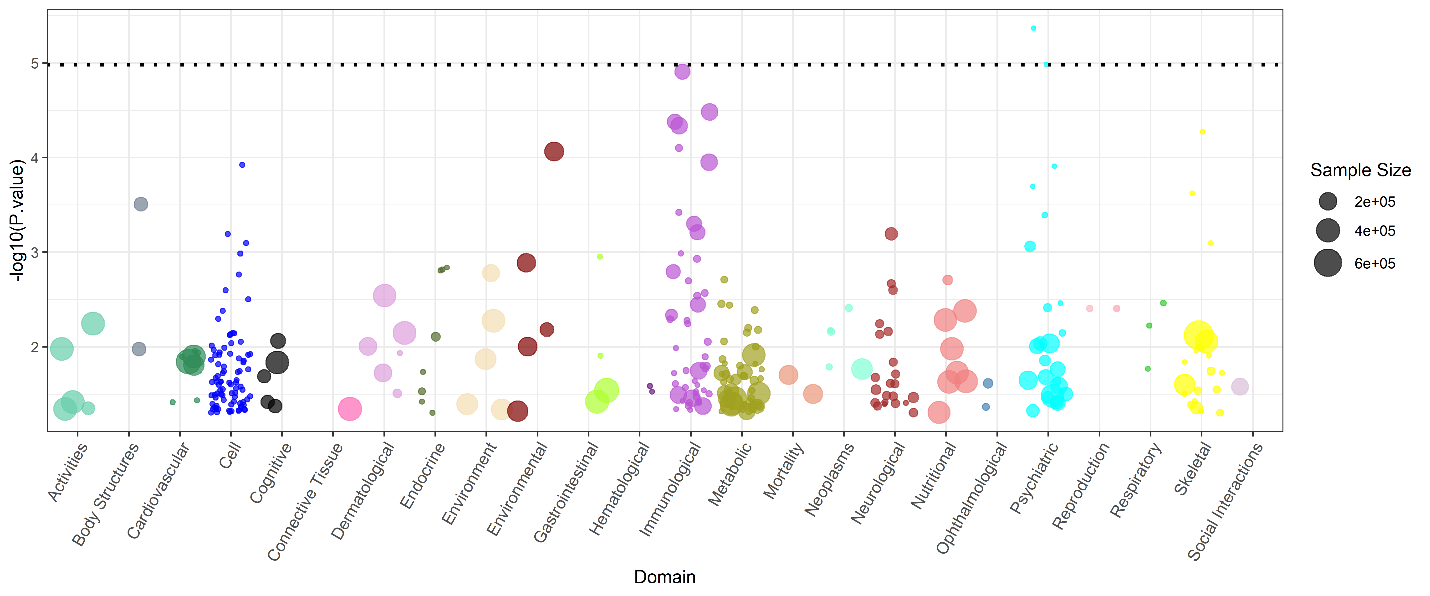


Figure S12: CLCA1. Each data point presents trait associated with gene as mined from the GWAS Atlas, traits are grouped in domains (x-axis) and size of the data point represents the sample size (legend on right) of the study for which the association statistic was reported. The y-axis shows -log10(p-value) of the gene with the respective trait. The dotted line presents Bonferroni significance line (1e-5) correcting for the traits present in the GWASAtlas.


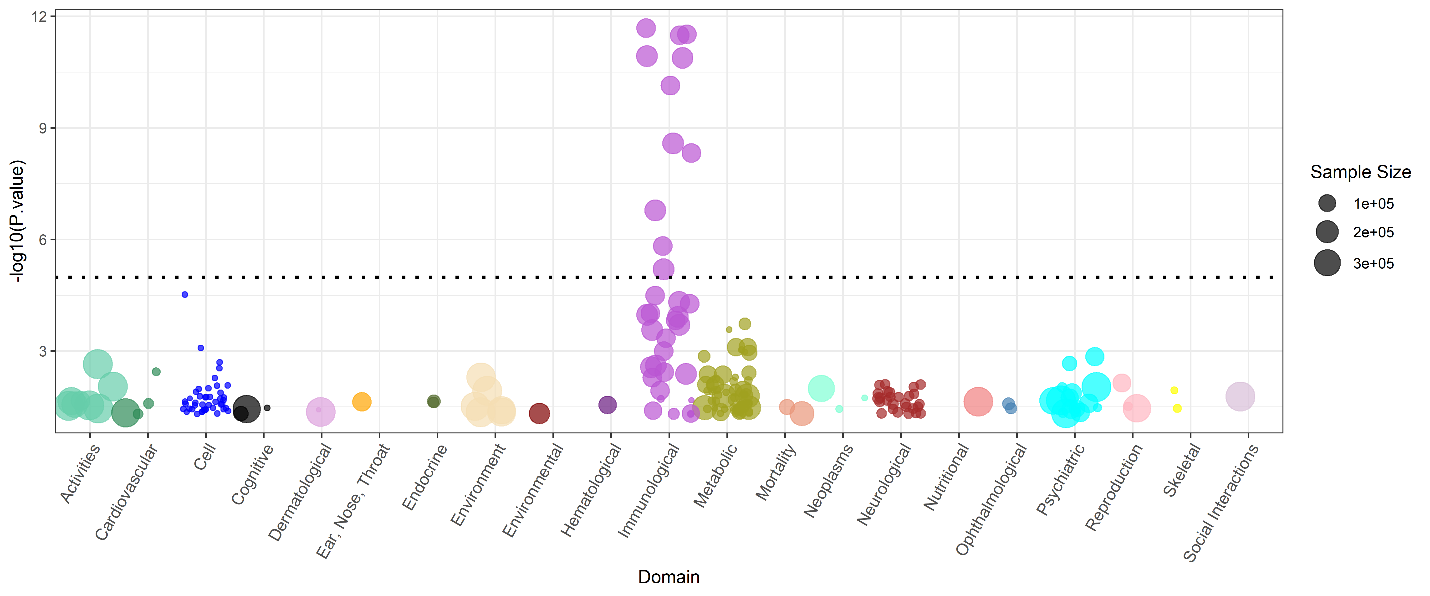


Figure S13: DEFA5. Each data point presents trait associated with gene as mined from the GWAS Atlas, traits are grouped in domains (x-axis) and size of the data point represents the sample size (legend on right) of the study for which the association statistic was reported. The y-axis shows -log10(p-value) of the gene with the respective trait. The dotted line presents Bonferroni significance line (1e-5) correcting for the traits present in the GWASAtlas.


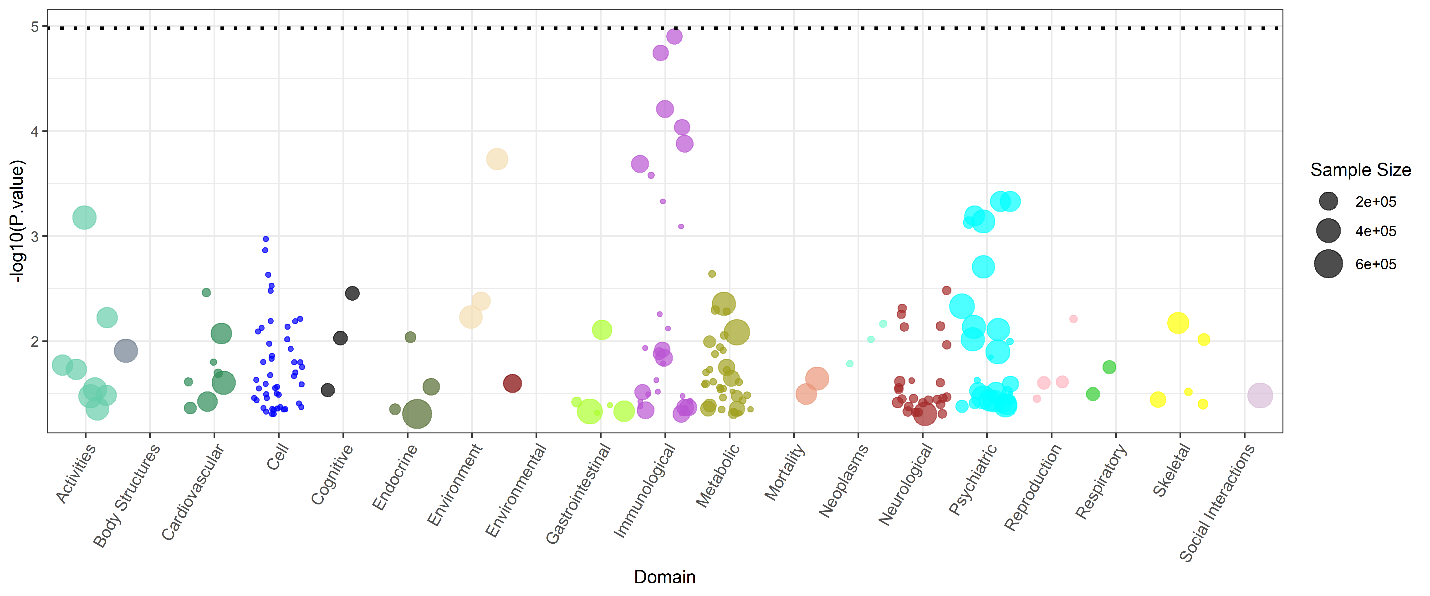


Figure S14: DEFA6. Each data point presents trait associated with gene as mined from the GWAS Atlas, traits are grouped in domains (x-axis) and size of the data point represents the sample size (legend on right) of the study for which the association statistic was reported. The y-axis shows -log10(p-value) of the gene with the respective trait. The dotted line presents Bonferroni significance line (1e-5) correcting for the traits present in the GWASAtlas.


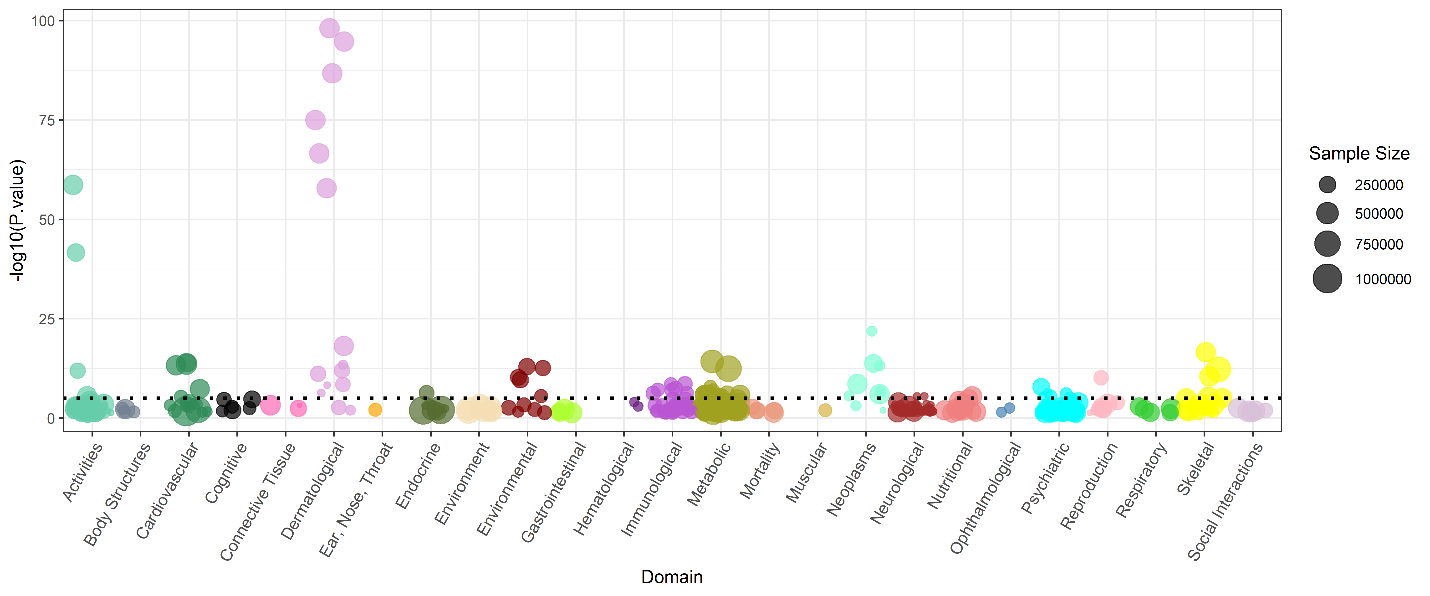


Figure S15: DPEP1. Each data point presents trait associated with gene as mined from the GWAS Atlas, traits are grouped in domains (x-axis) and size of the data point represents the sample size (legend on right) of the study for which the association statistic was reported. The y-axis shows -log10(p-value) of the gene with the respective trait. The dotted line presents Bonferroni significance line (1e-5) correcting for the traits present in the GWASAtlas.


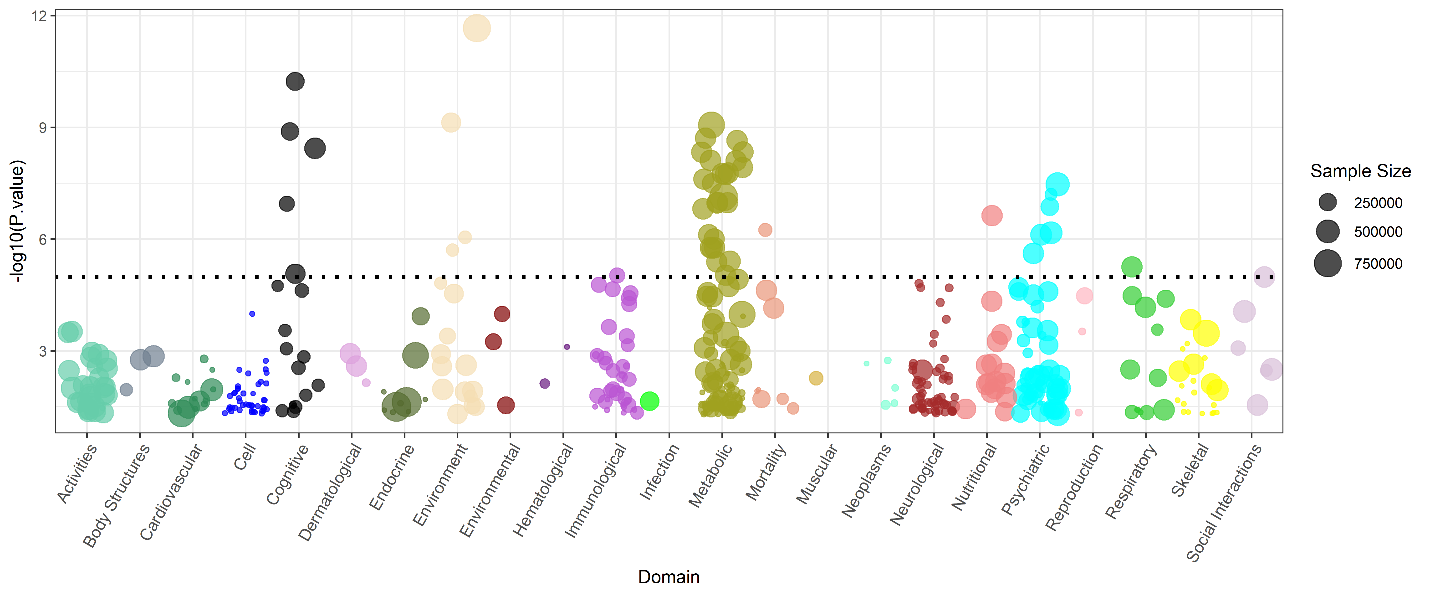


Figure S16: DPP4. Each data point presents trait associated with gene as mined from the GWAS Atlas, traits are grouped in domains (x-axis) and size of the data point represents the sample size (legend on right) of the study for which the association statistic was reported. The y-axis shows -log10(p-value) of the gene with the respective trait. The dotted line presents Bonferroni significance line (1e-5) correcting for the traits present in the GWASAtlas.


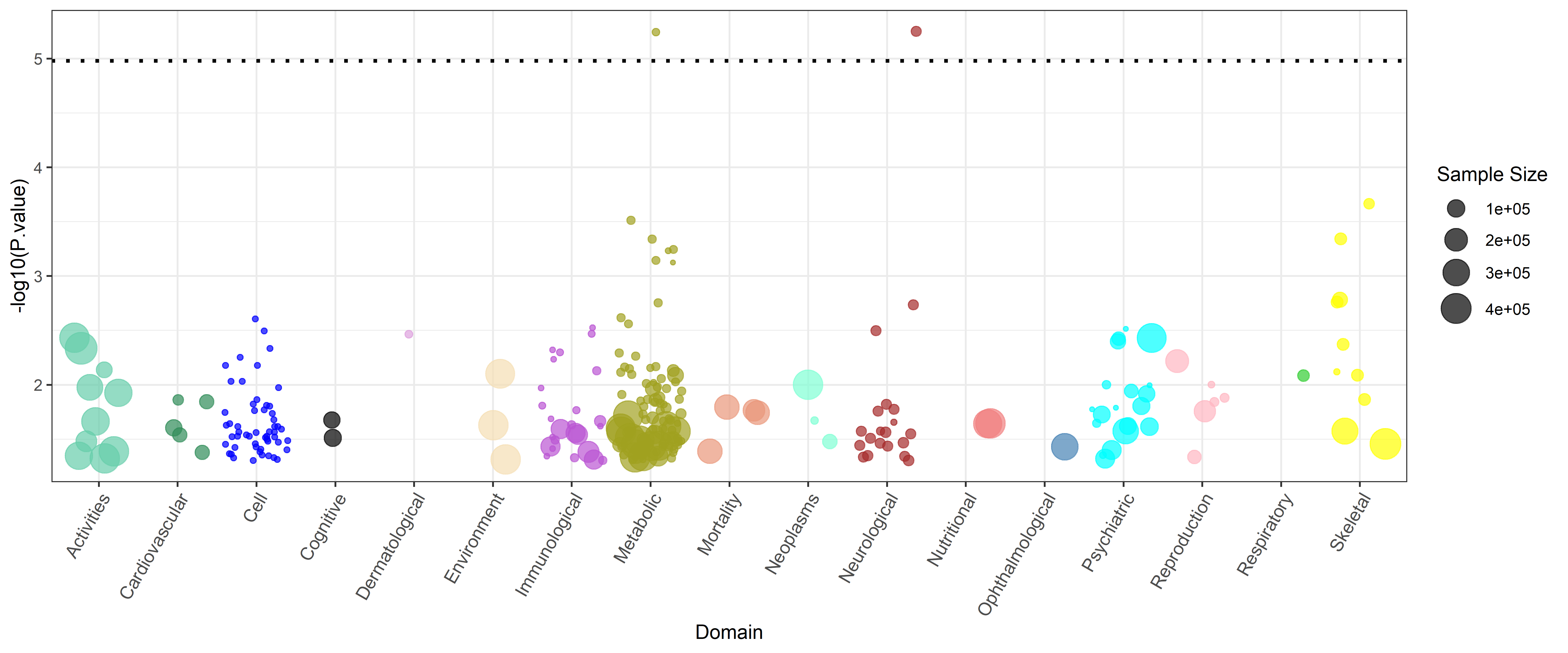


Figure S17: FABP1. Each data point presents trait associated with gene as mined from the GWAS Atlas, traits are grouped in domains (x-axis) and size of the data point represents the sample size (legend on right) of the study for which the association statistic was reported. The y-axis shows -log10(p-value) of the gene with the respective trait. The dotted line presents Bonferroni significance line (1e-5) correcting for the traits present in the GWASAtlas.


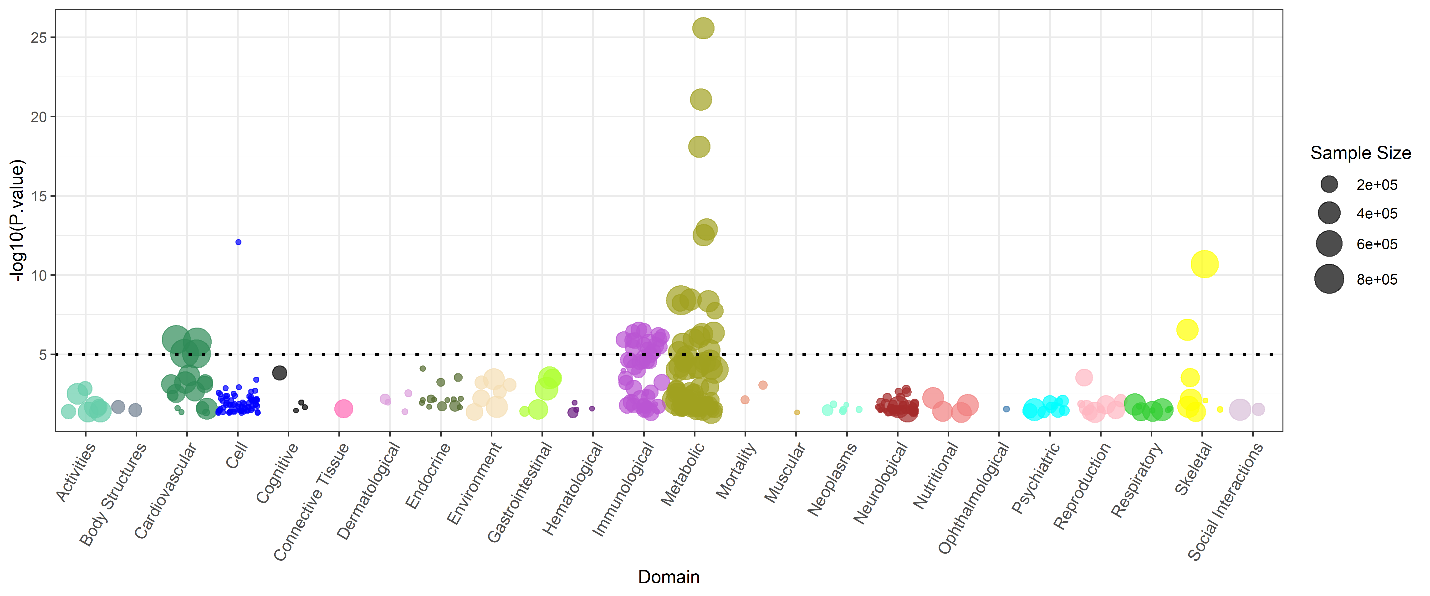


Figure S18:FABP2. Each data point presents trait associated with gene as mined from the GWAS Atlas, traits are grouped in domains (x-axis) and size of the data point represents the sample size (legend on right) of the study for which the association statistic was reported. The y-axis shows -log10(p-value) of the gene with the respective trait. The dotted line presents Bonferroni significance line (1e-5) correcting for the traits present in the GWASAtlas.


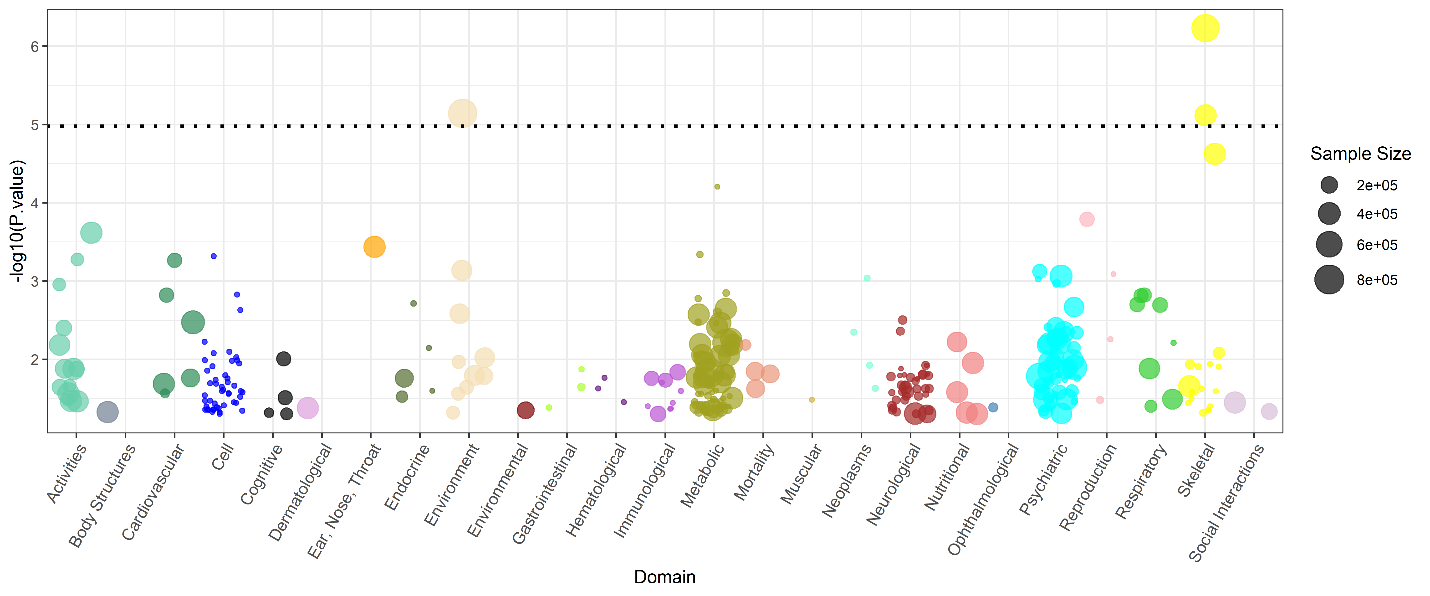


Figure S19: GHRL. Each data point presents trait associated with gene as mined from the GWAS Atlas, traits are grouped in domains (x-axis) and size of the data point represents the sample size (legend on right) of the study for which the association statistic was reported. The y-axis shows -log10(p-value) of the gene with the respective trait. The dotted line presents Bonferroni significance line (1e-5) correcting for the traits present in the GWASAtlas.


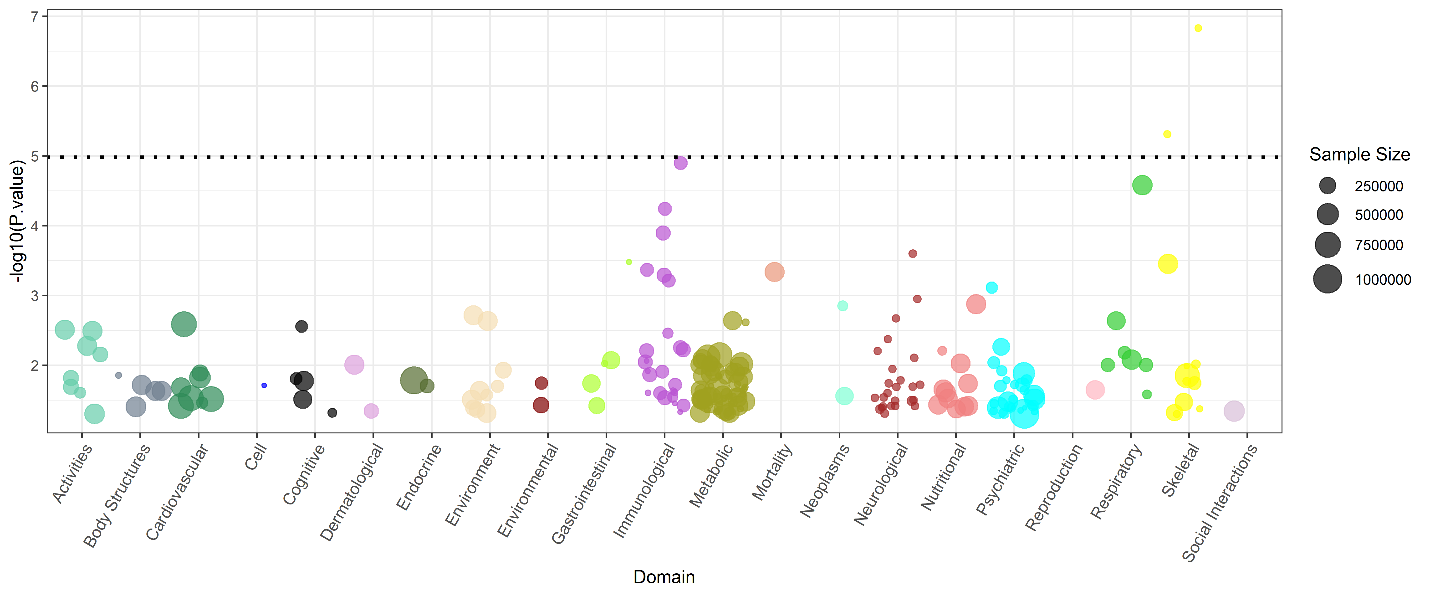


Figure S20: HRAS. Each data point presents trait associated with gene as mined from the GWAS Atlas, traits are grouped in domains (x-axis) and size of the data point represents the sample size (legend on right) of the study for which the association statistic was reported. The y-axis shows -log10(p-value) of the gene with the respective trait. The dotted line presents Bonferroni significance line (1e-5) correcting for the traits present in the GWASAtlas.


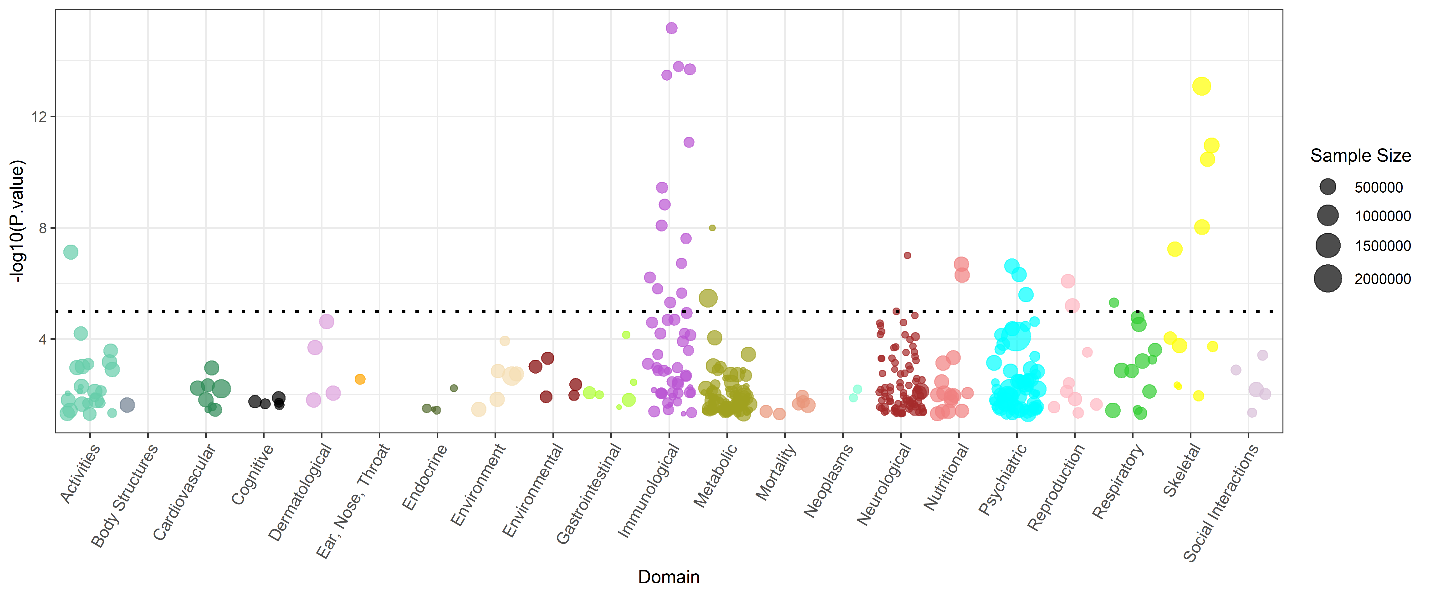


Figure S21:ISYNA1. Each data point presents trait associated with gene as mined from the GWAS Atlas, traits are grouped in domains (x-axis) and size of the data point represents the sample size (legend on right) of the study for which the association statistic was reported. The y-axis shows -log10(p-value) of the gene with the respective trait. The dotted line presents Bonferroni significance line (1e-5) correcting for the traits present in the GWASAtlas.


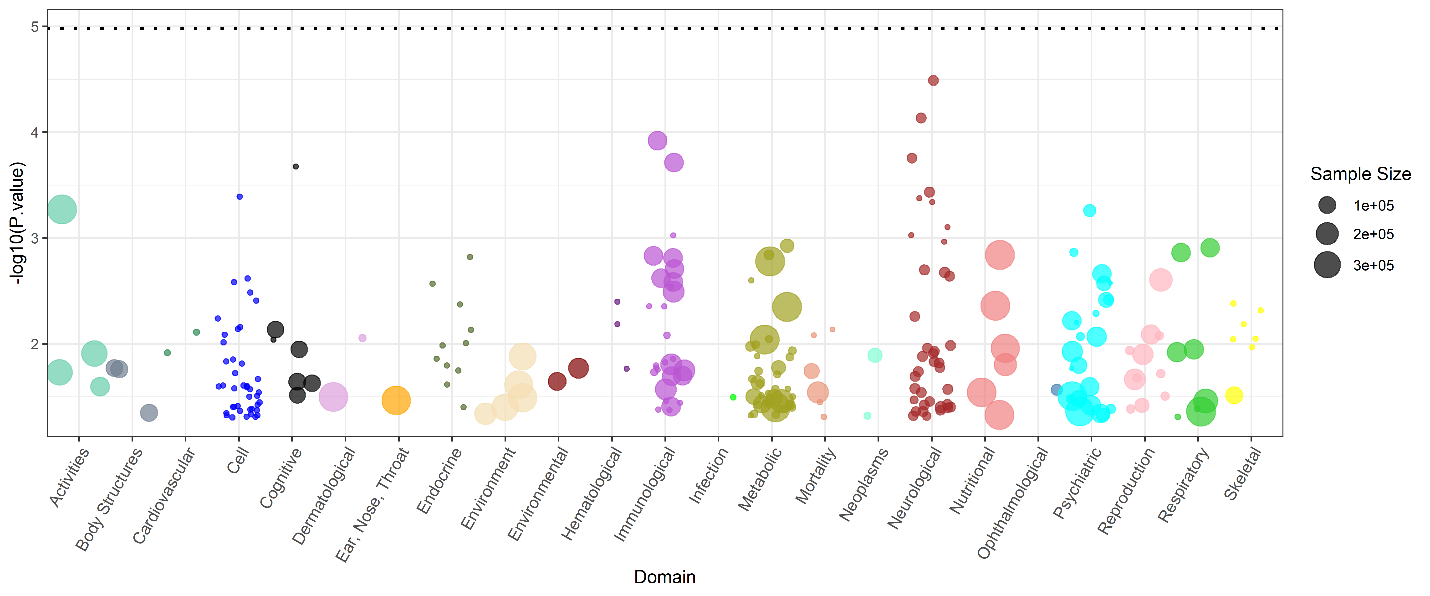


Figure S22:IYD. Each data point presents trait associated with gene as mined from the GWAS Atlas, traits are grouped in domains (x-axis) and size of the data point represents the sample size (legend on right) of the study for which the association statistic was reported. The y-axis shows -log10(p-value) of the gene with the respective trait. The dotted line presents Bonferroni significance line (1e-5) correcting for the traits present in the GWASAtlas.


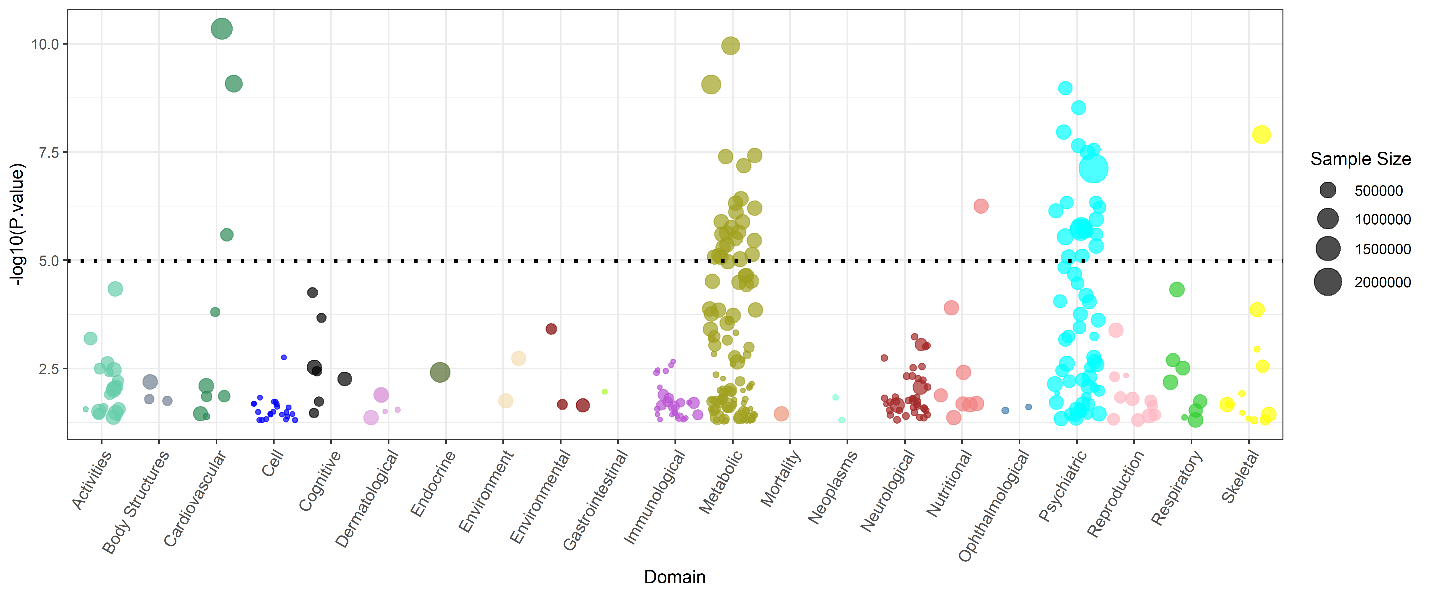


Figure S23: KDM3A. Each data point presents trait associated with gene as mined from the GWAS Atlas, traits are grouped in domains (x-axis) and size of the data point represents the sample size (legend on right) of the study for which the association statistic was reported. The y-axis shows -log10(p-value) of the gene with the respective trait. The dotted line presents Bonferroni significance line (1e-5) correcting for the traits present in the GWASAtlas.


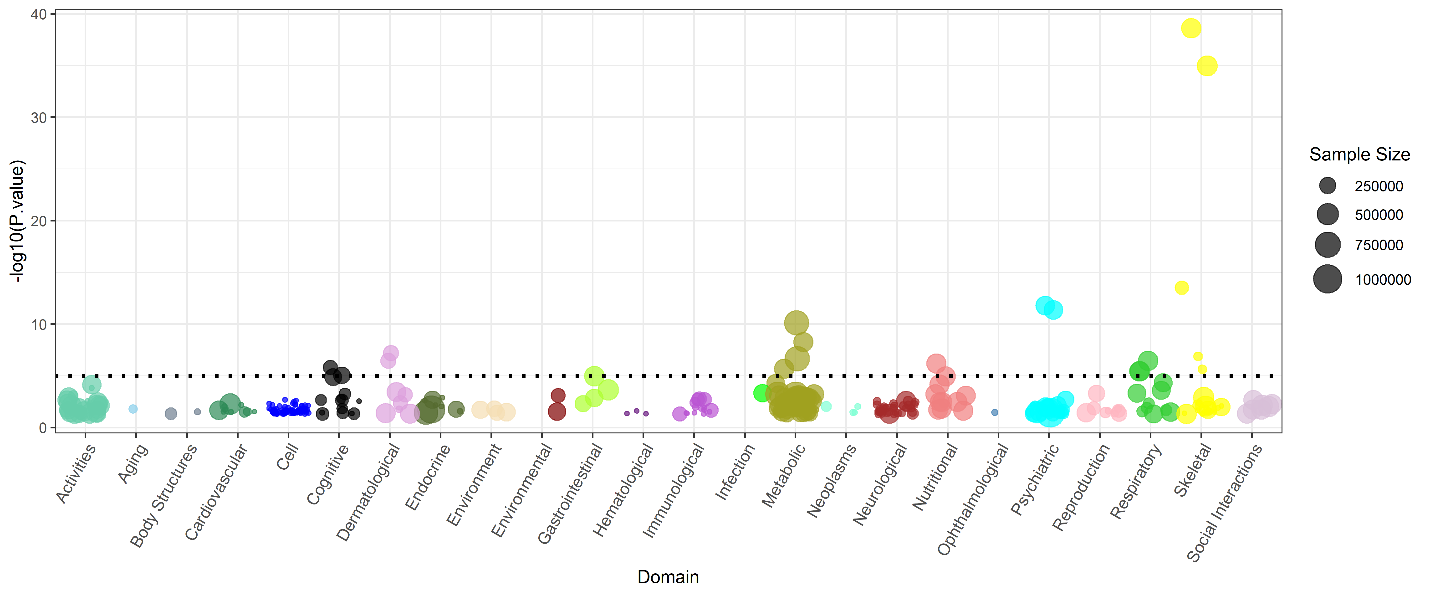


Figure S24: LACTB2. Each data point presents trait associated with gene as mined from the GWAS Atlas, traits are grouped in domains (x-axis) and size of the data point represents the sample size (legend on right) of the study for which the association statistic was reported. The y-axis shows -log10(p-value) of the gene with the respective trait. The dotted line presents Bonferroni significance line (1e-5) correcting for the traits present in the GWASAtlas.


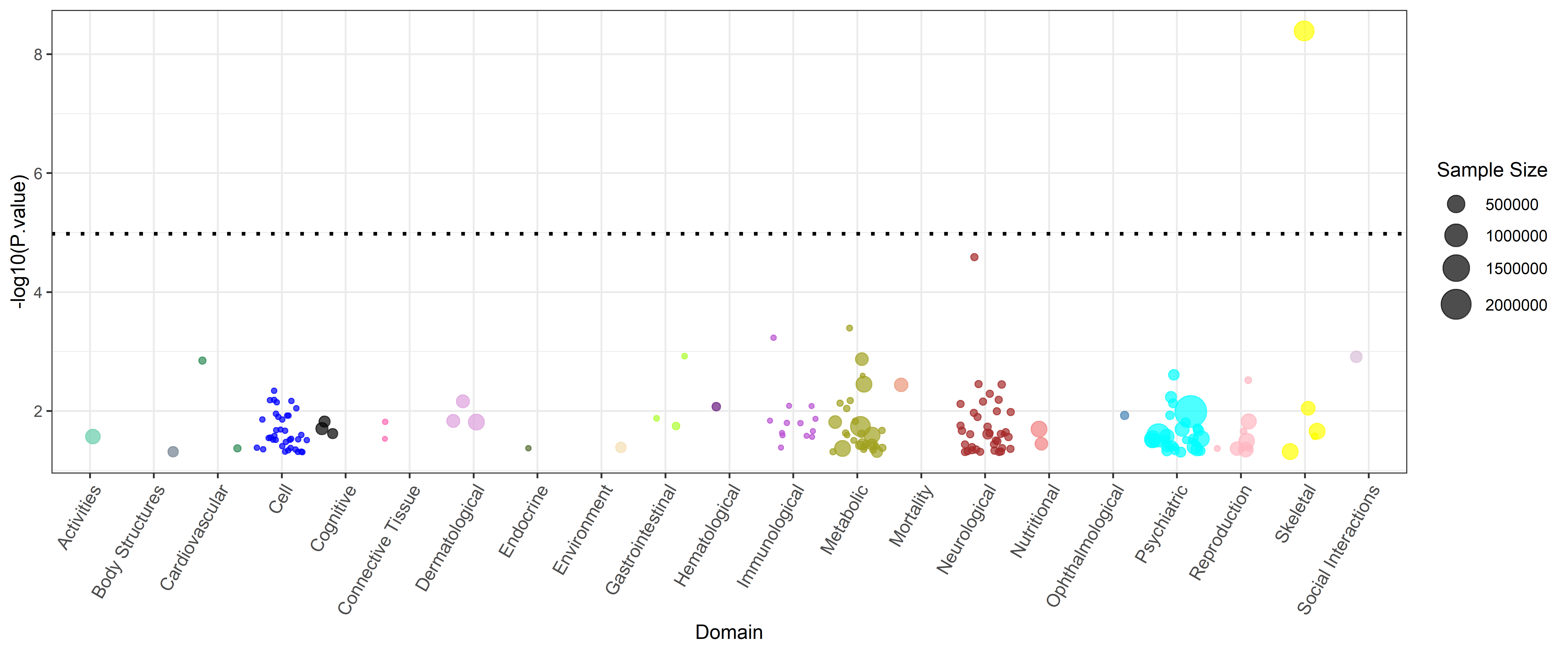


Figure S25:LRRC15. Each data point presents trait associated with gene as mined from the GWAS Atlas, traits are grouped in domains (x-axis) and size of the data point represents the sample size (legend on right) of the study for which the association statistic was reported. The y-axis shows -log10(p-value) of the gene with the respective trait. The dotted line presents Bonferroni significance line (1e-5) correcting for the traits present in the GWASAtlas.


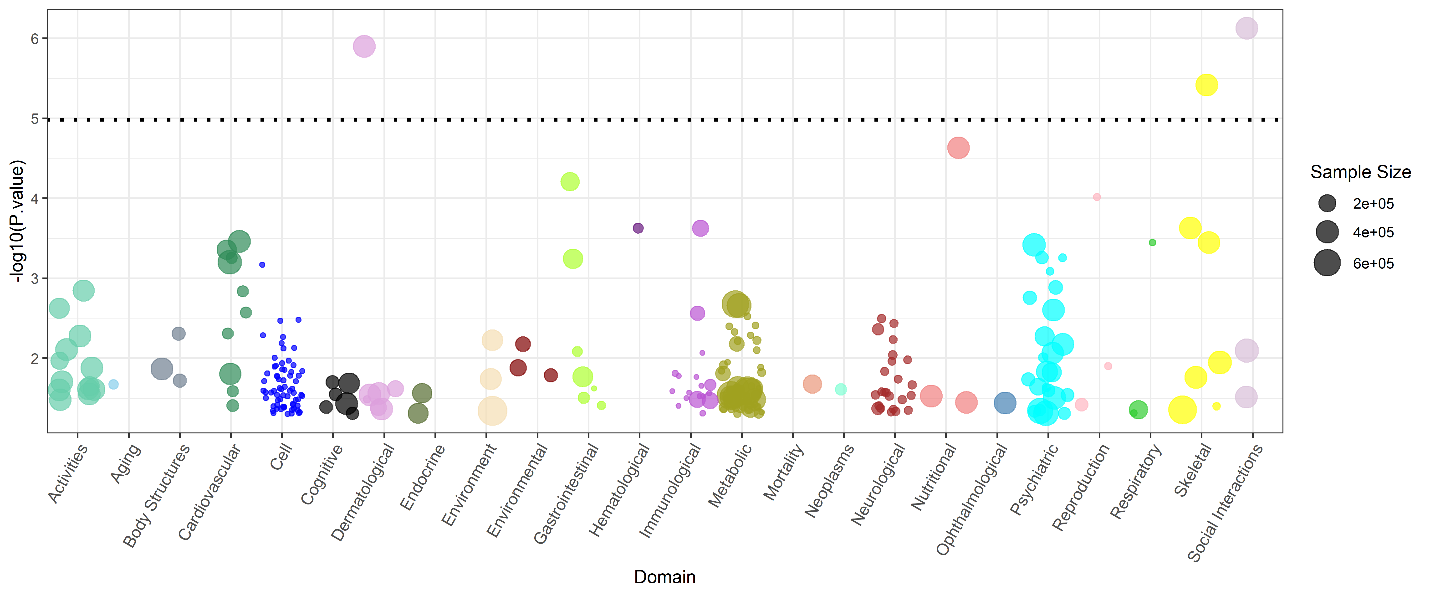


Figure S26: LRRC19. Each data point presents trait associated with gene as mined from the GWAS Atlas, traits are grouped in domains (x-axis) and size of the data point represents the sample size (legend on right) of the study for which the association statistic was reported. The y-axis shows -log10(p-value) of the gene with the respective trait. The dotted line presents Bonferroni significance line (1e-5) correcting for the traits present in the GWASAtlas.


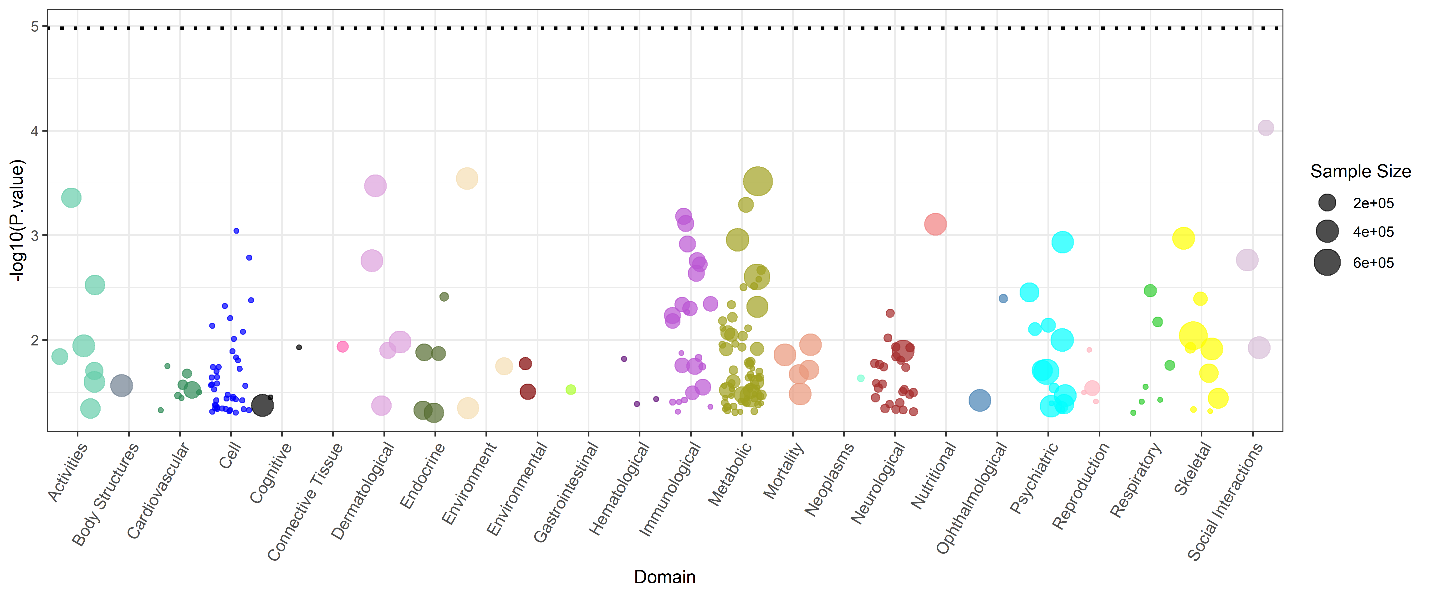


Figure S27: MEP1A. Each data point presents trait associated with gene as mined from the GWAS Atlas, traits are grouped in domains (x-axis) and size of the data point represents the sample size (legend on right) of the study for which the association statistic was reported. The y-axis shows -log10(p-value) of the gene with the respective trait. The dotted line presents Bonferroni significance line (1e-5) correcting for the traits present in the GWASAtlas.


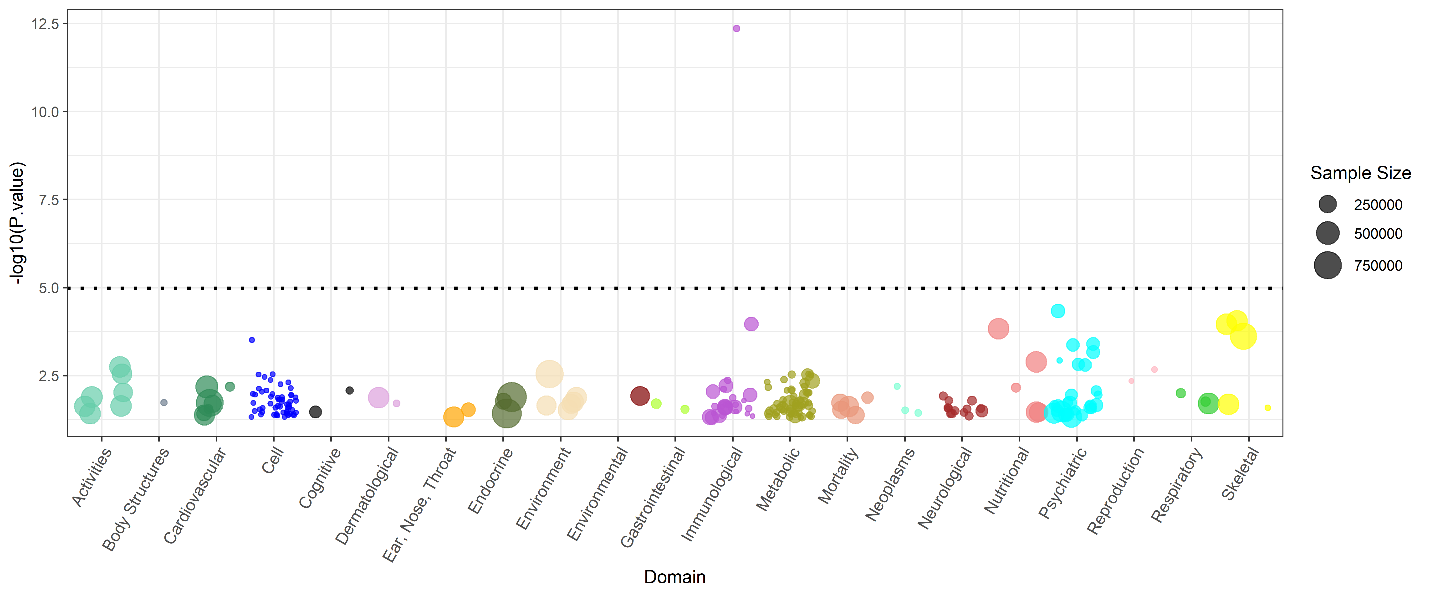


Figure S28:MEP1B. Each data point presents trait associated with gene as mined from the GWAS Atlas, traits are grouped in domains (x-axis) and size of the data point represents the sample size (legend on right) of the study for which the association statistic was reported. The y-axis shows -log10(p-value) of the gene with the respective trait. The dotted line presents Bonferroni significance line (1e-5) correcting for the traits present in the GWASAtlas.


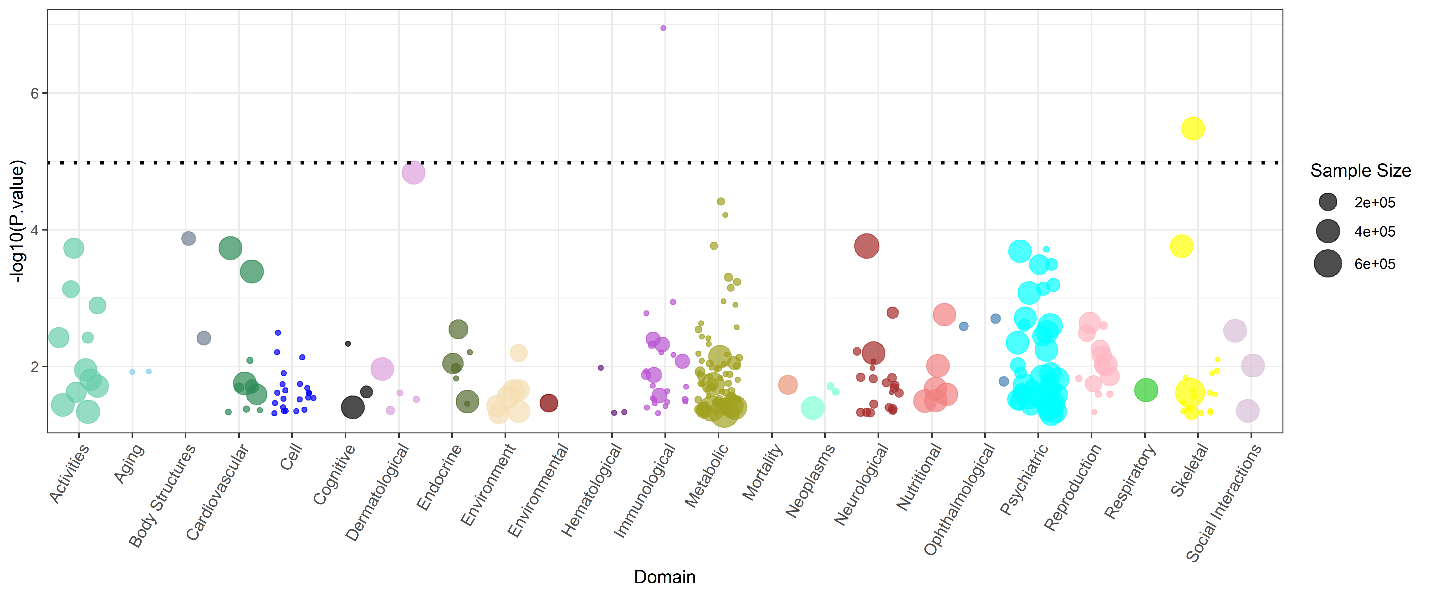


Figure S29: MME. Each data point presents trait associated with gene as mined from the GWAS Atlas, traits are grouped in domains (x-axis) and size of the data point represents the sample size (legend on right) of the study for which the association statistic was reported. The y-axis shows -log10(p-value) of the gene with the respective trait. The dotted line presents Bonferroni significance line (1e-5) correcting for the traits present in the GWASAtlas.


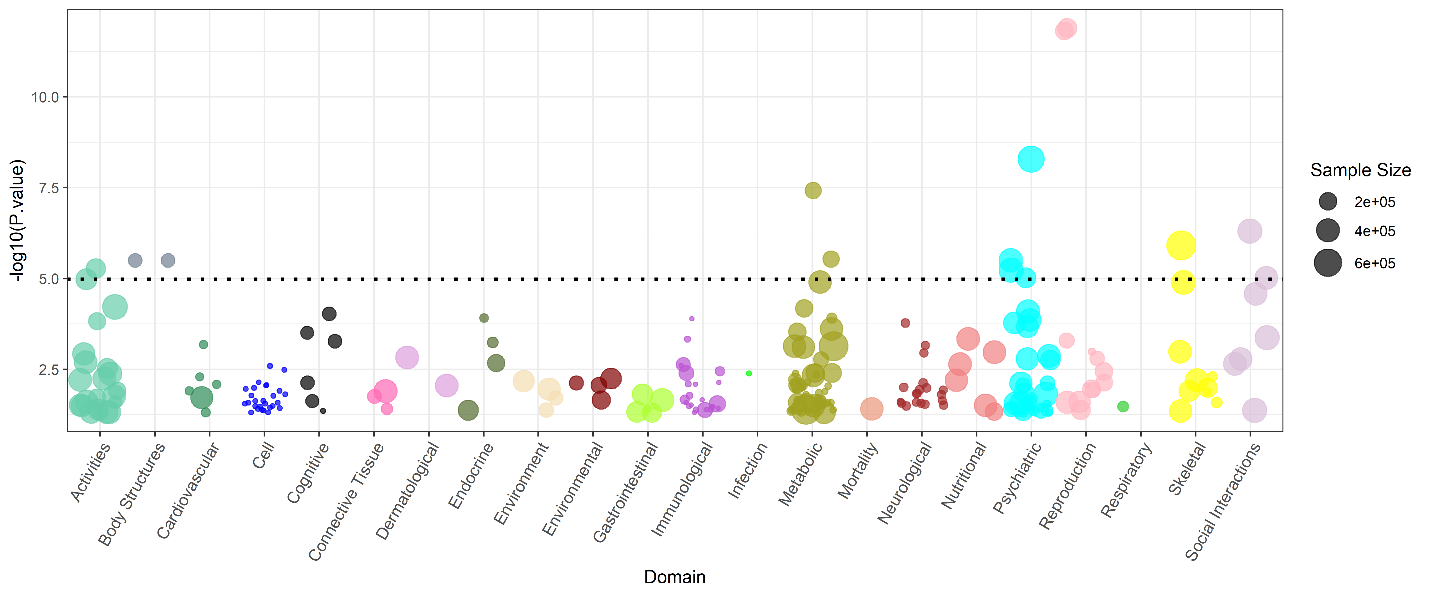


Figure S30: NR5A1. Each data point presents trait associated with gene as mined from the GWAS Atlas, traits are grouped in domains (x-axis) and size of the data point represents the sample size (legend on right) of the study for which the association statistic was reported. The y-axis shows -log10(p-value) of the gene with the respective trait. The dotted line presents Bonferroni significance line (1e-5) correcting for the traits present in the GWASAtlas.


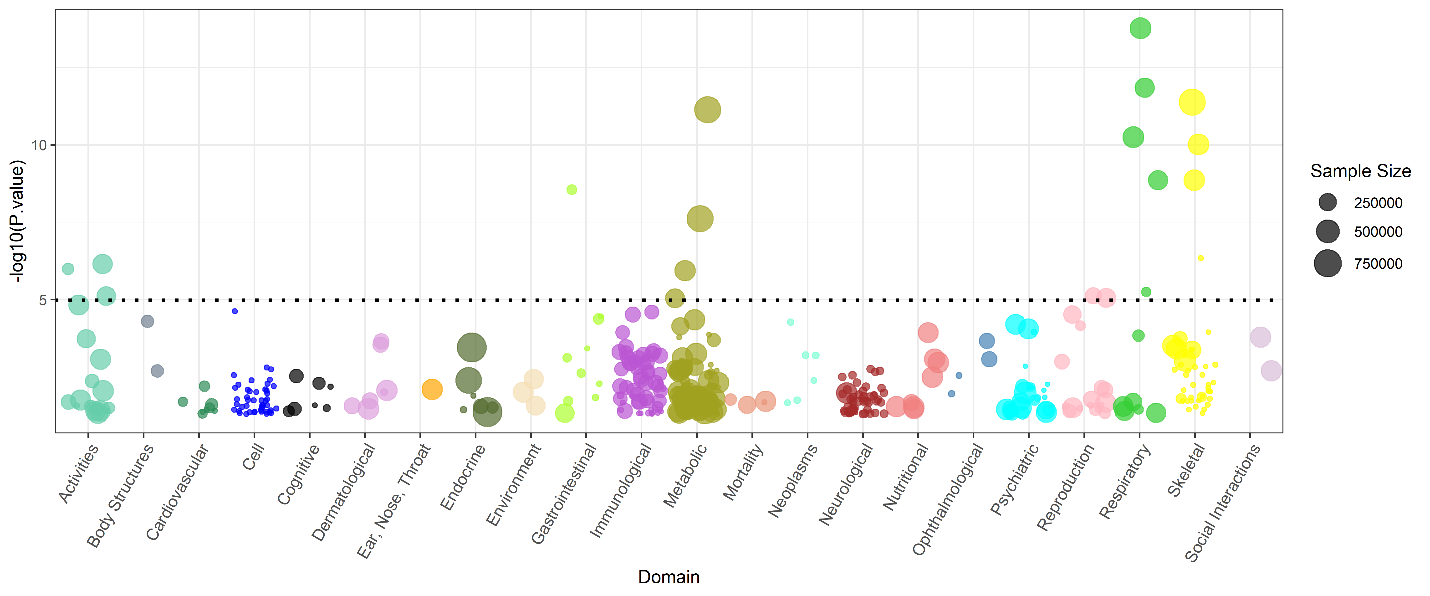


Figure S31: NR5A2. Each data point presents trait associated with gene as mined from the GWAS Atlas, traits are grouped in domains (x-axis) and size of the data point represents the sample size (legend on right) of the study for which the association statistic was reported. The y-axis shows -log10(p-value) of the gene with the respective trait. The dotted line presents Bonferroni significance line (1e-5) correcting for the traits present in the GWASAtlas.


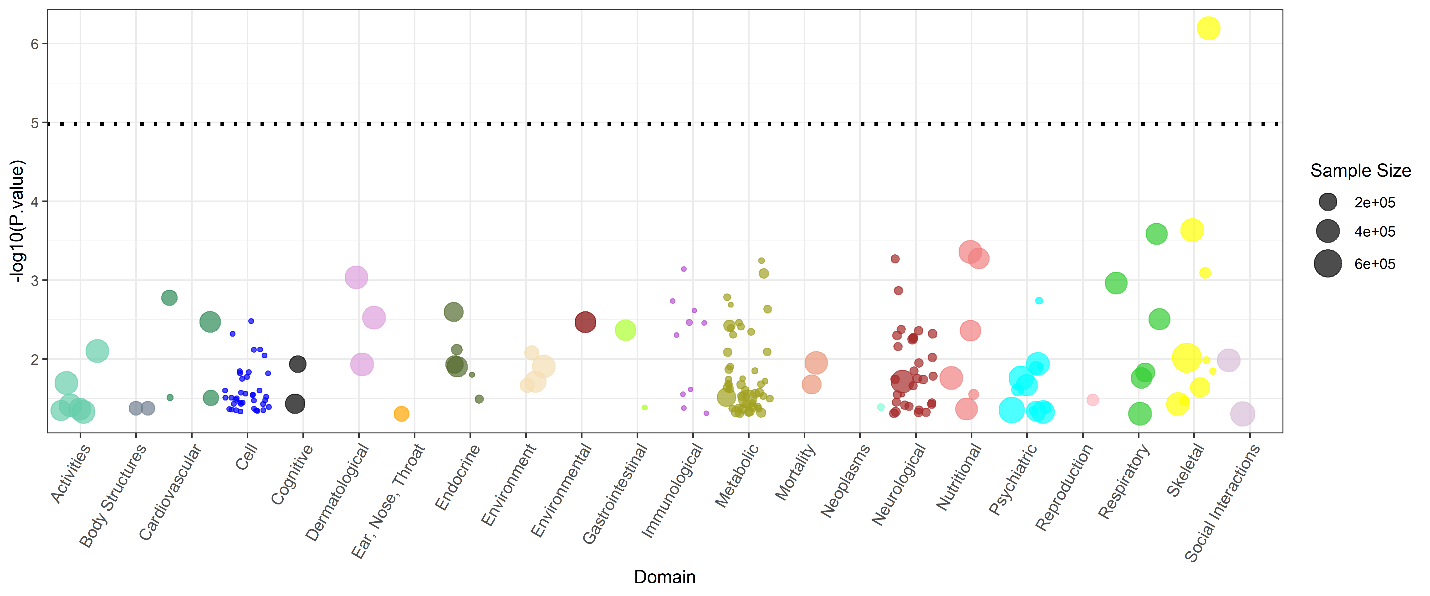


Figure S32: NTS. Each data point presents trait associated with gene as mined from the GWAS Atlas, traits are grouped in domains (x-axis) and size of the data point represents the sample size (legend on right) of the study for which the association statistic was reported. The y-axis shows -log10(p-value) of the gene with the respective trait. The dotted line presents Bonferroni significance line (1e-5) correcting for the traits present in the GWASAtlas.


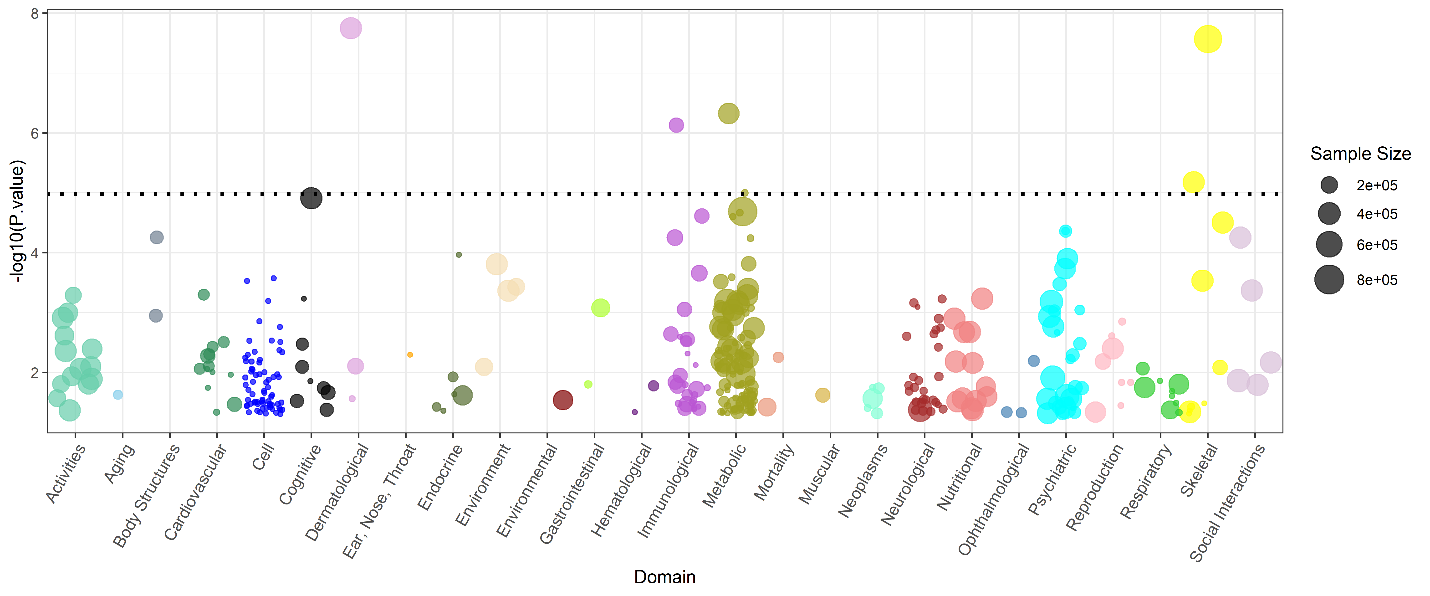


Figure S33: PDE9A. Each data point presents trait associated with gene as mined from the GWAS Atlas, traits are grouped in domains (x-axis) and size of the data point represents the sample size (legend on right) of the study for which the association statistic was reported. The y-axis shows -log10(p-value) of the gene with the respective trait. The dotted line presents Bonferroni significance line (1e-5) correcting for the traits present in the GWASAtlas.


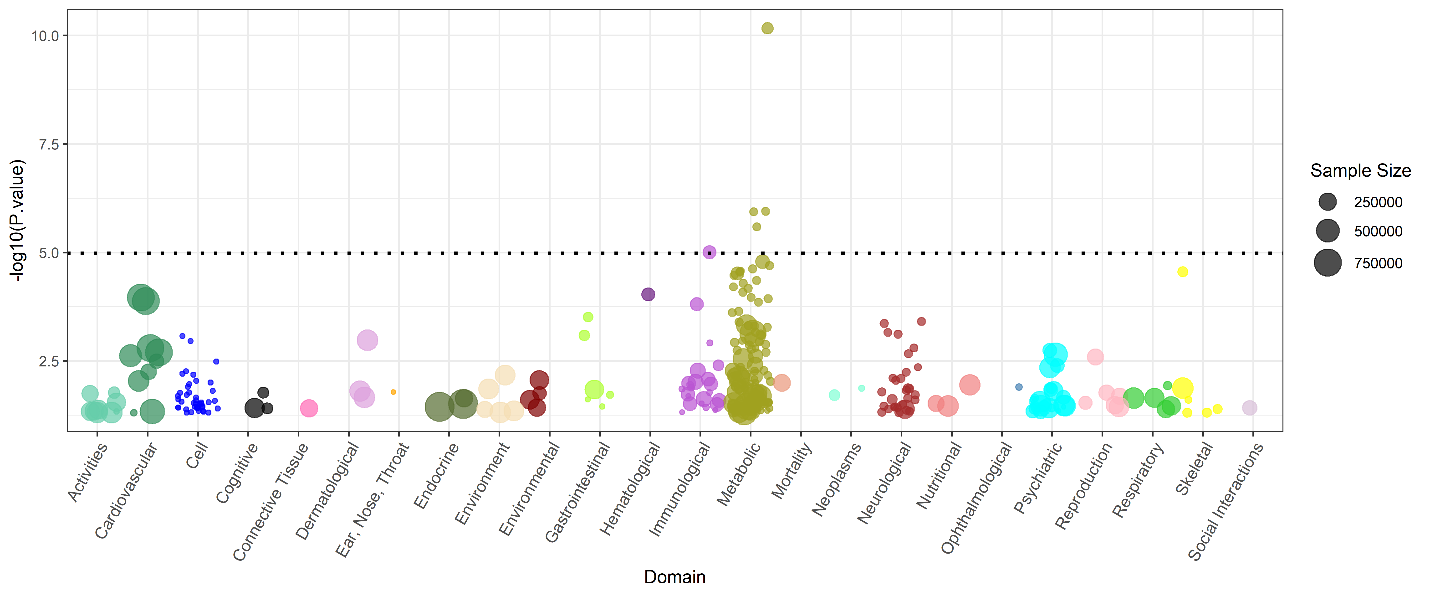


Figure S34: PLA2G1B. Each data point presents trait associated with gene as mined from the GWAS Atlas, traits are grouped in domains (x-axis) and size of the data point represents the sample size (legend on right) of the study for which the association statistic was reported. The y-axis shows -log10(p-value) of the gene with the respective trait. The dotted line presents Bonferroni significance line (1e-5) correcting for the traits present in the GWASAtlas.


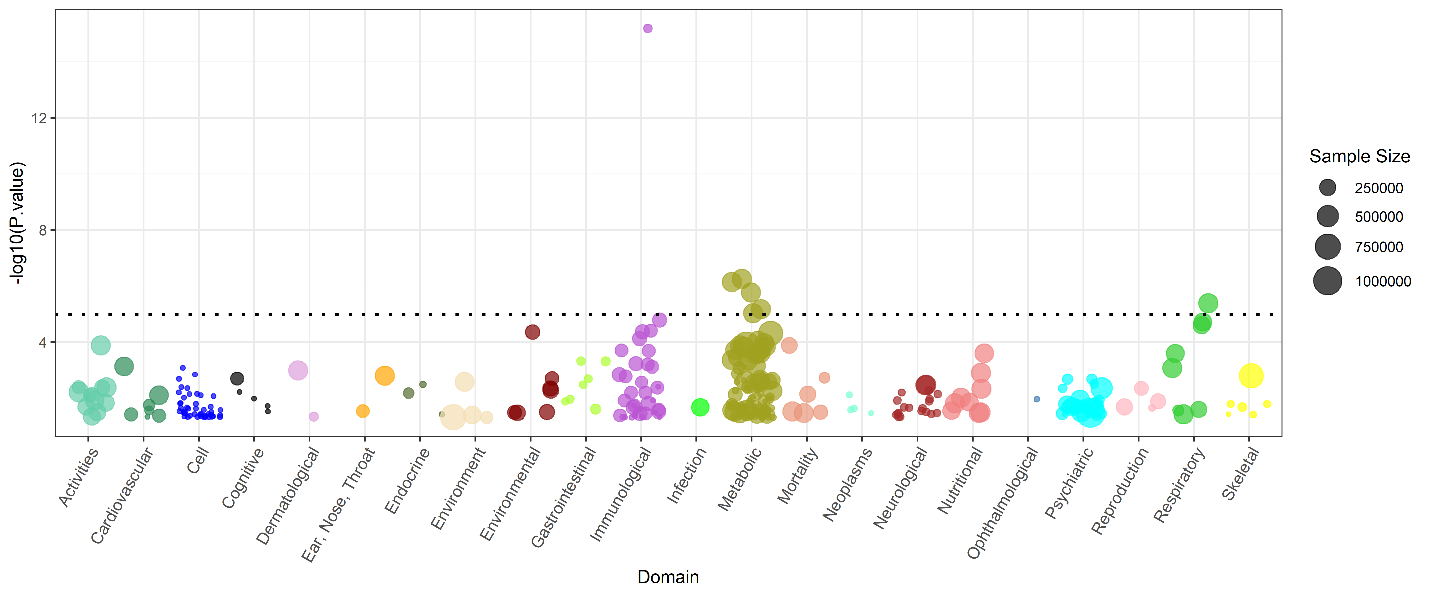


Figure S35: POU2F1. Each data point presents trait associated with gene as mined from the GWAS Atlas, traits are grouped in domains (x-axis) and size of the data point represents the sample size (legend on right) of the study for which the association statistic was reported. The y-axis shows -log10(p-value) of the gene with the respective trait. The dotted line presents Bonferroni significance line (1e-5) correcting for the traits present in the GWASAtlas.


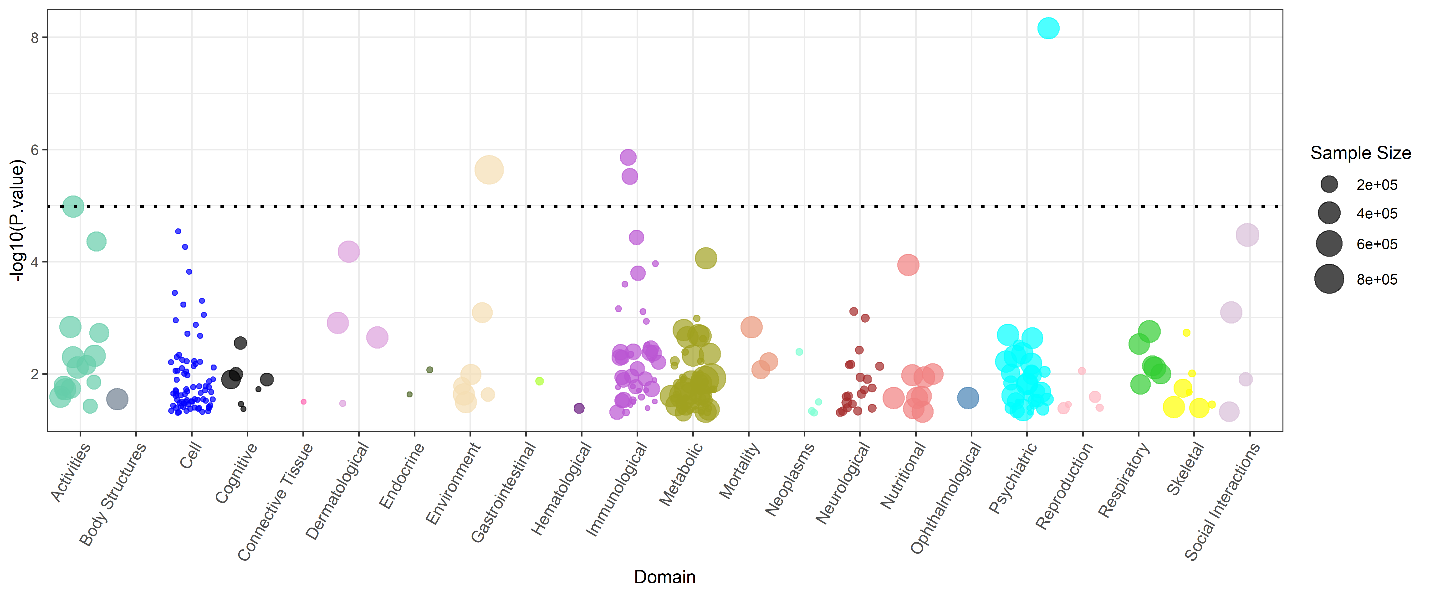


Figure S36: PRCP. Each data point presents trait associated with gene as mined from the GWAS Atlas, traits are grouped in domains (x-axis) and size of the data point represents the sample size (legend on right) of the study for which the association statistic was reported. The y-axis shows -log10(p-value) of the gene with the respective trait. The dotted line presents Bonferroni significance line (1e-5) correcting for the traits present in the GWASAtlas.


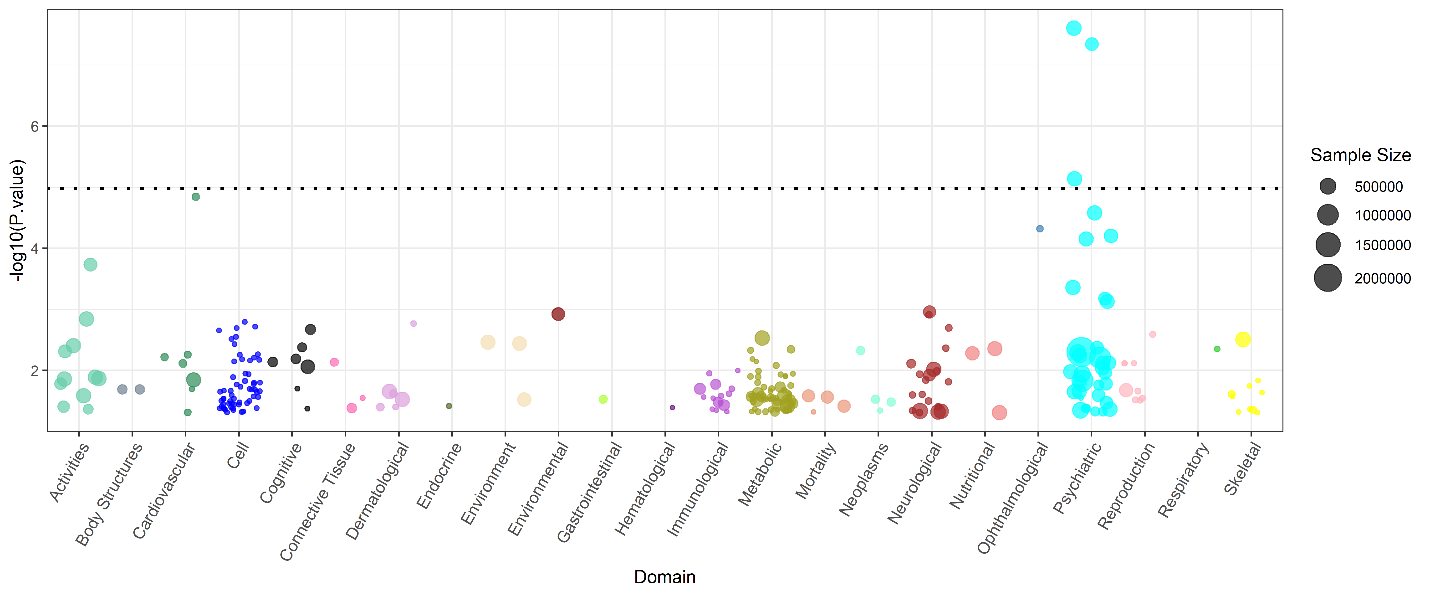


Figure S37:RASEF. Each data point presents trait associated with gene as mined from the GWAS Atlas, traits are grouped in domains (x-axis) and size of the data point represents the sample size (legend on right) of the study for which the association statistic was reported. The y-axis shows -log10(p-value) of the gene with the respective trait. The dotted line presents Bonferroni significance line (1e-5) correcting for the traits present in the GWASAtlas.


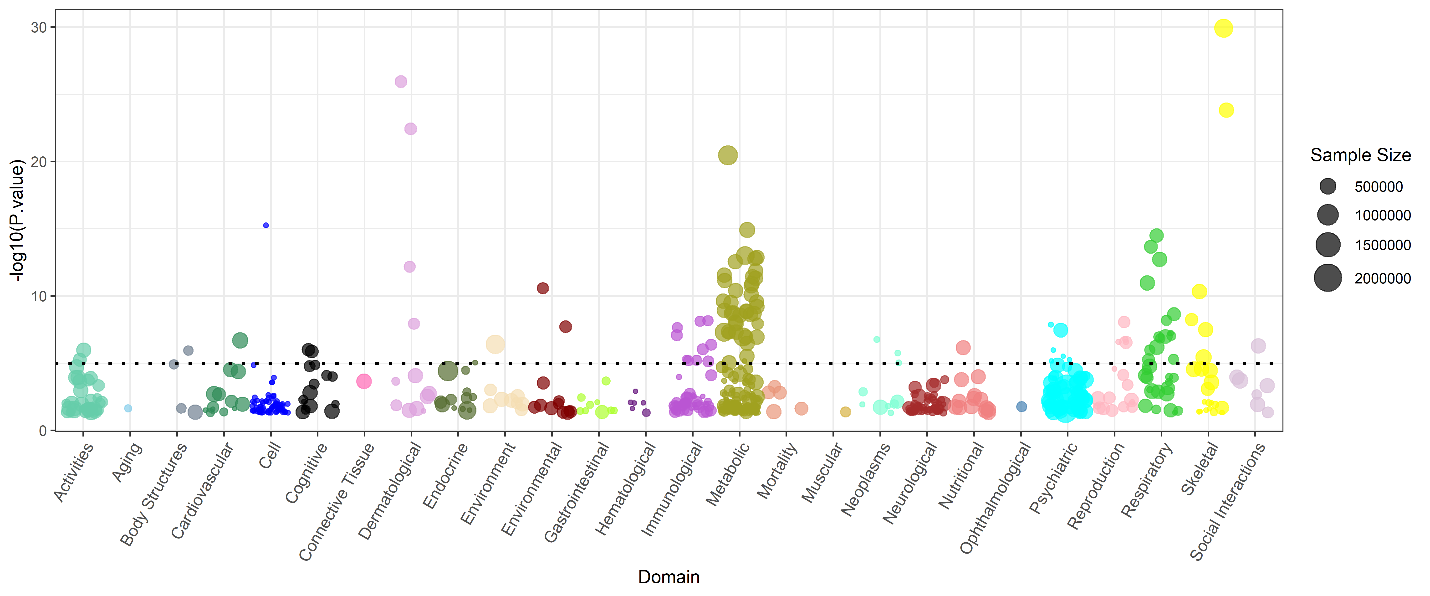


Figure S38: RORA. Each data point presents trait associated with gene as mined from the GWAS Atlas, traits are grouped in domains (x-axis) and size of the data point represents the sample size (legend on right) of the study for which the association statistic was reported. The y-axis shows -log10(p-value) of the gene with the respective trait. The dotted line presents Bonferroni significance line (1e-5) correcting for the traits present in the GWASAtlas.


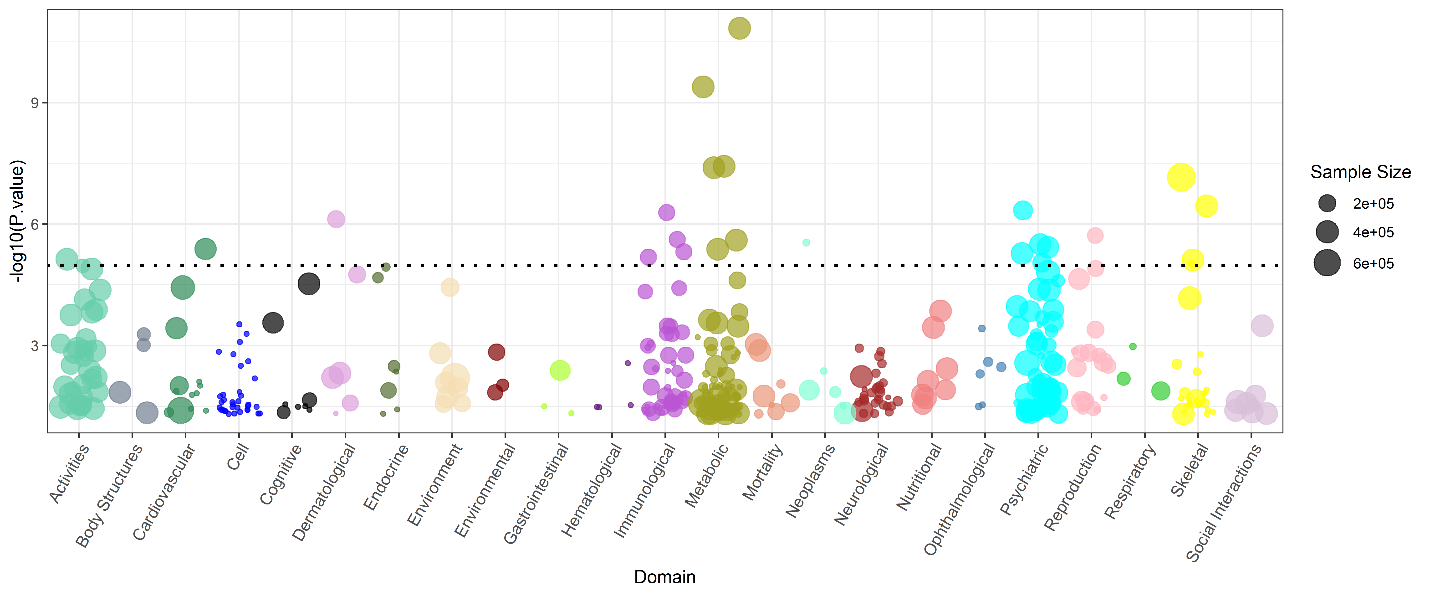


Figure S39: RORB. Each data point presents trait associated with gene as mined from the GWAS Atlas, traits are grouped in domains (x-axis) and size of the data point represents the sample size (legend on right) of the study for which the association statistic was reported. The y-axis shows -log10(p-value) of the gene with the respective trait. The dotted line presents Bonferroni significance line (1e-5) correcting for the traits present in the GWASAtlas.


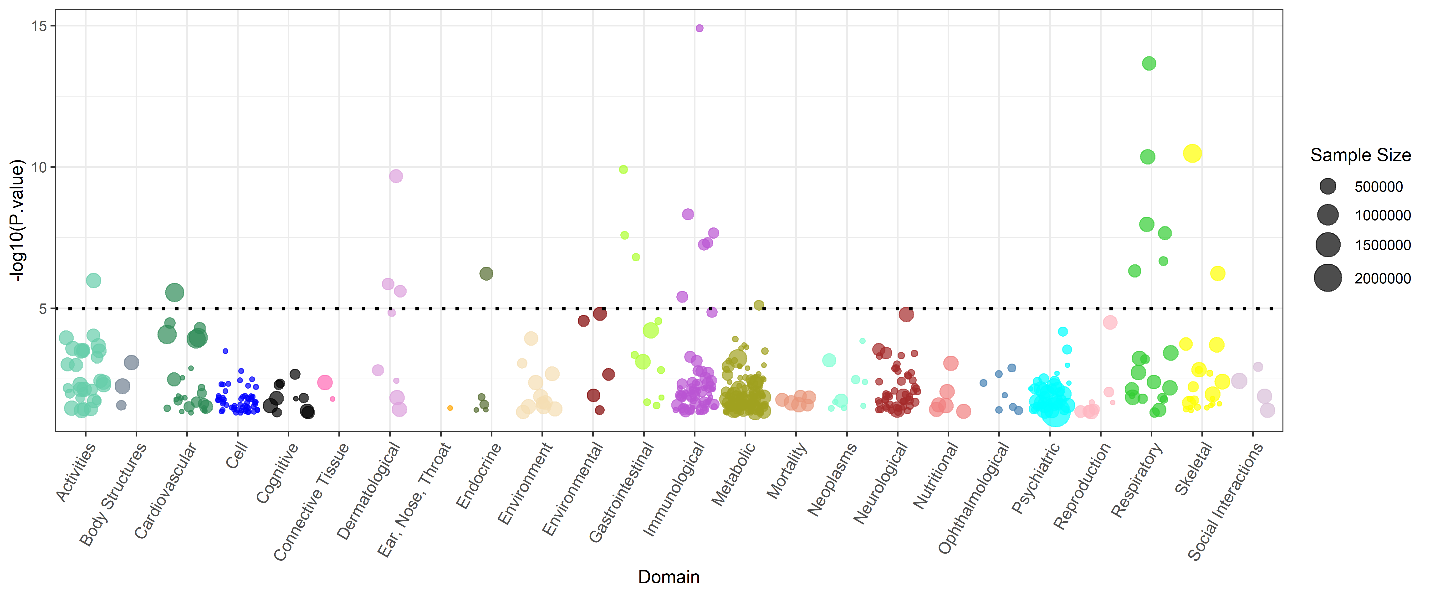


Figure S40: RORC. Each data point presents trait associated with gene as mined from the GWAS Atlas, traits are grouped in domains (x-axis) and size of the data point represents the sample size (legend on right) of the study for which the association statistic was reported. The y-axis shows -log10(p-value) of the gene with the respective trait. The dotted line presents Bonferroni significance line (1e-5) correcting for the traits present in the GWASAtlas.


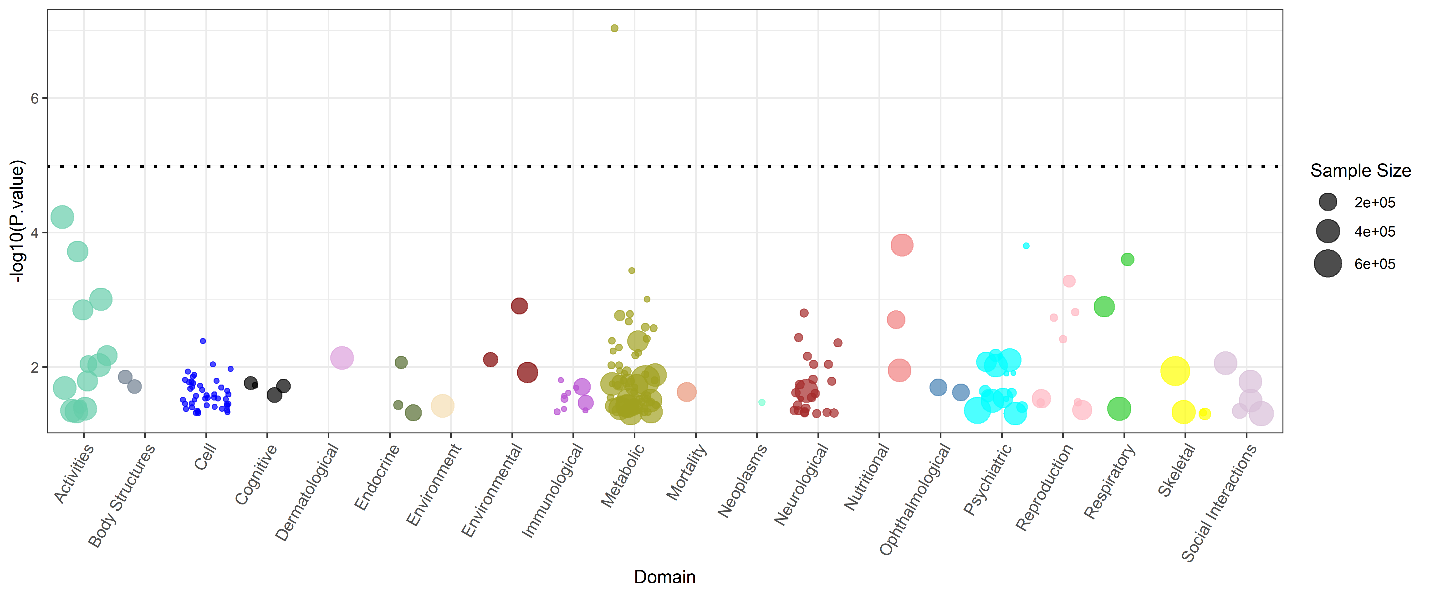


Figure S41:SI. Each data point presents trait associated with gene as mined from the GWAS Atlas, traits are grouped in domains (x-axis) and size of the data point represents the sample size (legend on right) of the study for which the association statistic was reported. The y-axis shows -log10(p-value) of the gene with the respective trait. The dotted line presents Bonferroni significance line (1e-5) correcting for the traits present in the GWASAtlas.


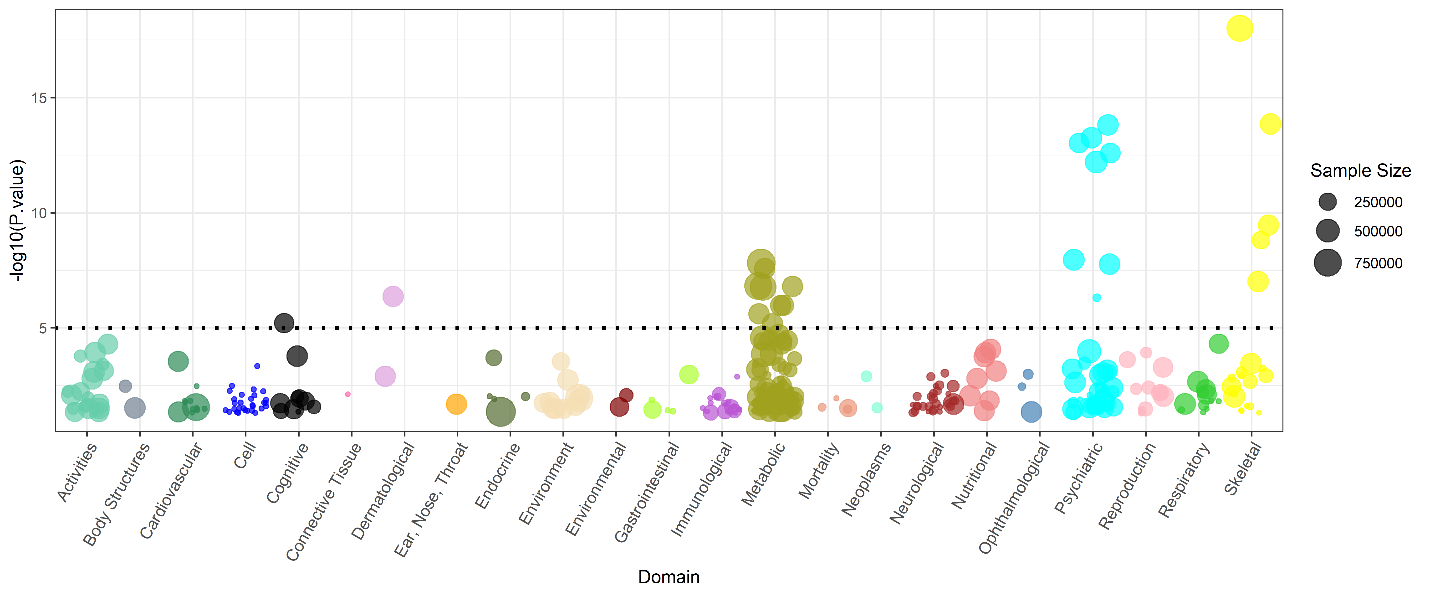


Figure S42: SLC3A1. Each data point presents trait associated with gene as mined from the GWAS Atlas, traits are grouped in domains (x-axis) and size of the data point represents the sample size (legend on right) of the study for which the association statistic was reported. The y-axis shows -log10(p-value) of the gene with the respective trait. The dotted line presents Bonferroni significance line (1e-5) correcting for the traits present in the GWASAtlas.


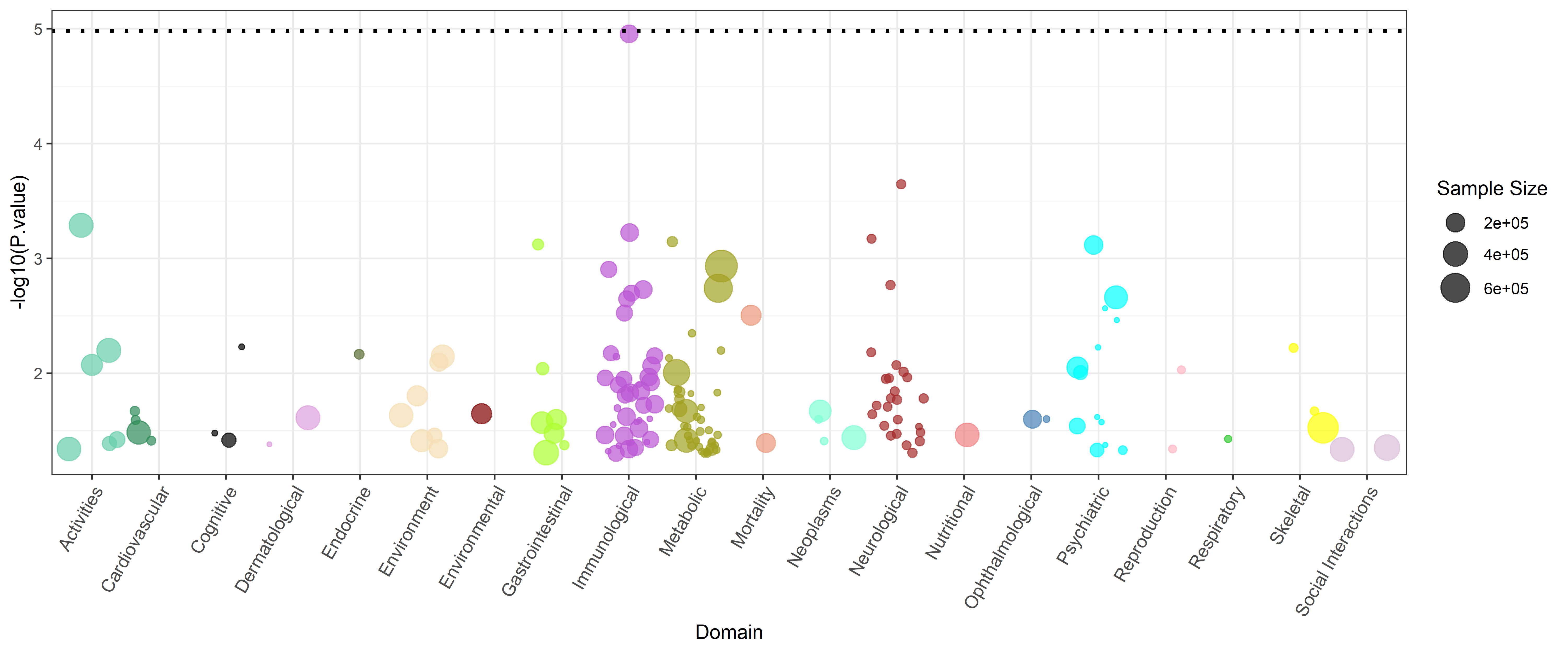


Figure S43: SLC6A19. Each data point presents trait associated with gene as mined from the GWAS Atlas, traits are grouped in domains (x-axis) and size of the data point represents the sample size (legend on right) of the study for which the association statistic was reported. The y-axis shows -log10(p-value) of the gene with the respective trait. The dotted line presents Bonferroni significance line (1e-5) correcting for the traits present in the GWASAtlas.


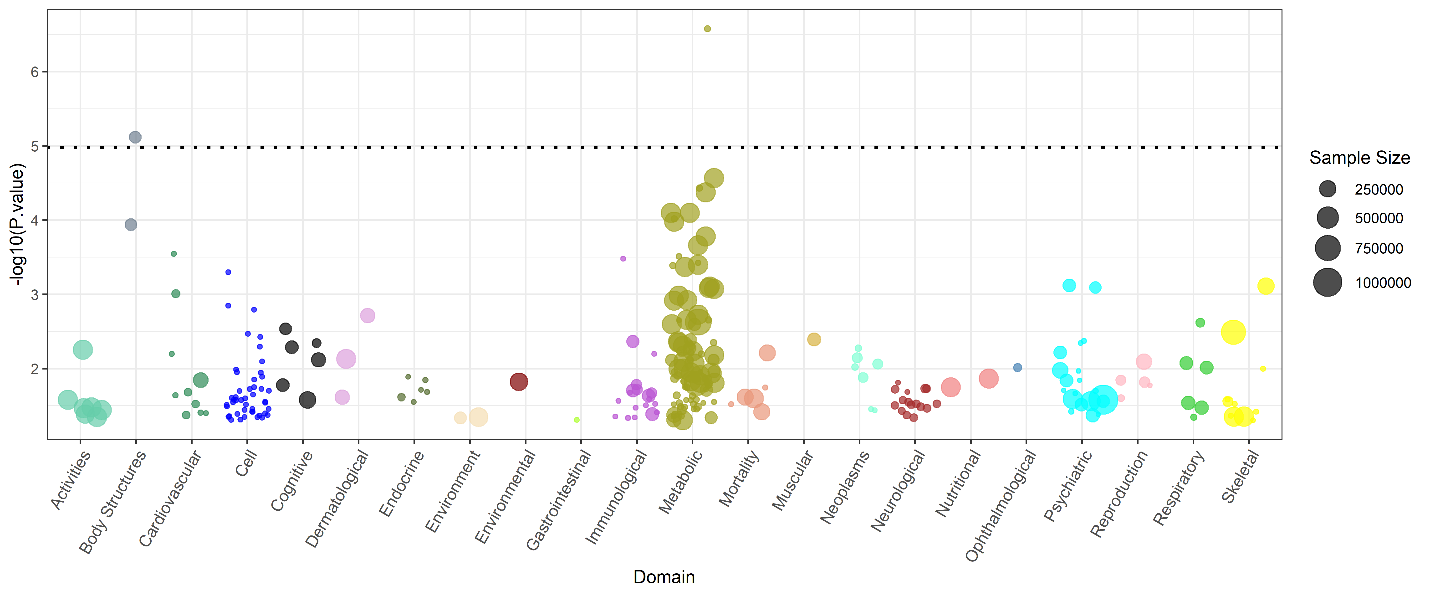


Figure S44: SLC10A2. Each data point presents trait associated with gene as mined from the GWAS Atlas, traits are grouped in domains (x-axis) and size of the data point represents the sample size (legend on right) of the study for which the association statistic was reported. The y-axis shows -log10(p-value) of the gene with the respective trait. The dotted line presents Bonferroni significance line (1e-5) correcting for the traits present in the GWASAtlas.


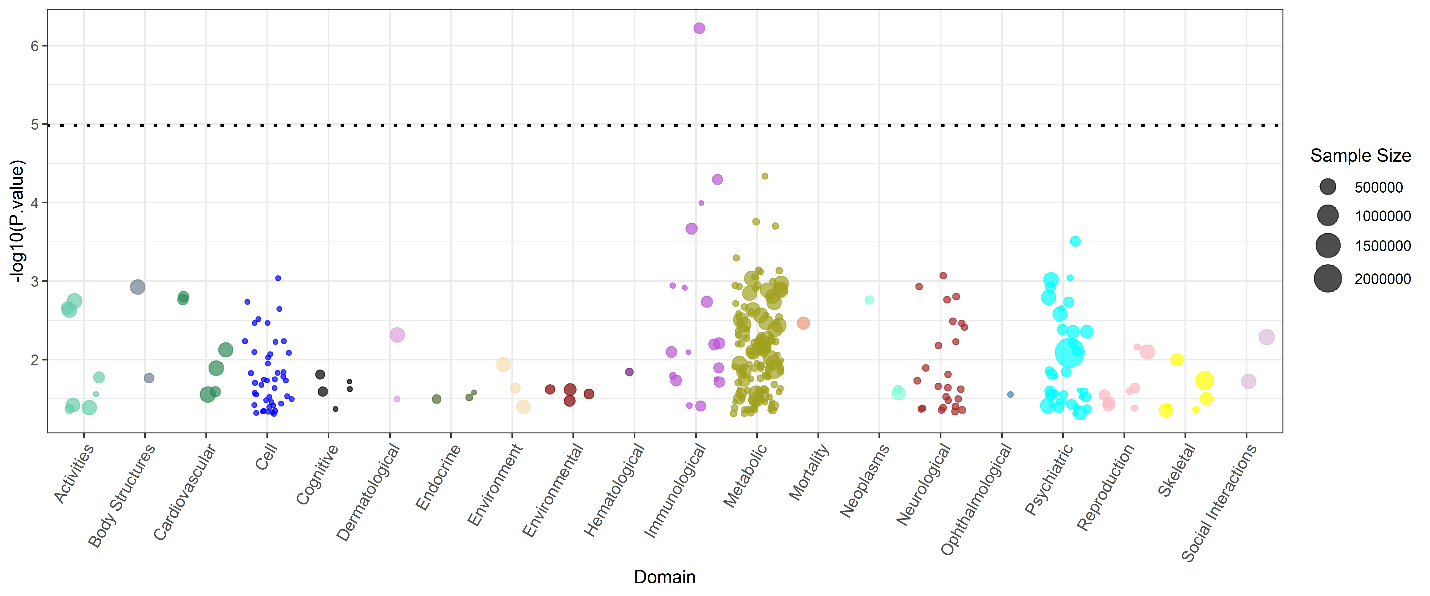


Figure S45: SLC12A6. Each data point presents trait associated with gene as mined from the GWAS Atlas, traits are grouped in domains (x-axis) and size of the data point represents the sample size (legend on right) of the study for which the association statistic was reported. The y-axis shows -log10(p-value) of the gene with the respective trait. The dotted line presents Bonferroni significance line (1e-5) correcting for the traits present in the GWASAtlas.


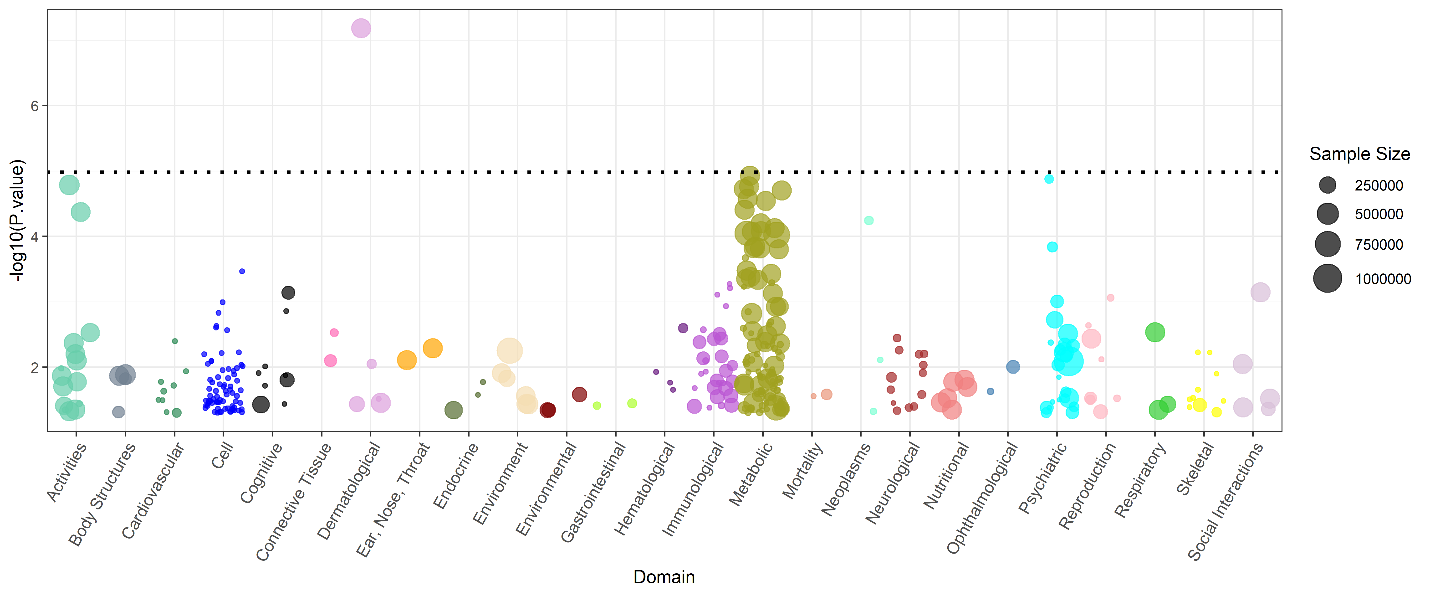


Figure S46: SLC37A1. Each data point presents trait associated with gene as mined from the GWAS Atlas, traits are grouped in domains (x-axis) and size of the data point represents the sample size (legend on right) of the study for which the association statistic was reported. The y-axis shows -log10(p-value) of the gene with the respective trait. The dotted line presents Bonferroni significance line (1e-5) correcting for the traits present in the GWASAtlas.


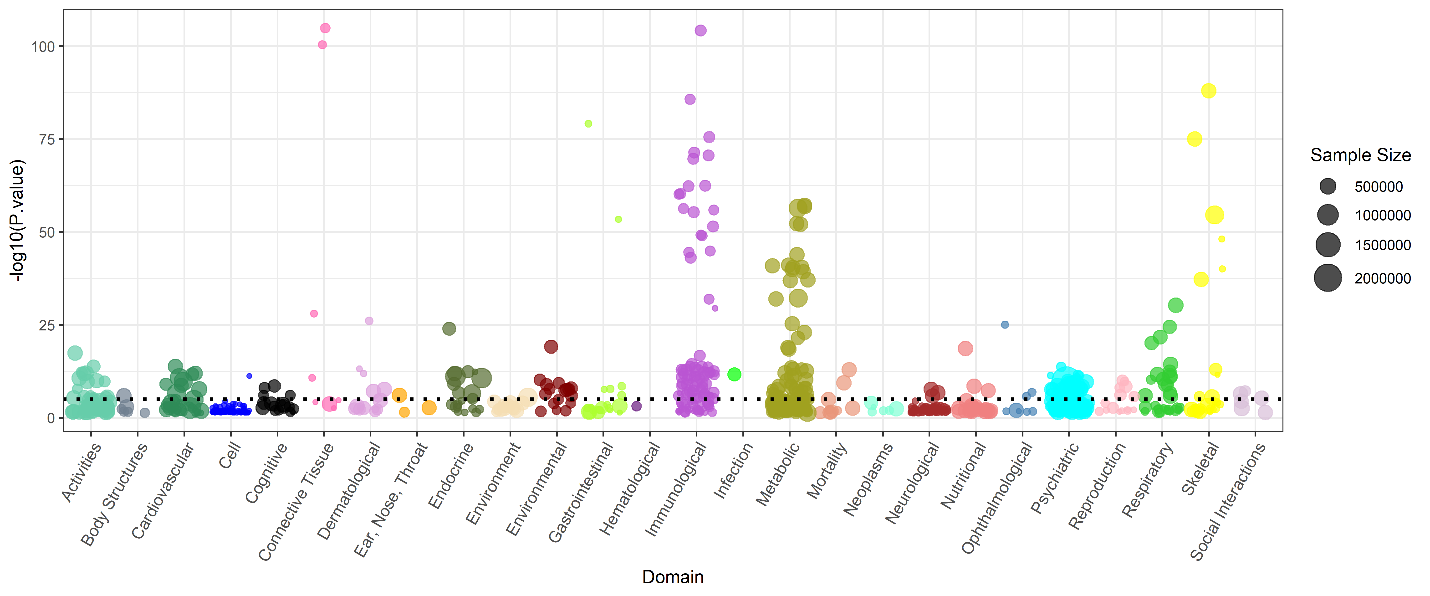


Figure S47: SLC44A4. Each data point presents trait associated with gene as mined from the GWAS Atlas, traits are grouped in domains (x-axis) and size of the data point represents the sample size (legend on right) of the study for which the association statistic was reported. The y-axis shows -log10(p-value) of the gene with the respective trait. The dotted line presents Bonferroni significance line (1e-5) correcting for the traits present in the GWASAtlas.


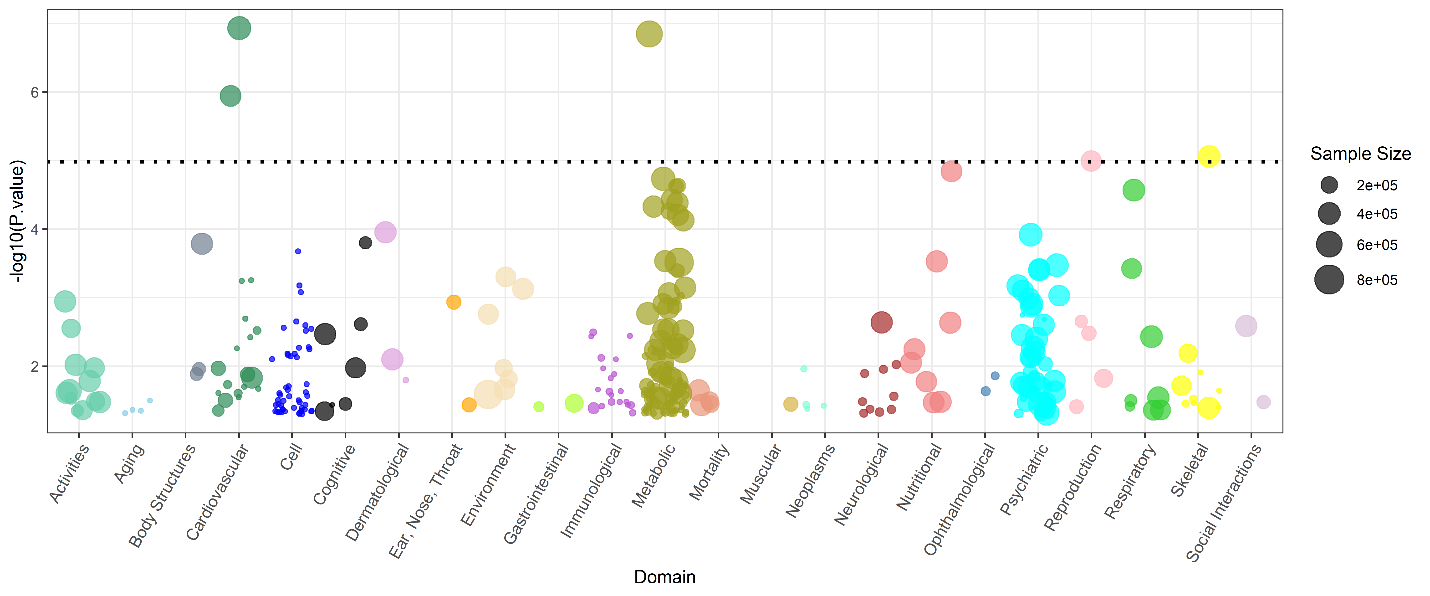


Figure S48: TINAG. Each data point presents trait associated with gene as mined from the GWAS Atlas, traits are grouped in domains (x-axis) and size of the data point represents the sample size (legend on right) of the study for which the association statistic was reported. The y-axis shows -log10(p-value) of the gene with the respective trait. The dotted line presents Bonferroni significance line (1e-5) correcting for the traits present in the GWASAtlas.


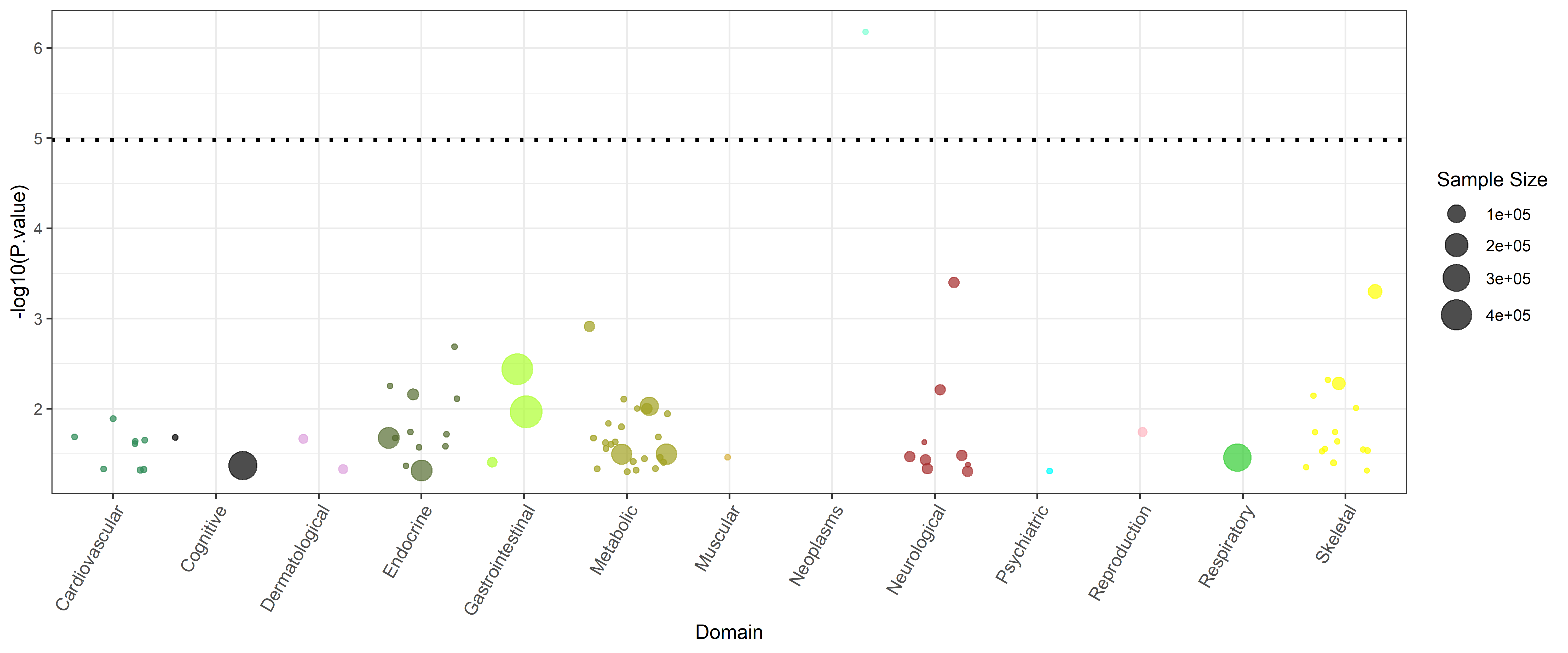


Figure S49: TMEM27. Each data point presents trait associated with gene as mined from the GWAS Atlas, traits are grouped in domains (x-axis) and size of the data point represents the sample size (legend on right) of the study for which the association statistic was reported. The y-axis shows -log10(p-value) of the gene with the respective trait. The dotted line presents Bonferroni significance line (1e-5) correcting for the traits present in the GWASAtlas.


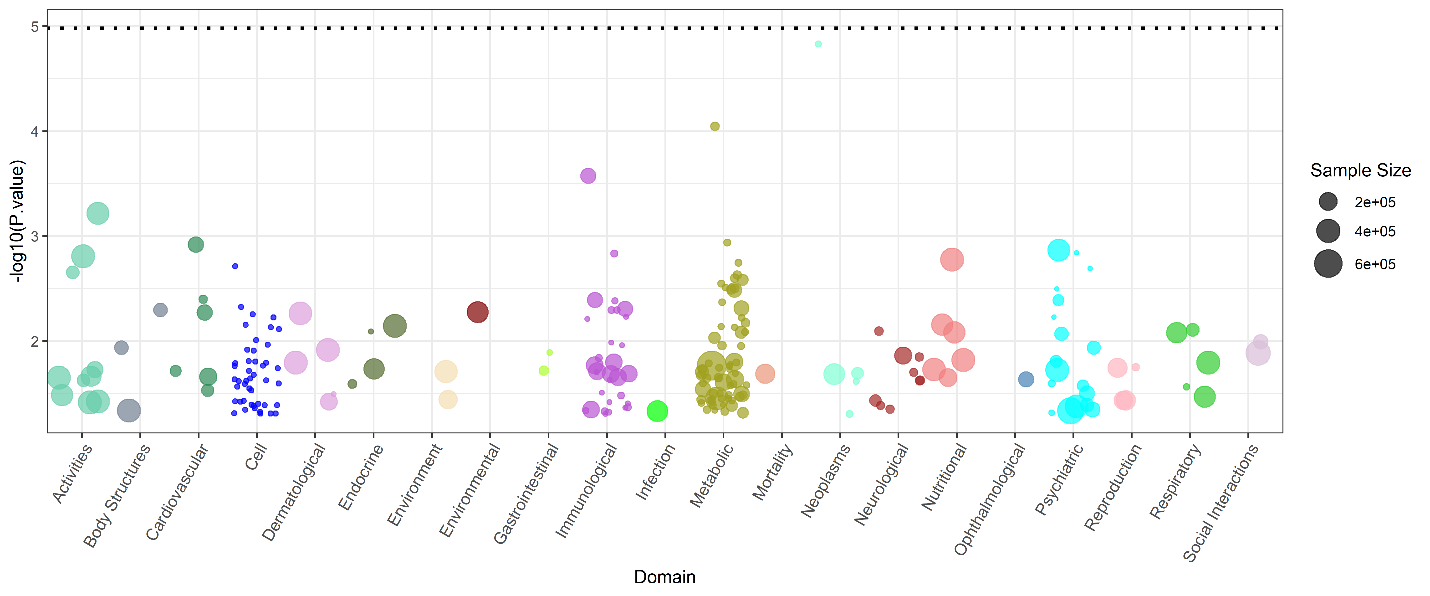


Figure S50: TMPRSS2. Each data point presents trait associated with gene as mined from the GWAS Atlas, traits are grouped in domains (x-axis) and size of the data point represents the sample size (legend on right) of the study for which the association statistic was reported. The y-axis shows -log10(p-value) of the gene with the respective trait. The dotted line presents Bonferroni significance line (1e-5) correcting for the traits present in the GWASAtlas.


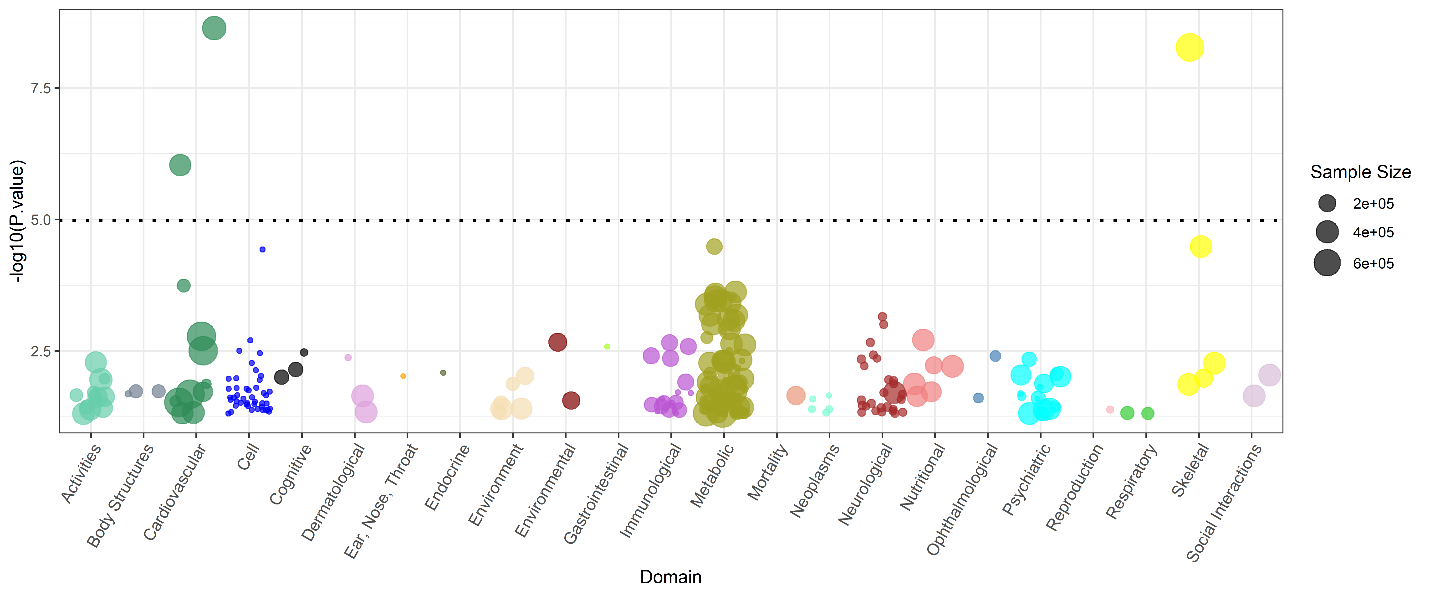


Figure S51: TRPM4. Each data point presents trait associated with gene as mined from the GWAS Atlas, traits are grouped in domains (x-axis) and size of the data point represents the sample size (legend on right) of the study for which the association statistic was reported. The y-axis shows -log10(p-value) of the gene with the respective trait. The dotted line presents Bonferroni significance line (1e-5) correcting for the traits present in the GWASAtlas.


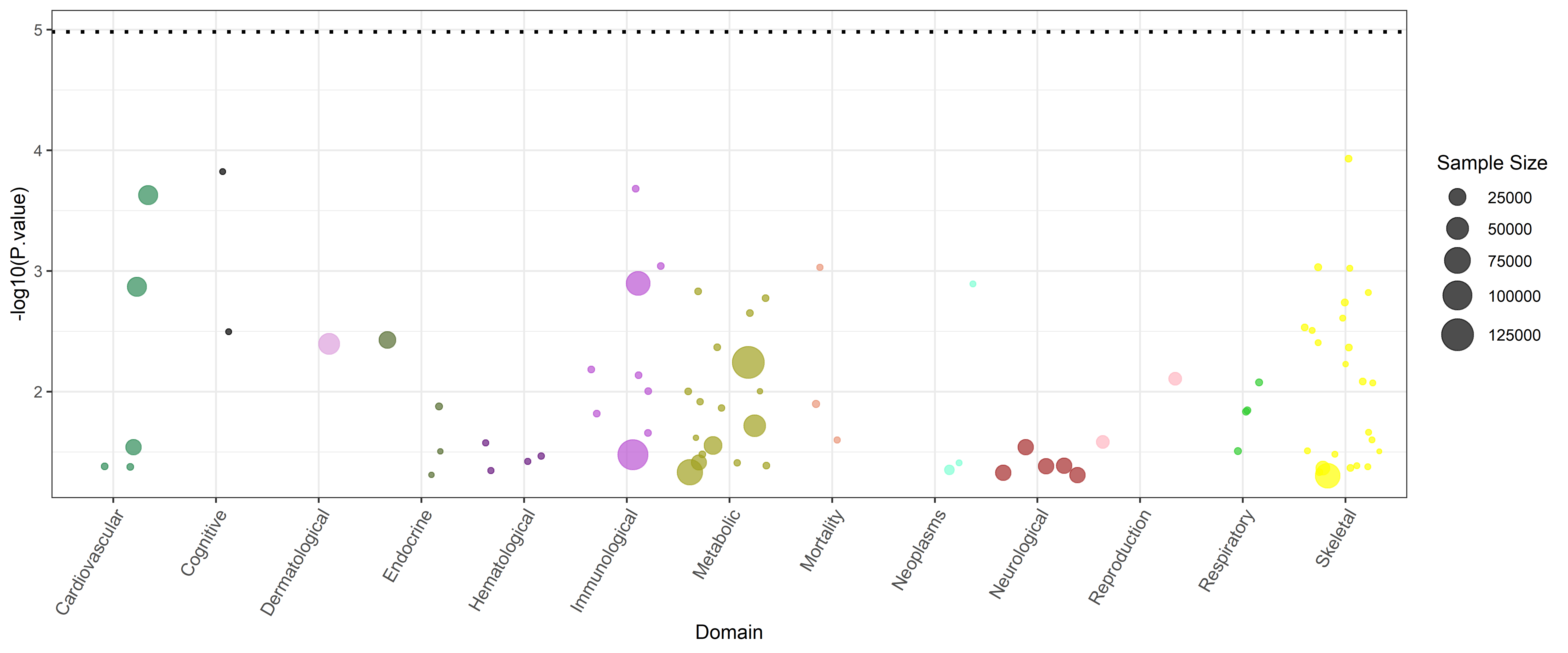


Figure S52: XPNPEP2. Each data point presents trait associated with gene as mined from the GWAS Atlas, traits are grouped in domains (x-axis) and size of the data point represents the sample size (legend on right) of the study for which the association statistic was reported. The y-axis shows -log10(p-value) of the gene with the respective trait. The dotted line presents Bonferroni significance line (1e-5) correcting for the traits present in the GWASAtlas.

# miRNA enrichment


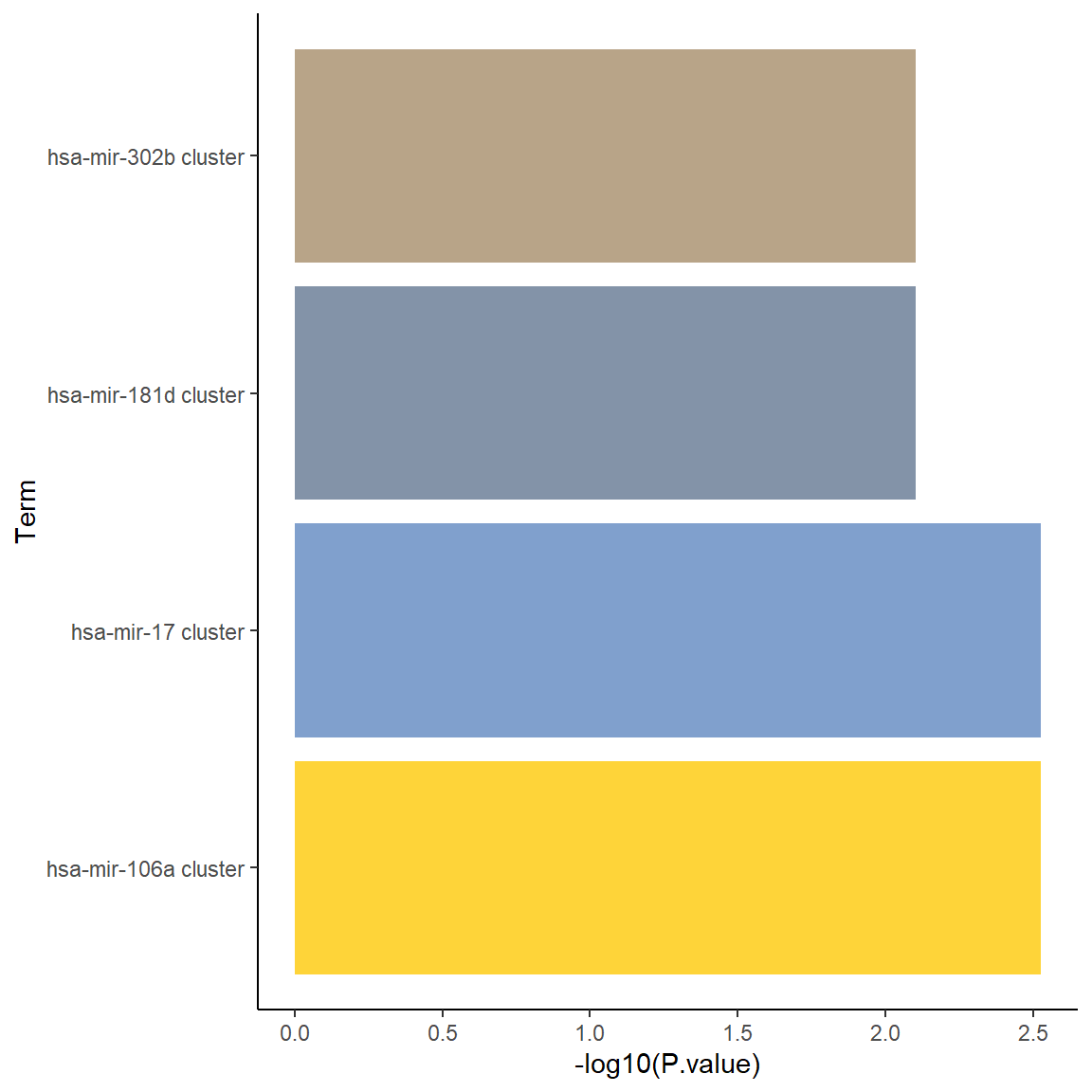


Figure S53: miRNA cluster enrichment. The x-axis shows -log10(p-value) of the miRNAs clusters (y-axis) that were FDR significant.


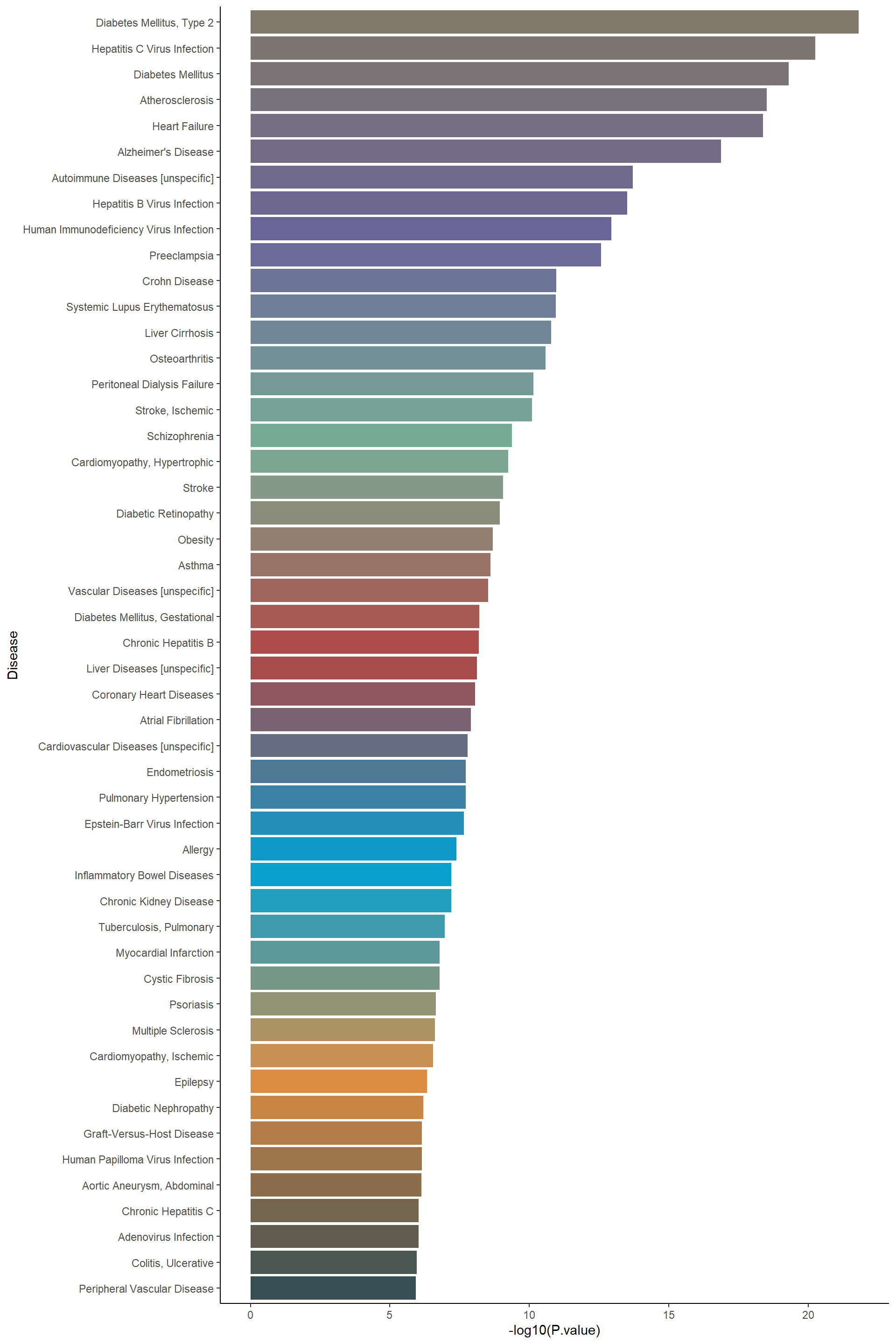


Figure S54: miRNA disease enrichment. The x-axis shows -log10(p-value) of the diseases (y-axis) that were FDR significant.

# Neanderthal local ancestry assessment for the SNPs in the ACE2 gene network


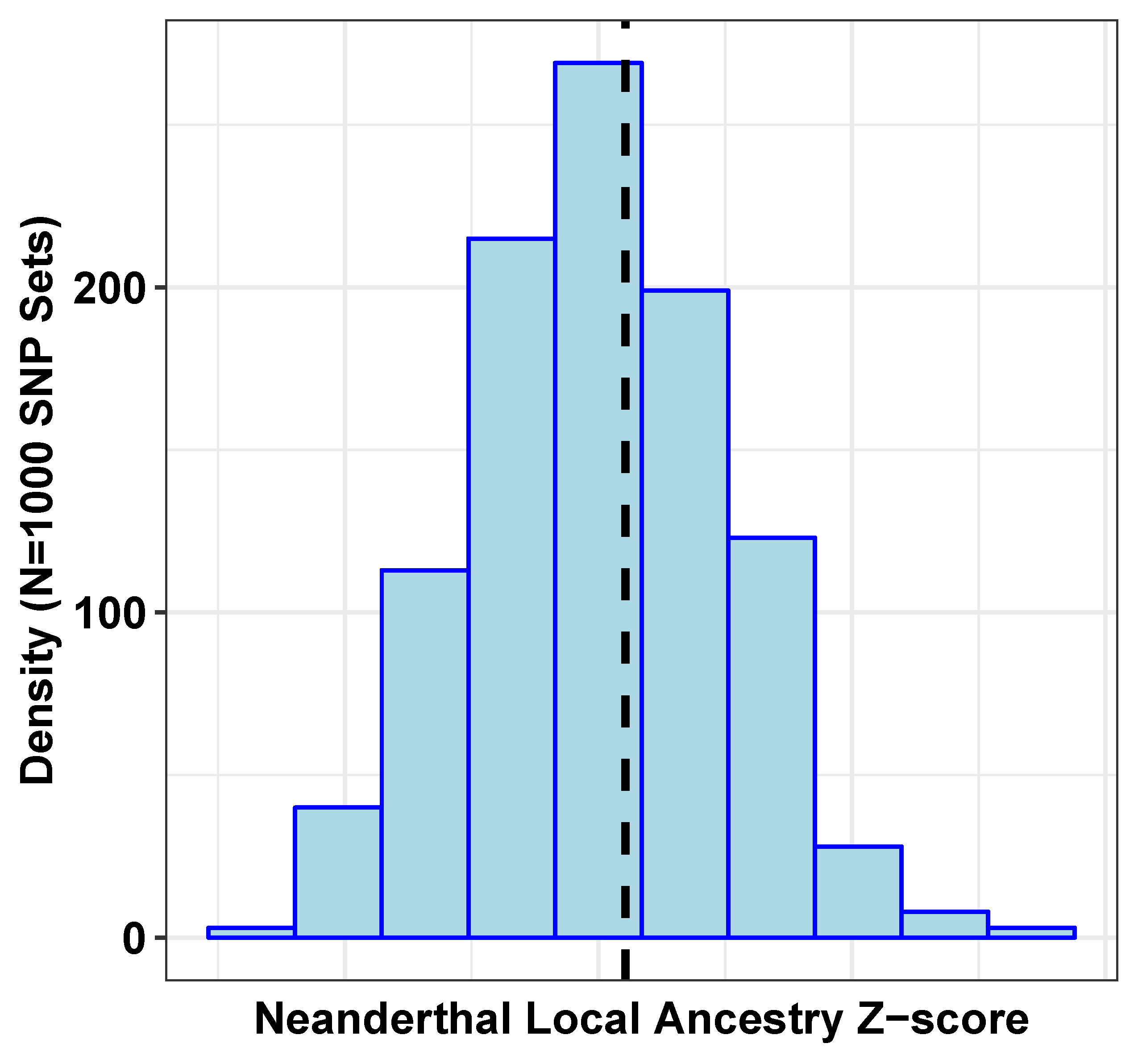


Figure S55: Neanderthal local ancestry of SNPs from the ACE2-network. The histogram showing mean probability of ACE2-network SNPs (black dashed line) against randomly selected SNPs with similar genomic features (blue bars). The Z-score of local ancestry is shown x-axis and distribution of SNP sets on y-axis.

# ACE2 network SNPs in six phenotypes of COVID-19


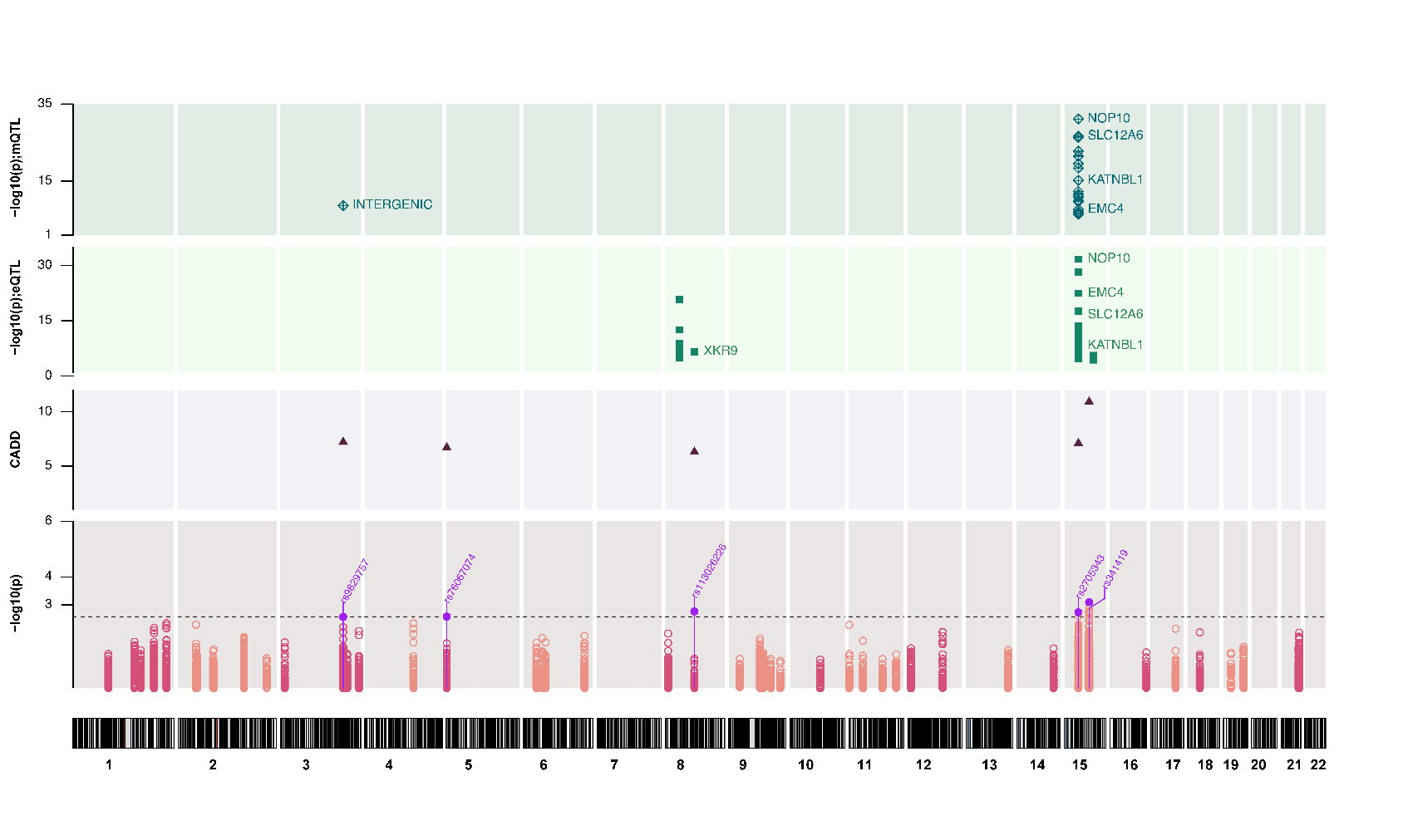


Figure S56: A2_V2 (very severe respiratory confirmed COVID-19 cases [N=536] vs. population[N=329391]). The bottom panel shows SNPs from the ACE2-gene network and SNPs highlighted in purple are LD-independent significant SNPs based on the number of LD-independent (r2<0.1) and p-value (<0.01) clumping. The significant SNPs are then annotated for Combined Annotation Dependent Depletion (CADD) scores to signify their pathogenicity (defined as score of >=10). The SNPs are also annotated for gene expression (eQTL) and methylation (mQTL) associations. The genes whose expression is associated is labelled next to the data point. The x-axis shows genomic coordinate of the SNPs grouped by chromosome, also presented as chromosome maps. The y-axis shows -log10 of the p-value or the CADD score.


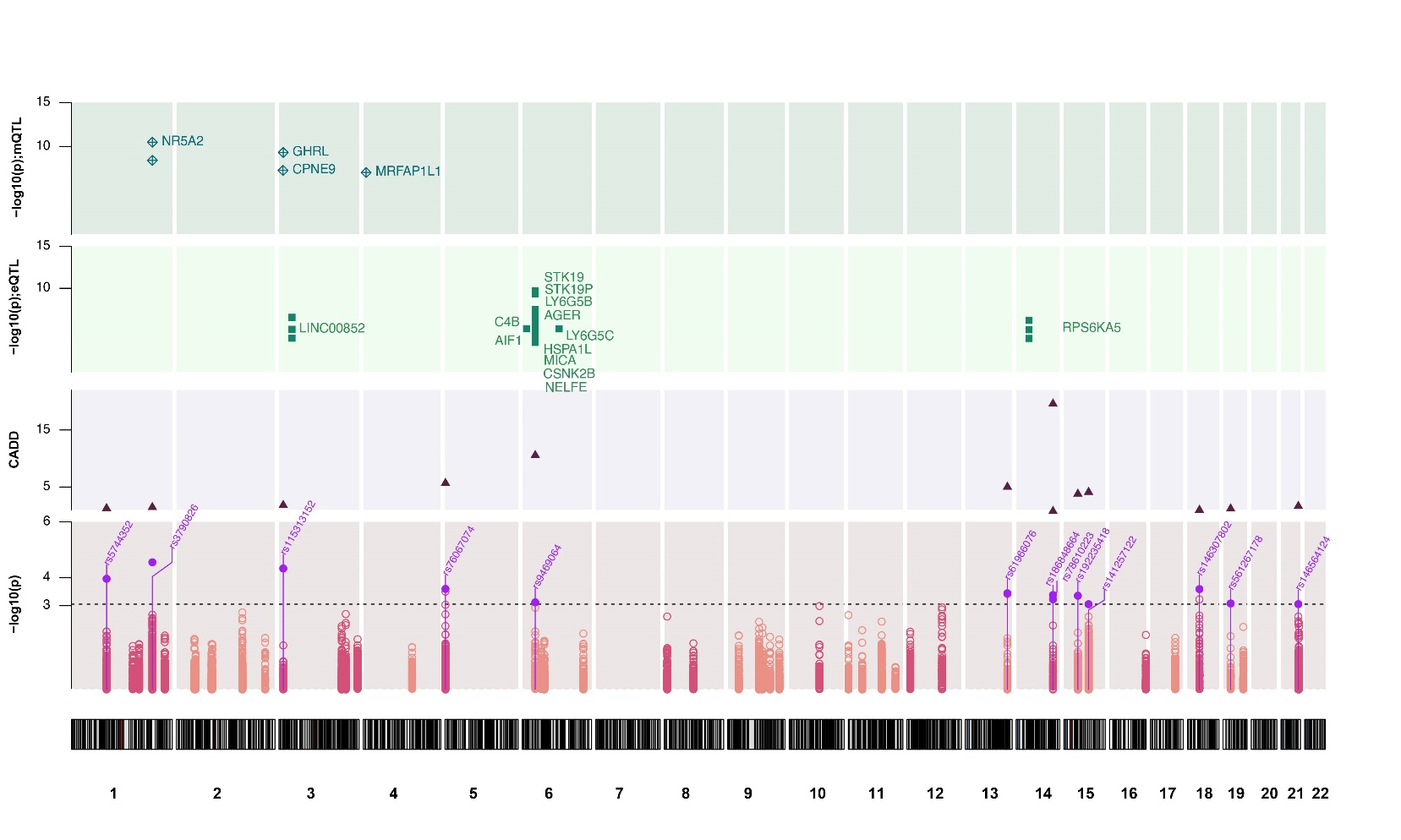


Figure S57: B1_V2(hospitalized COVID-19 cases [N=928] vs. not hospitalized COVID-19 cases [N=2028]). The bottom panel shows SNPs from the ACE2-gene network and SNPs highlighted in purple are LD-independent significant SNPs based on the number of LD-independent (r2<0.1) and p-value (<0.01) clumping. The significant SNPs are then annotated for Combined Annotation Dependent Depletion (CADD) scores to signify their pathogenicity (defined as score of >=10). The SNPs are also annotated for gene expression (eQTL) and methylation (mQTL) associations. The genes whose expression is associated is labelled next to the data point. The x-axis shows genomic coordinate of the SNPs grouped by chromosome, also presented as chromosome maps. The y-axis shows -log10 of the p-value or the CADD score.


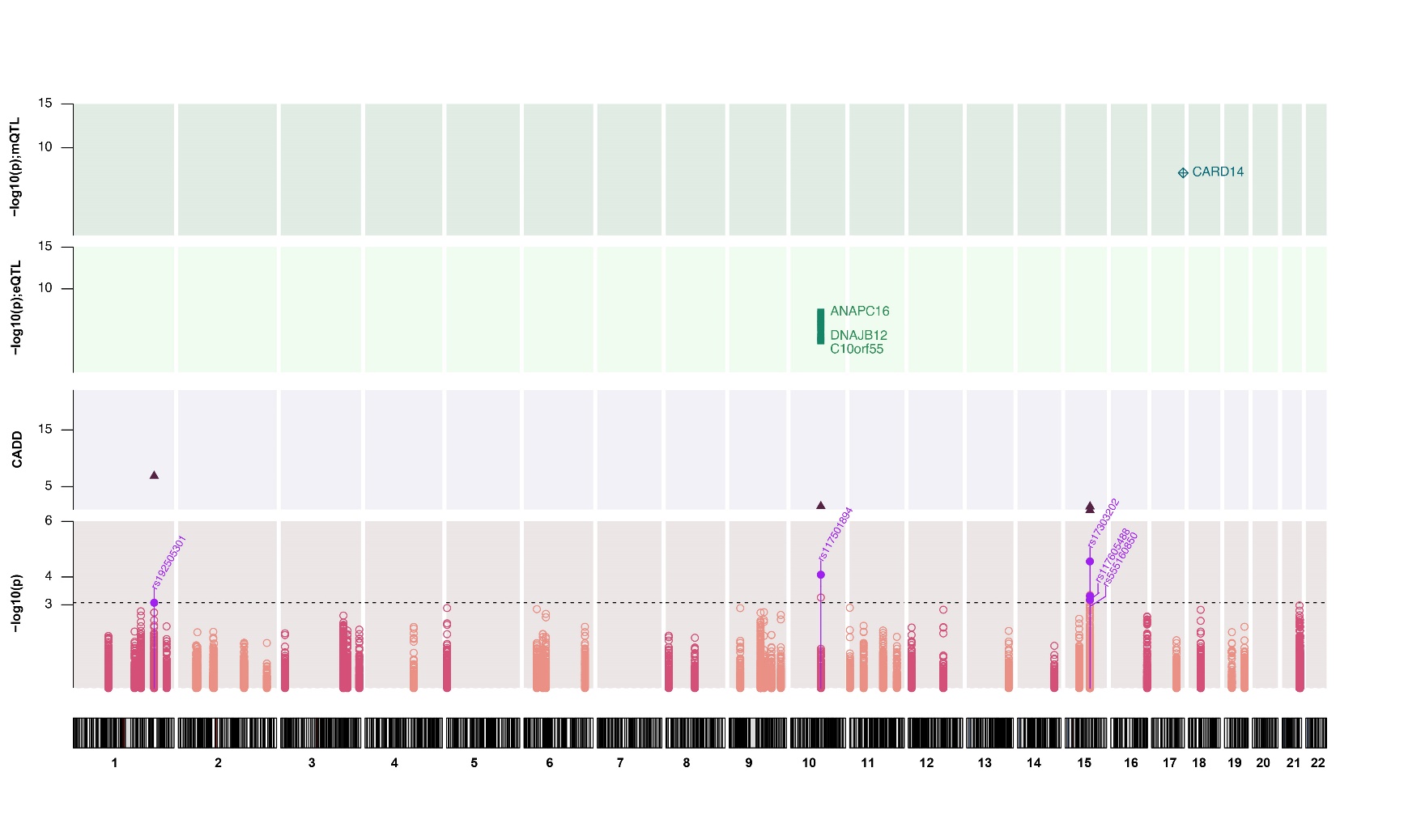


Figure S58: B2_V2(hospitalized COVID-19 cases [N=3199] vs. population [N=897488]). The bottom panel shows SNPs from the ACE2-gene network and SNPs highlighted in purple are LD-independent significant SNPs based on the number of LD-independent (r2<0.1) and p-value (<0.01) clumping. The significant SNPs are then annotated for Combined Annotation Dependent Depletion (CADD) scores to signify their pathogenicity (defined as score of >=10). The SNPs are also annotated for gene expression (eQTL) and methylation (mQTL) associations. The genes whose expression is associated is labelled next to the data point. The x-axis shows genomic coordinate of the SNPs grouped by chromosome, also presented as chromosome maps. The y-axis shows -log10 of the p-value or the CADD score.


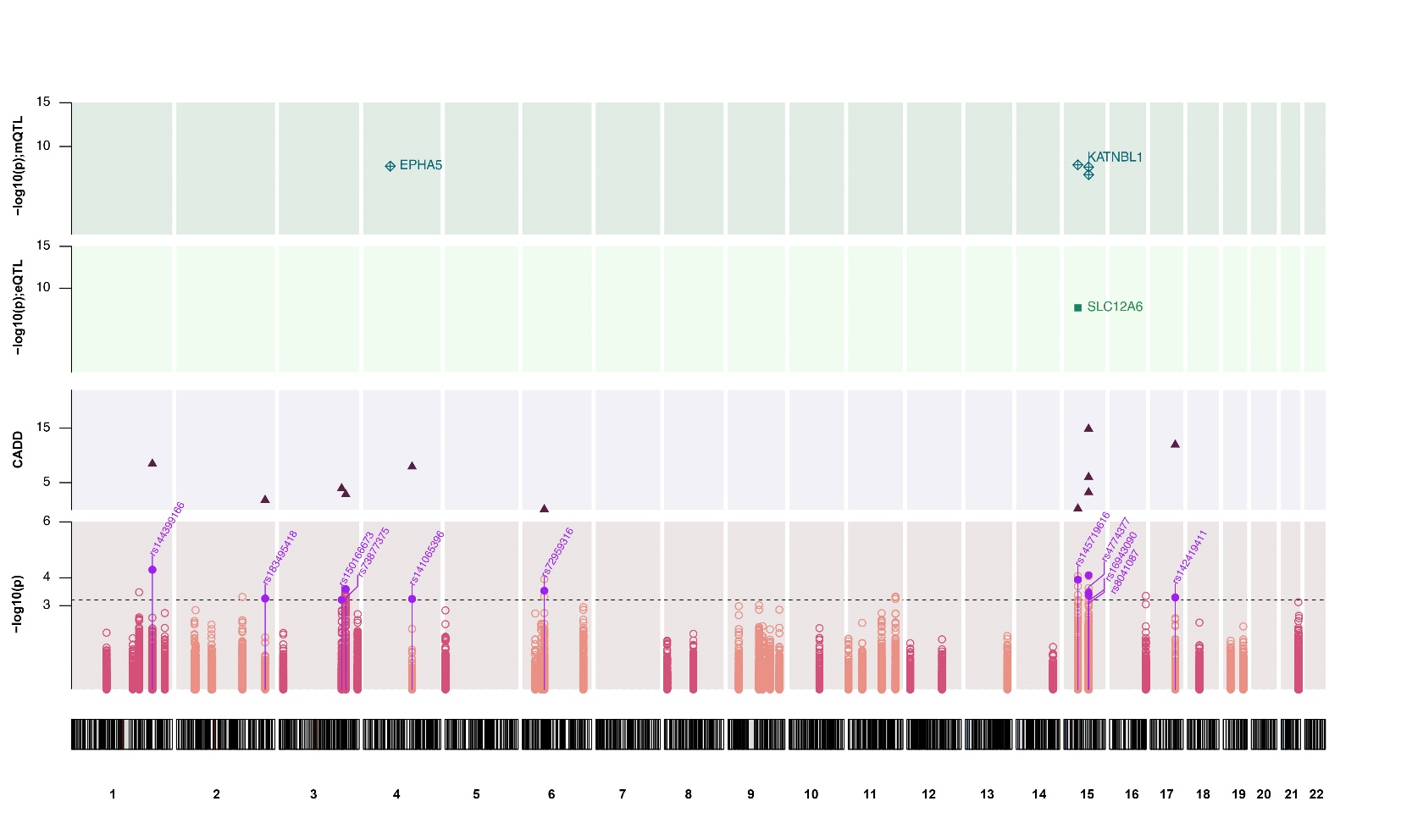


Figure S59: C1_V2(COVID-19 cases [N=3523] vs. lab/self-reported negative [N=36634]). The bottom panel shows SNPs from the ACE2-gene network and SNPs highlighted in purple are LD-independent significant SNPs based on the number of LD-independent (r2<0.1) and p-value (<0.01) clumping. The significant SNPs are then annotated for Combined Annotation Dependent Depletion (CADD) scores to signify their pathogenicity (defined as score of >=10). The SNPs are also annotated for gene expression (eQTL) and methylation (mQTL) associations. The genes whose expression is associated is labelled next to the data point. The x-axis shows genomic coordinate of the SNPs grouped by chromosome, also presented as chromosome maps. The y-axis shows -log10 of the p-value or the CADD score.


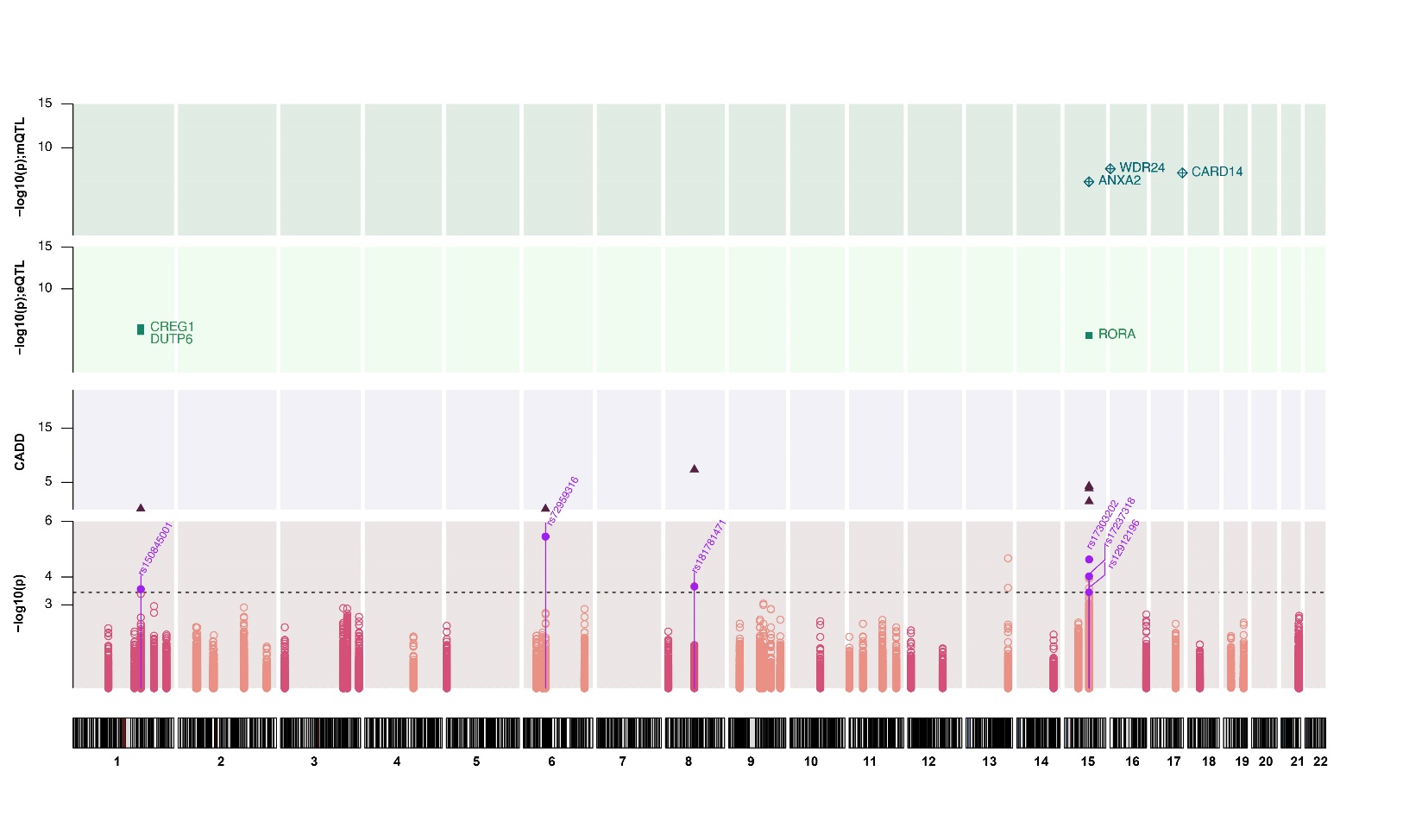


Figure S60: C2_V2(COVID-19 cases [N=6696] vs. population [N=1073072]). The bottom panel shows SNPs from the ACE2-gene network and SNPs highlighted in purple are LD-independent significant SNPs based on the number of LD-independent (r2<0.1) and p-value (<0.01) clumping. The significant SNPs are then annotated for Combined Annotation Dependent Depletion (CADD) scores to signify their pathogenicity (defined as score of >=10). The SNPs are also annotated for gene expression (eQTL) and methylation (mQTL) associations. The genes whose expression is associated is labelled next to the data point. The x-axis shows genomic coordinate of the SNPs grouped by chromosome, also presented as chromosome maps. The y-axis shows -log10 of the p-value or the CADD score.


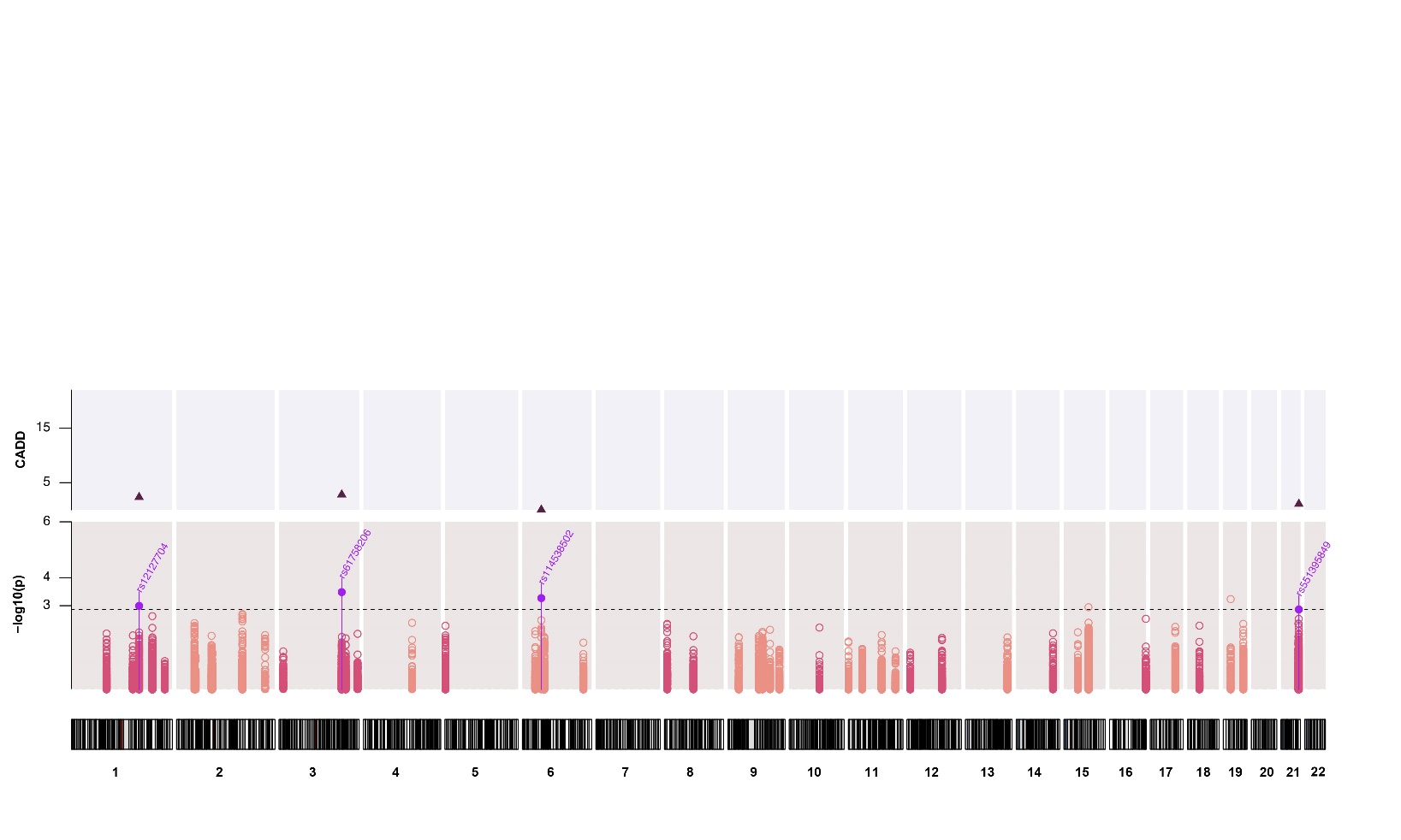


Figure S61: D1_V2 (predicted COVID-19 cases from self-reported symptoms [N=1865] vs. predicted or self-reported non-COVID-19 cases [N=29174]). The bottom panel shows SNPs from the ACE2-gene network and SNPs highlighted in purple are LD-independent significant SNPs based on the number of LD-independent (r2<0.1) and p-value (<0.01) clumping. The significant SNPs are then annotated for Combined Annotation Dependent Depletion (CADD) scores to signify their pathogenicity (defined as score of >=10). The SNPs are also annotated for gene expression (eQTL) and methylation (mQTL) associations. The genes whose expression is associated is labelled next to the data point. The x-axis shows genomic coordinate of the SNPs grouped by chromosome, also presented as chromosome maps. The y-axis shows -log10 of the p-value or the CADD score.
